# Supplementary material for: Exploring the Synthetic Potential of γ-Lactam Derivatives Obtained from a Multicomponent Reaction—Applications as Antiproliferative Agents
Source: Molecules. 2022 Jun 5;27(11):3624. doi: 10.3390/molecules27113624 (PMC9182551; doi:10.3390/molecules27113624)
Supplement: Supplementary file 1 [file molecules-27-03624-s001.zip › molecules-1754758-supplementary.pdf]

Supporting Information

Exploring the Synthetic Potential of γ-Lactam Derivatives Obtained from a Multicomponent Reaction. Applications as Antiproliferative Agents.

Adrián López-Francés, Xabier del Corte, Zuriñe Serna-Burgos, Edorta Martínez de Marigorta, Francisco Palacios* and Javier Vicario*

**Departamento de Química Orgánica I, Centro de Investigación y Estudios Avanzados "Lucio Lascaray"- Facultad de Farmacia, University of the Basque Country, UPV/EHU Paseo de la Universidad 7, 01006 Vitoria-Gasteiz, SPAIN.*

E mail: francisco.palacios@ehu.eus / javier.vicario@ehu.eus

Table of contents

1. Characterization by NMR Spectroscopy of products **7-10**. **S2**
2. Characterization by NMR Spectroscopy and Crystal Structure Determination of products **12**. **S21**
3. Characterization by NMR Spectroscopy and Crystal Structure Determination of product **13**. **S27**
4. Characterization by NMR Spectroscopy of products **18** and **19**. **S31**
5. Characterization by NMR Spectroscopy of product **20**. **S35**
6. Characterization by NMR Spectroscopy of products **21** and **22**. **S42**
7. Characterization by NMR Spectroscopy of products **25**. **S46**
8. Calculation of Lipinski's rule of five and prediction of ADME properties. **S53**

1. Characterization by NMR Spectroscopy of products 7-10.

Methyl (*E*)-3-(5-oxo-1-(*p*-tolyl)-4-(*p*-tolylamino)-2,5-dihydro-1*H*-pyrrol-3-yl)acrylate (7a).

^{1}H NMR (400 MHz, $CDCl_3$)

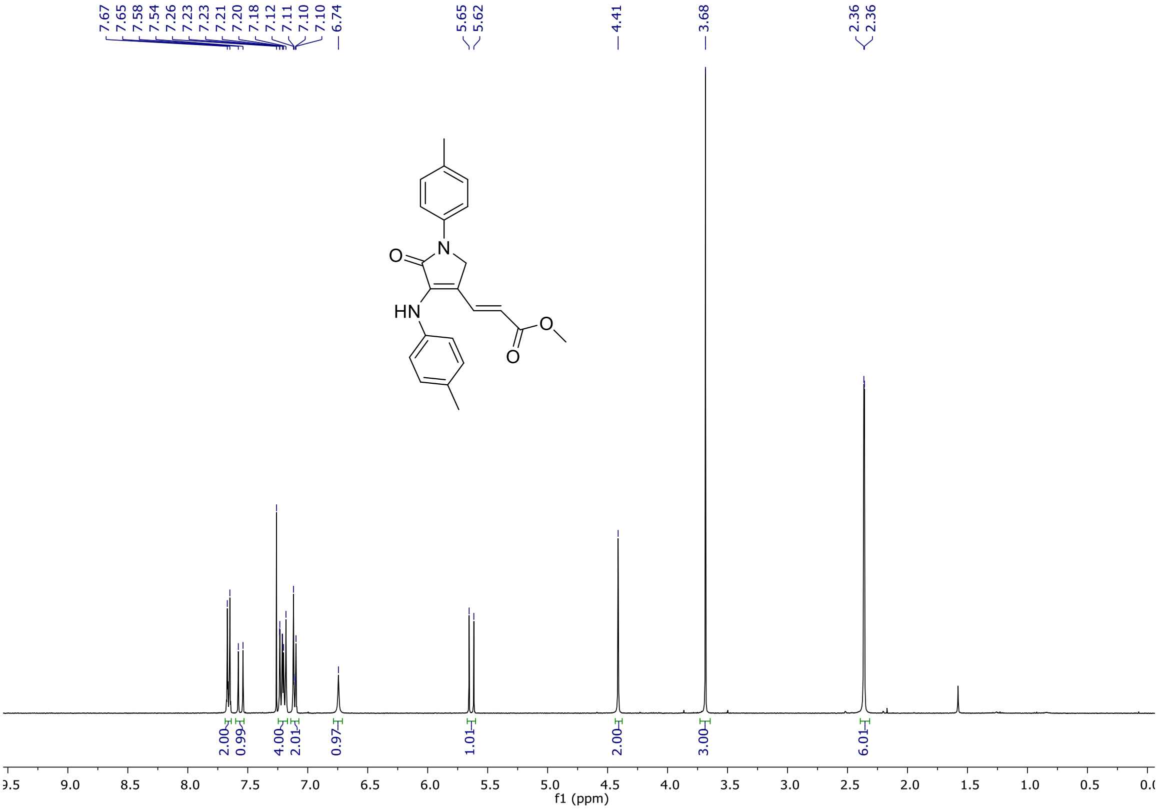

^{13}C NMR {^{1}H} (101 MHz, $CDCl_3$)

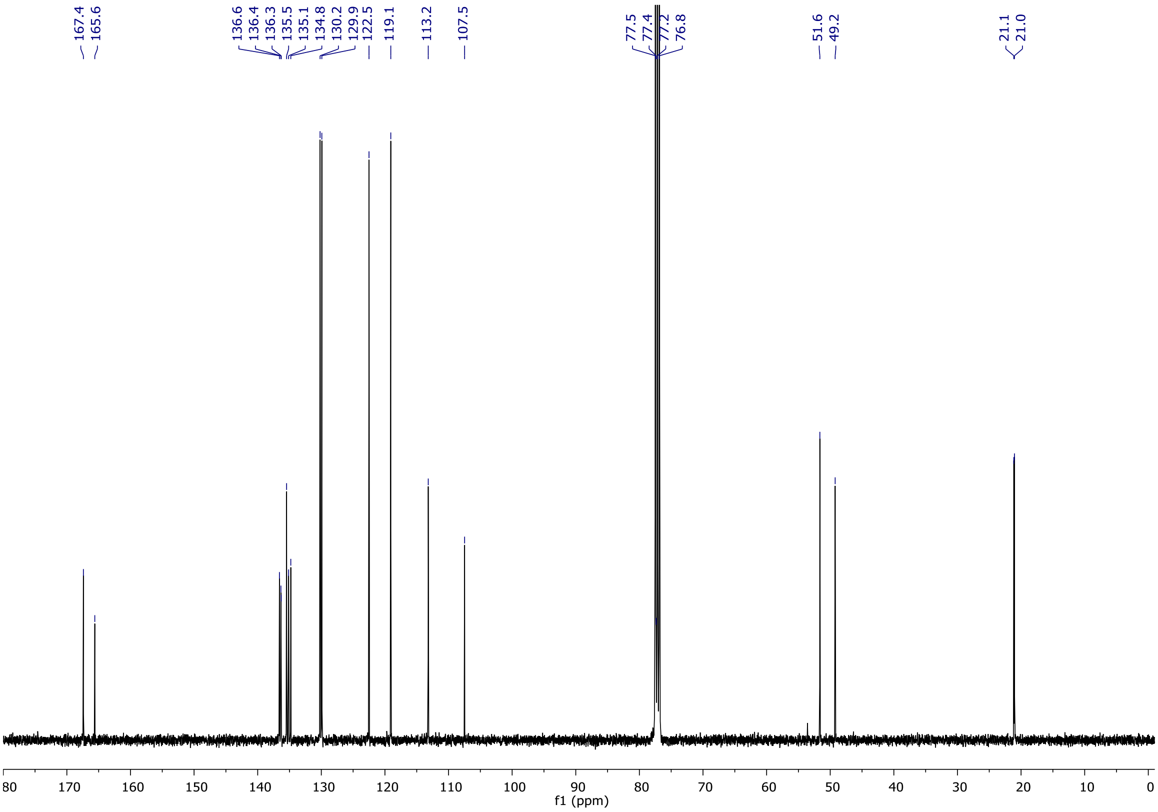

2D-COSY NMR {^{1}H – ^{1}H} (400 MHz, $CDCl_3$)

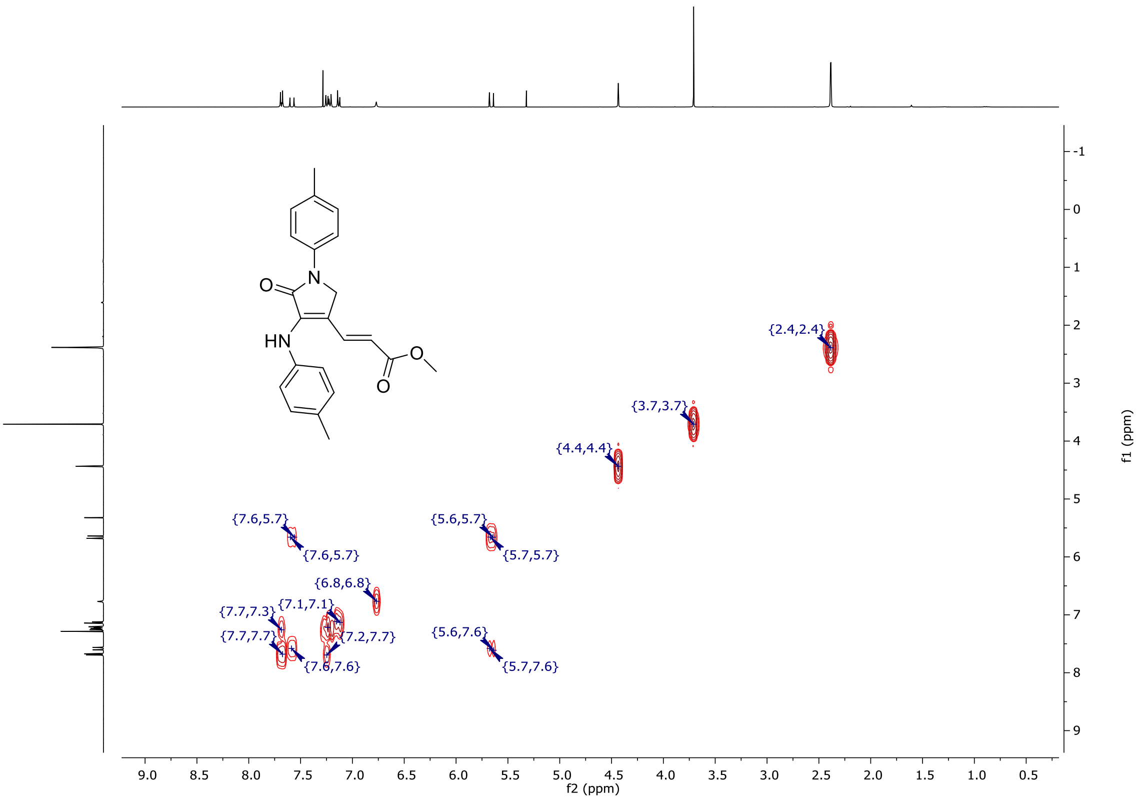

2D-HSQC NMR {^{1}H – ^{13}C} (^{1}H: 400 MHz, ^{13}C: 101 MHz, $CDCl_3$)

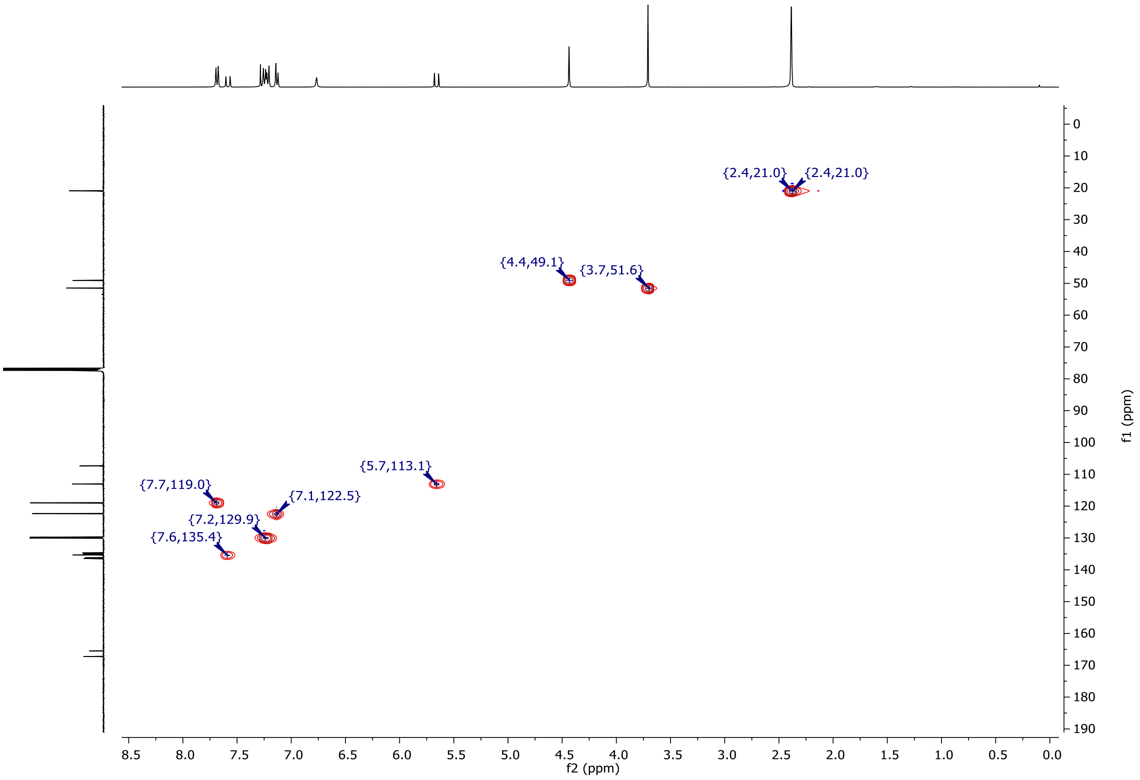

2D-HMBC NMR {^{1}H – ^{13}C} (^{1}H: 400 MHz, ^{13}C: 101 MHz, $CDCl_3$)

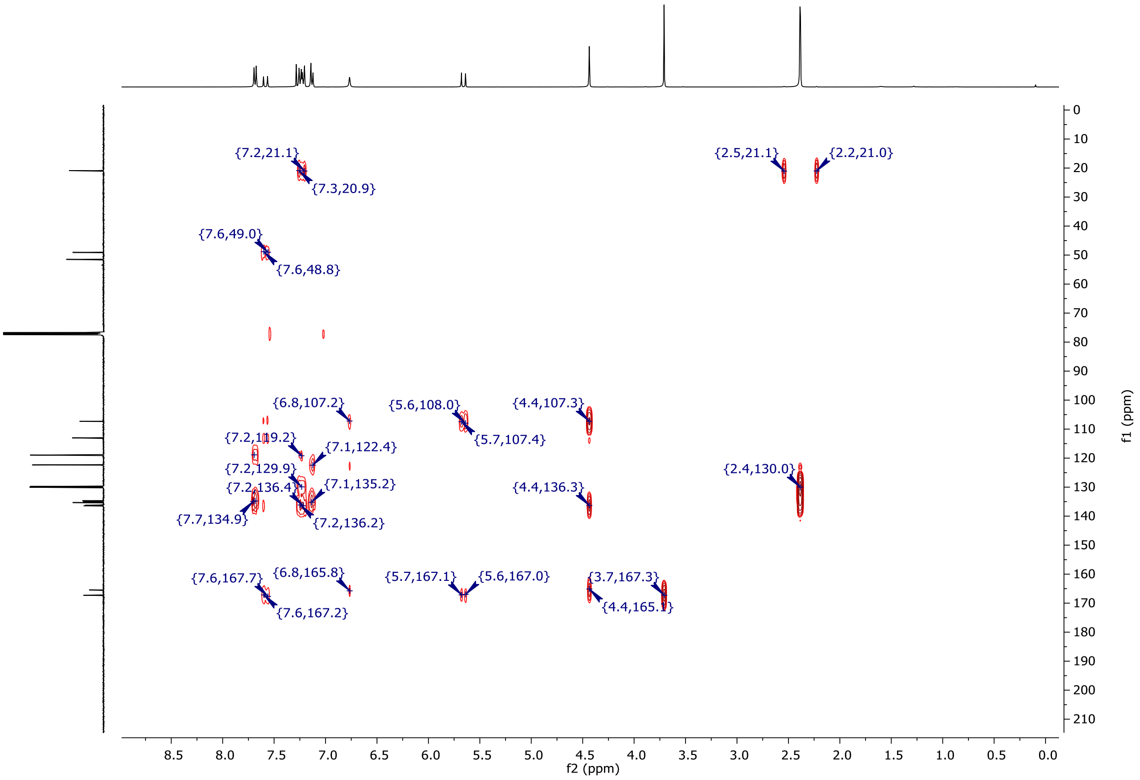

Methyl (*E*)-3-(5-oxo-1-(3-(trifluoromethyl)phenyl)-4-((3-(trifluoromethyl)phenyl)amino)-2,5-dihydro-1*H*-pyrrol-3-yl)acrylate (7b).

^{1}H NMR (400 MHz, $CDCl_3$)

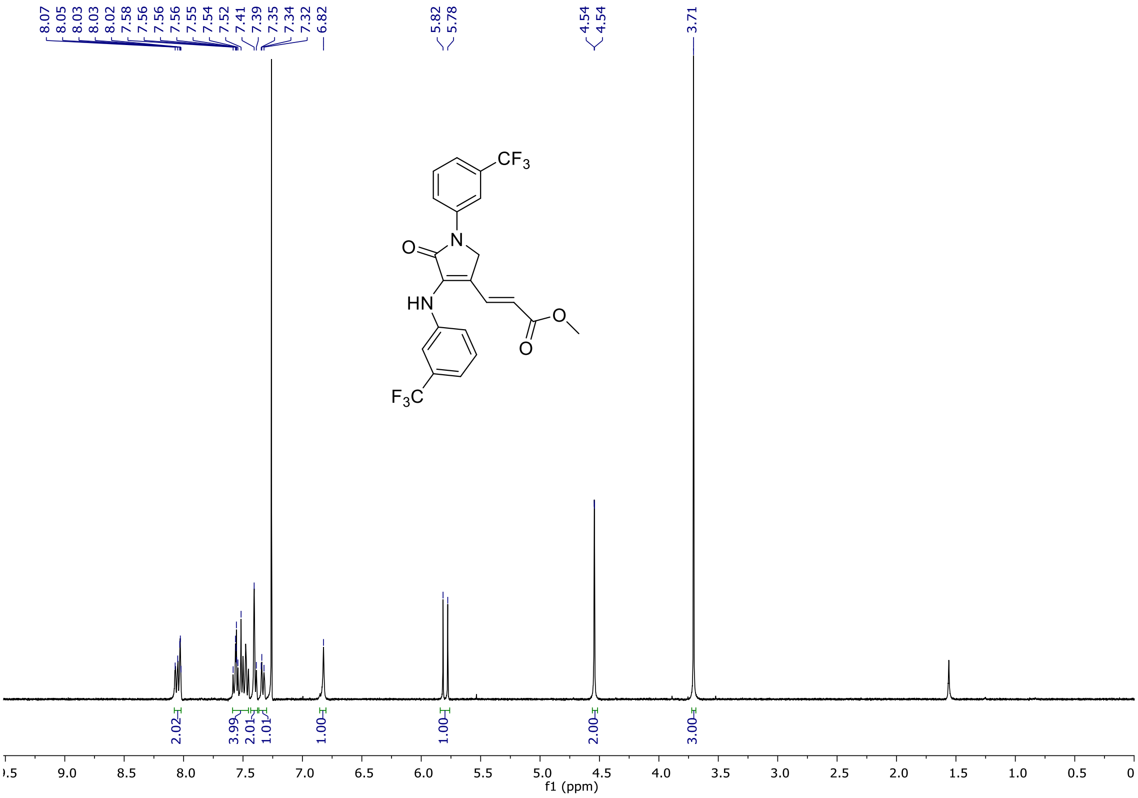

^{13}C NMR {^{1}H} (101 MHz, Acetone d_6)

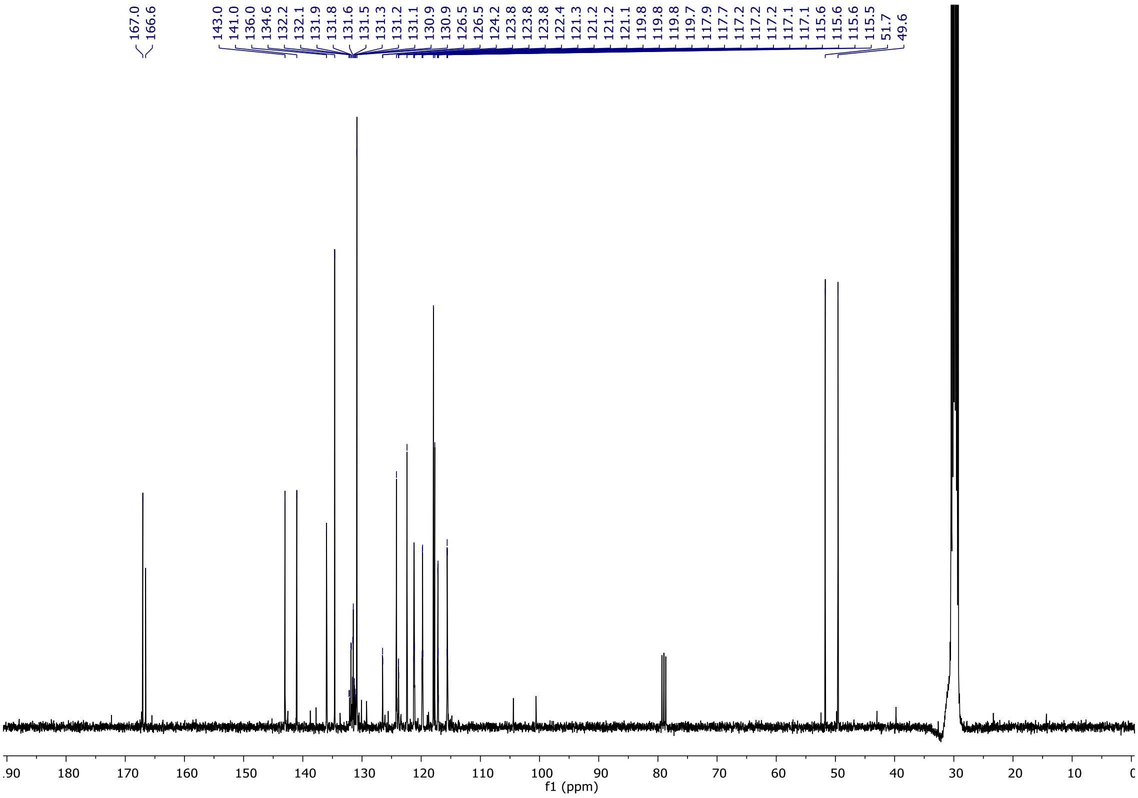

^{19}F-NMR (282 MHz, $CDCl_3$)

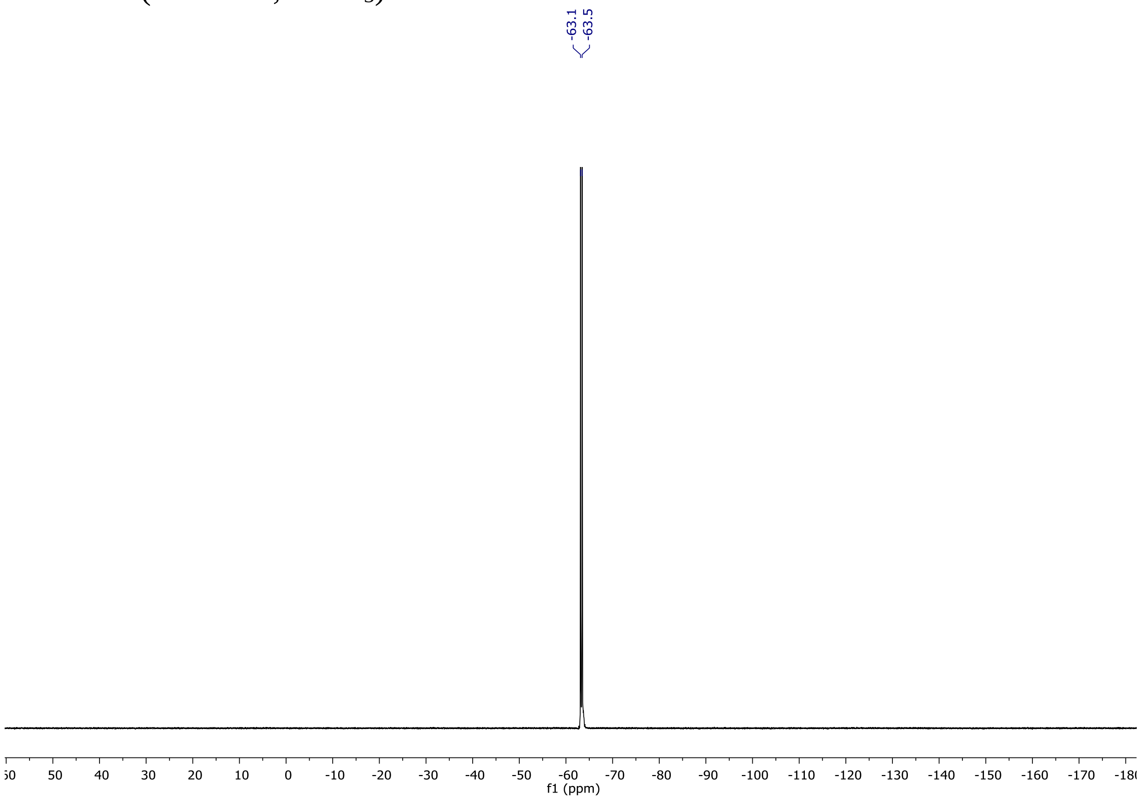

Ethyl (*E*)-3-(5-oxo-1-(p-tolyl)-4-(p-tolylamino)-2,5-dihydro-1*H*-pyrrol-3-yl)acrylate (7c).

^{1}H NMR (400 MHz, $CDCl_3$)

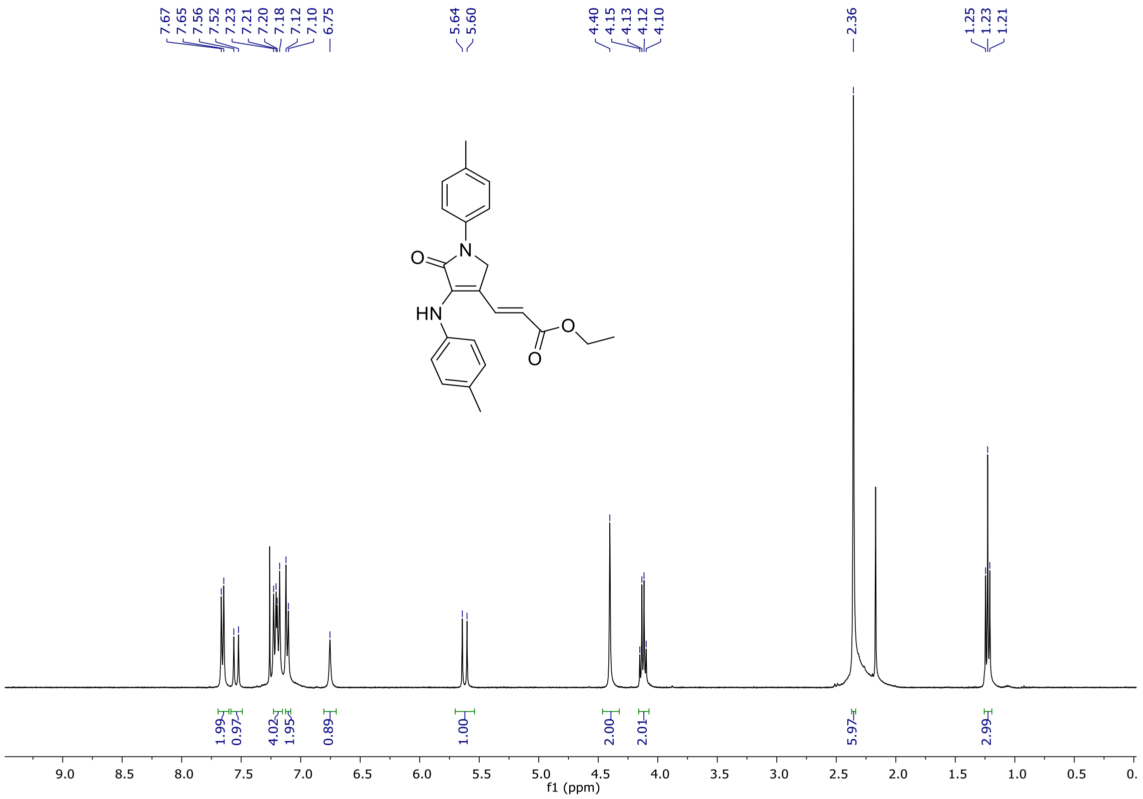

^{13}C NMR {^{1}H} (101 MHz, $CDCl_3$)

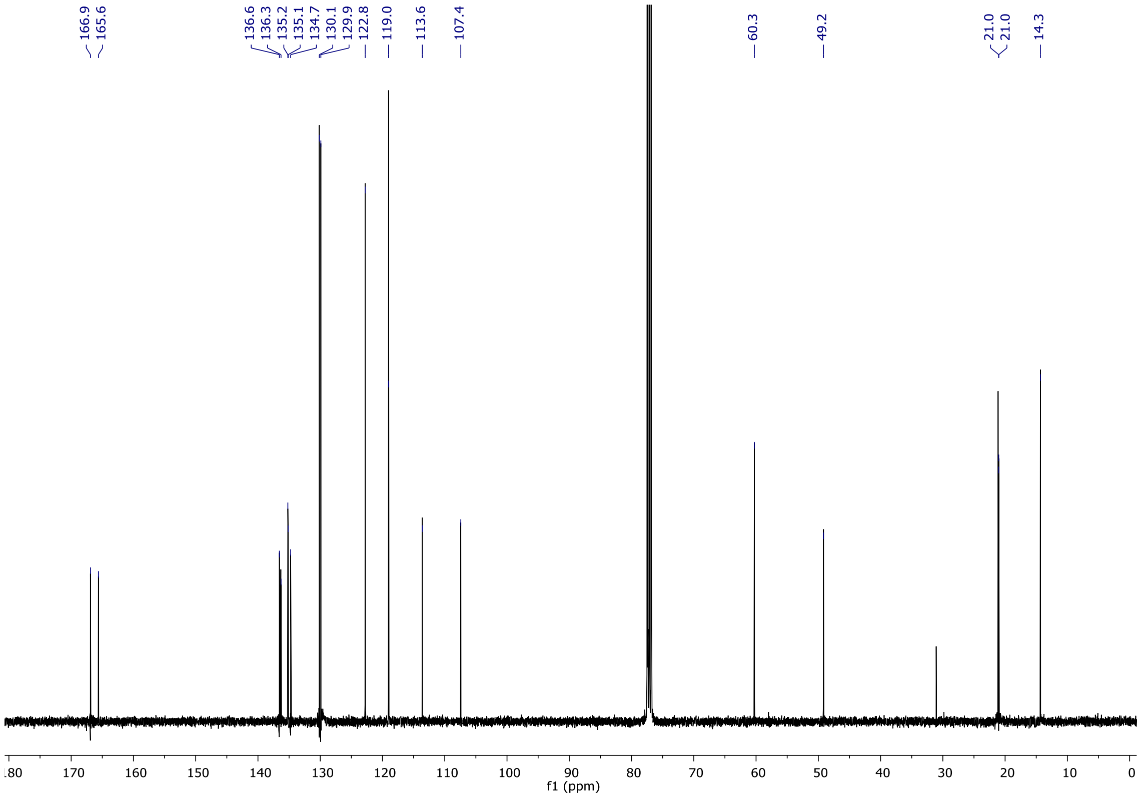

Tert-butyl (*E*)-3-(5-oxo-1-(p-tolyl)-4-(p-tolylamino)-2,5-dihydro-1*H*-pyrrol-3-yl)acrylate (7d).

^{1}H NMR (400 MHz, $CDCl_3$)

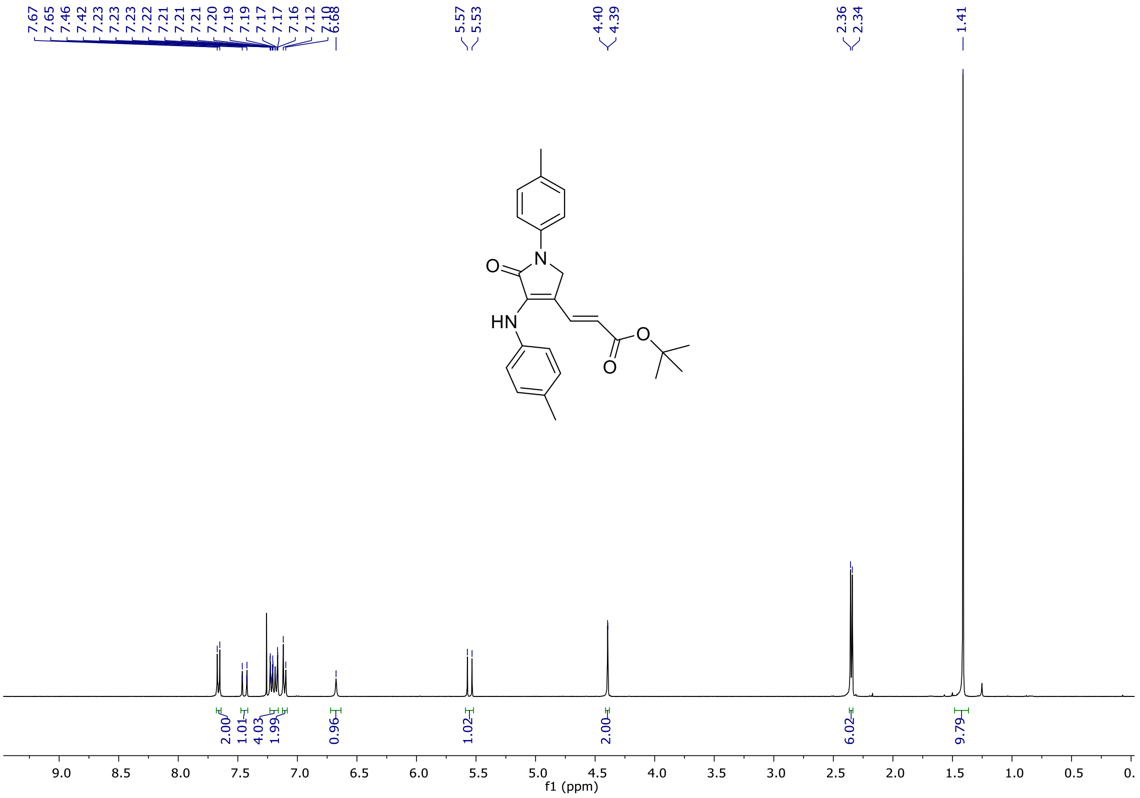

^{13}C NMR {^{1}H} (101 MHz, $CDCl_3$)

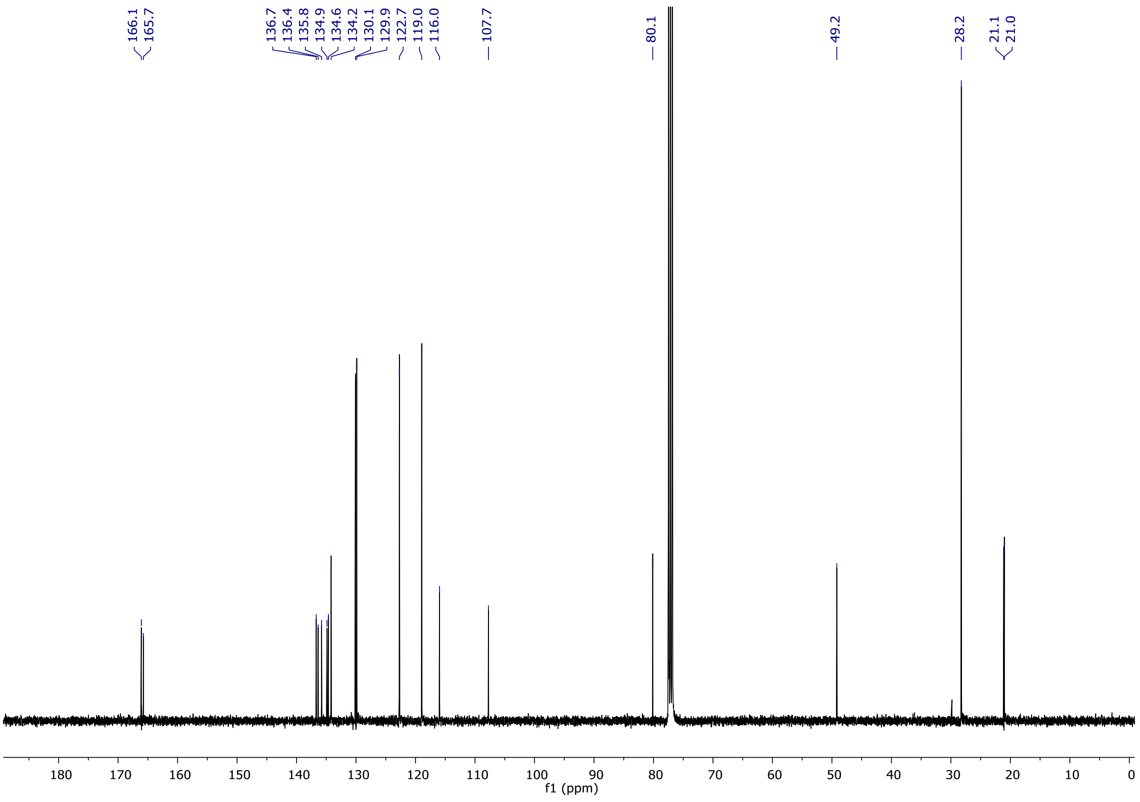

Tert-butyl (*E*)-3-(5-oxo-1-(3-(trifluoromethyl)phenyl)-4-((3-(trifluoromethyl)phenyl)amino)-2,5-dihydro-1*H*-pyrrol-3-yl)acrylate (7e).

^{1}H NMR (400 MHz, $CDCl_3$)

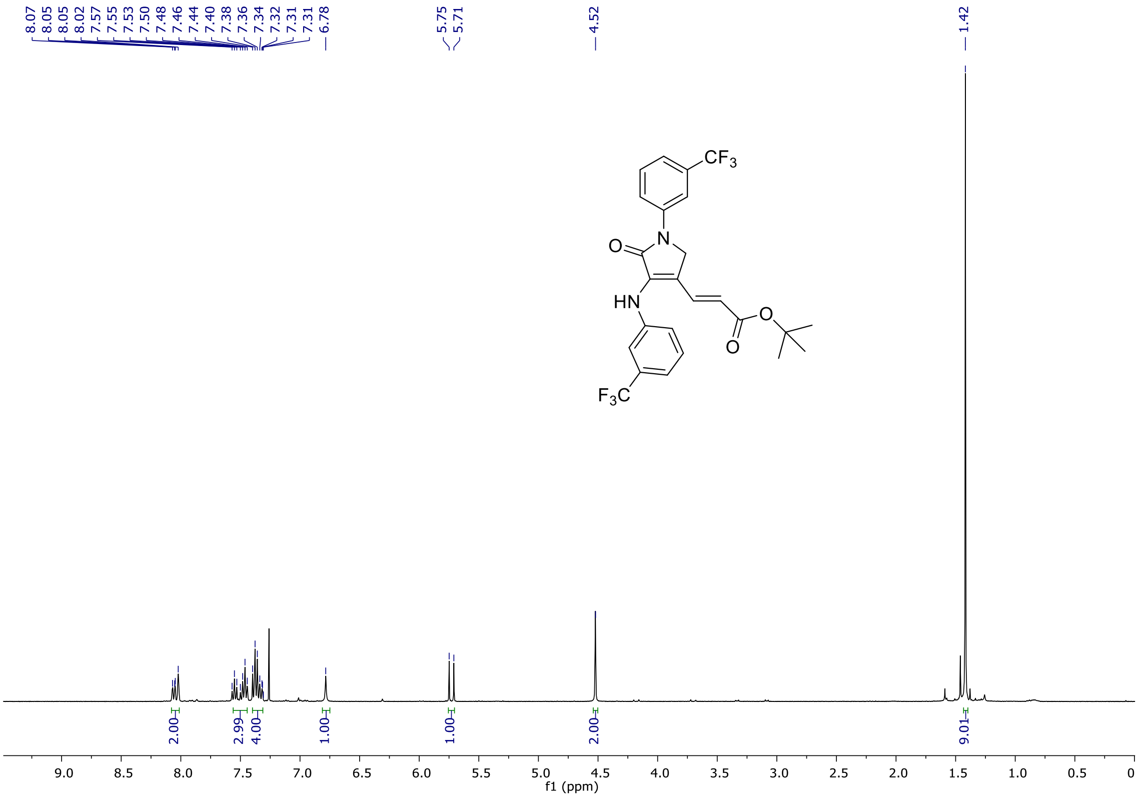

^{13}C NMR {^{1}H} (75 MHz, $CDCl_3$)

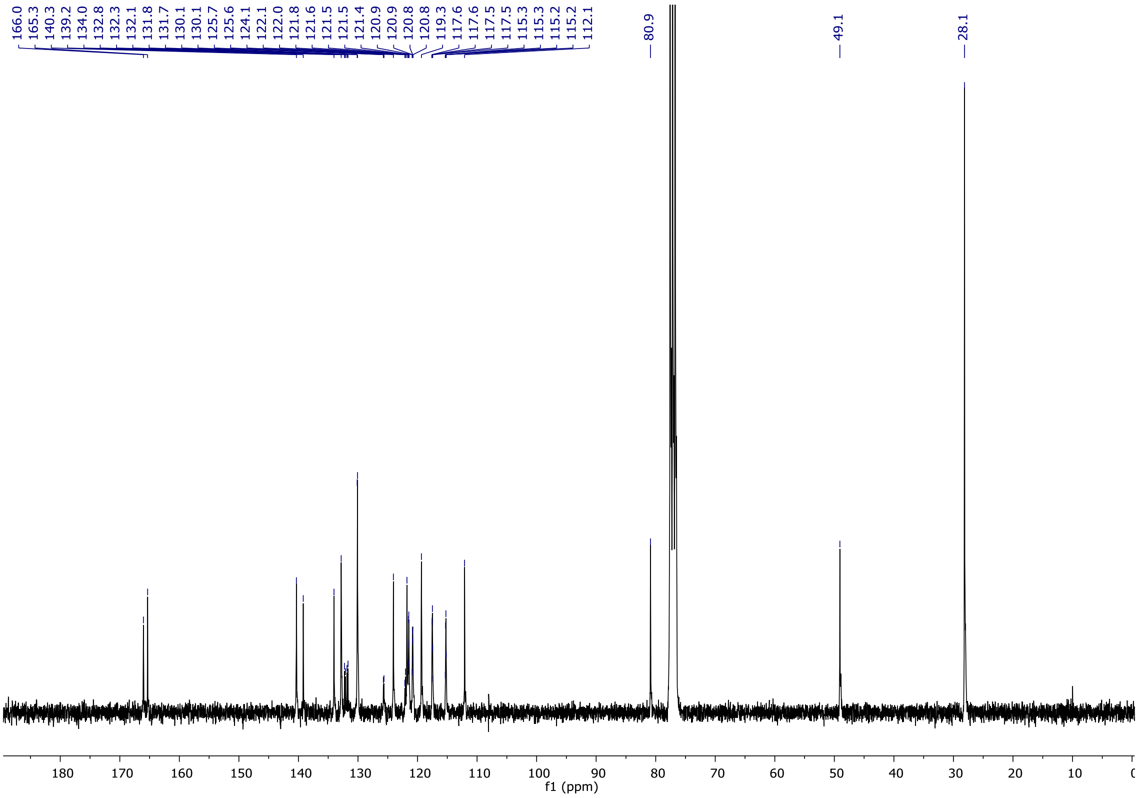

^{19}F-NMR (282 MHz, $CDCl_3$)

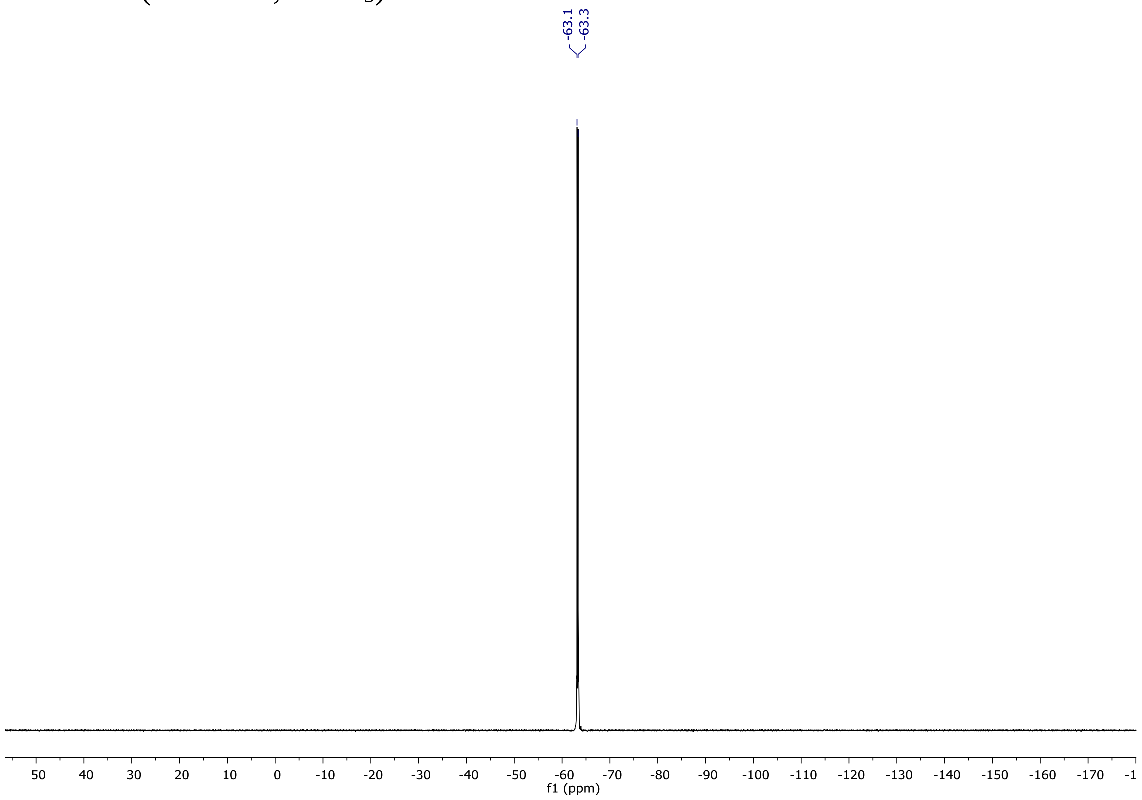

Naphthalen-2-yl (*E*)-3-(5-oxo-1-(*p*-tolyl)-4-(*p*-tolylamino)-2,5-dihydro-1*H*-pyrrol-3-yl)acrylate (7f).

^{1}H NMR (400 MHz, $CDCl_3$)

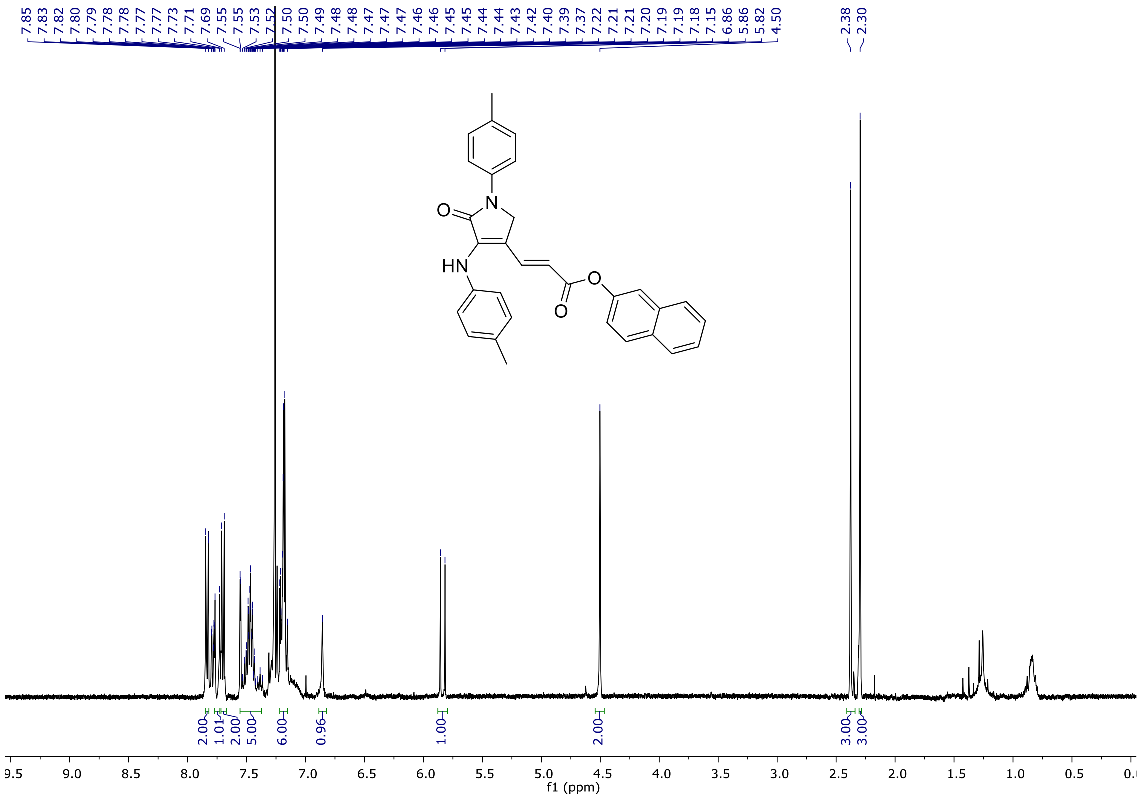

^{13}C NMR {^{1}H} (101 MHz, $CDCl_3$)

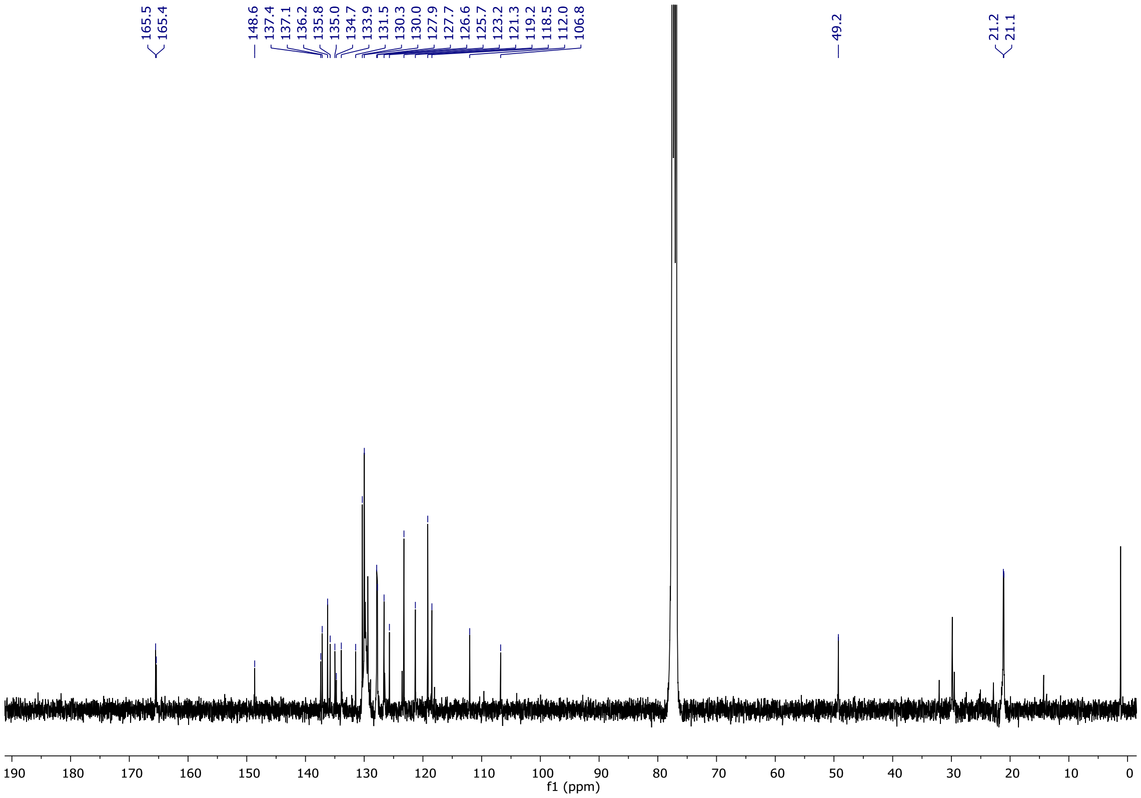

(*E*)-4-(3-oxobut-1-en-1-yl)-1-(*p*-tolyl)-3-(*p*-tolylamino)-1,5-dihydro-2*H*-pyrrol-2-one (7g).

^{1}H NMR (300 MHz, $CDCl_3$)

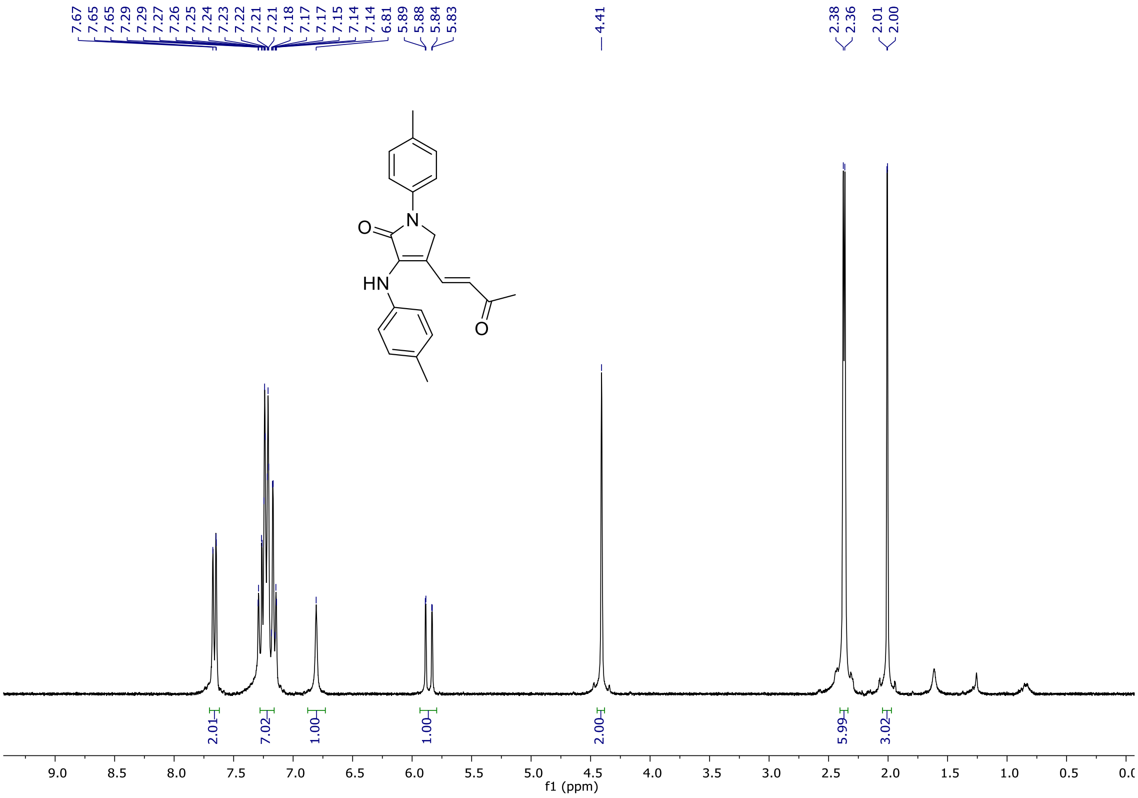

^{13}C NMR {^{1}H} (75 MHz, $CDCl_3$)

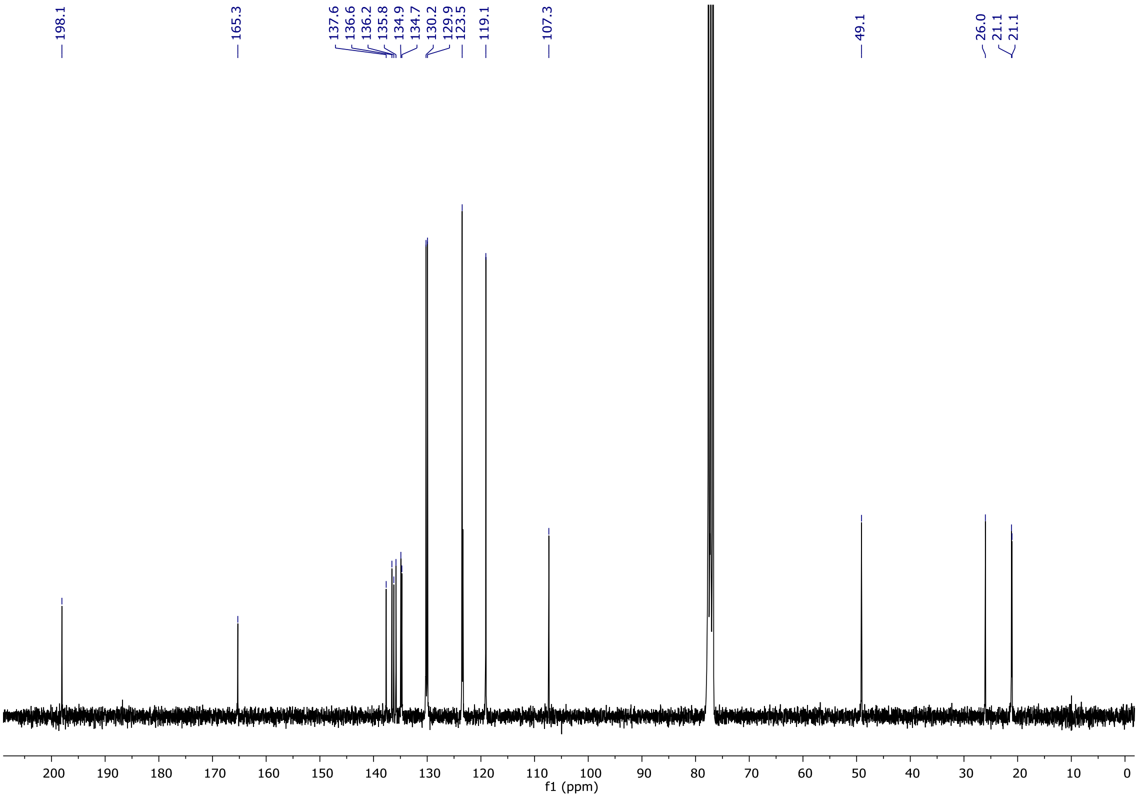

(*E*)-4-(3-oxobut-1-en-1-yl)-1-(3-(trifluoromethyl)phenyl)-3-((3-(trifluoromethyl)phenyl)amino)-1,5-dihydro-2*H*-pyrrol-2-one (6h).

^{1}H NMR (400 MHz, Acetone d_6)

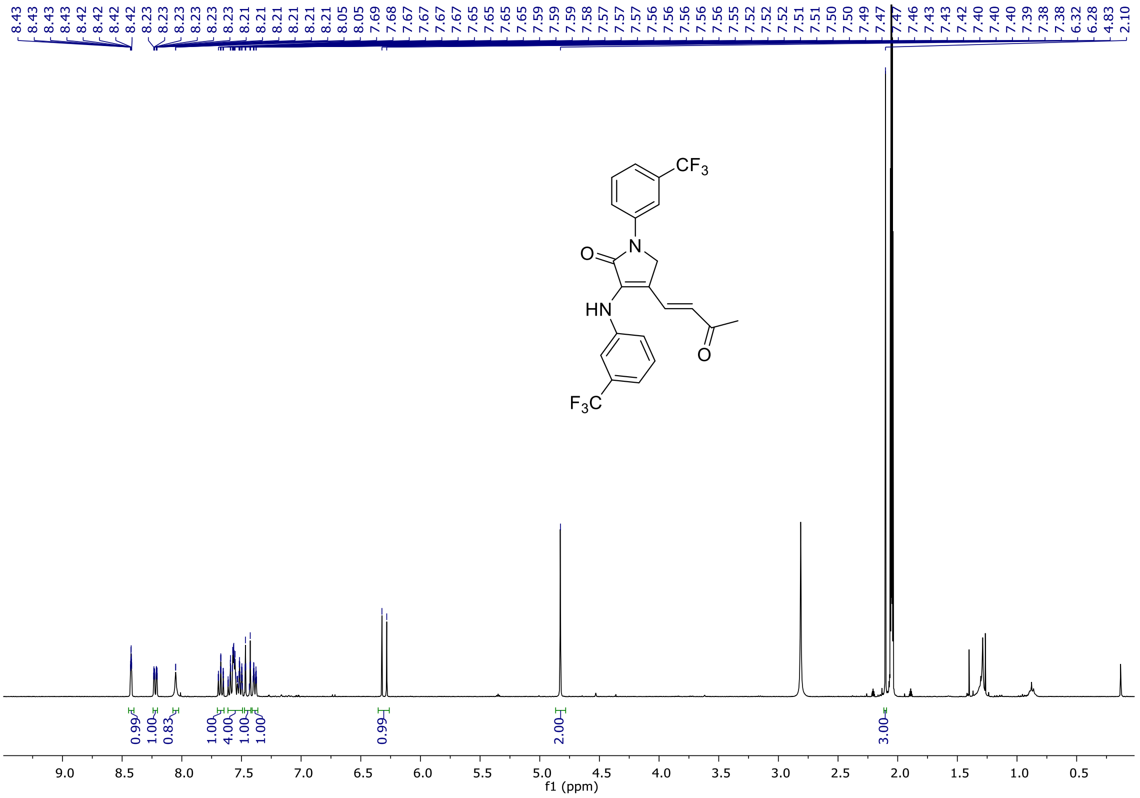

^{13}C NMR {^{1}H} (101 MHz, Acetone d_6)

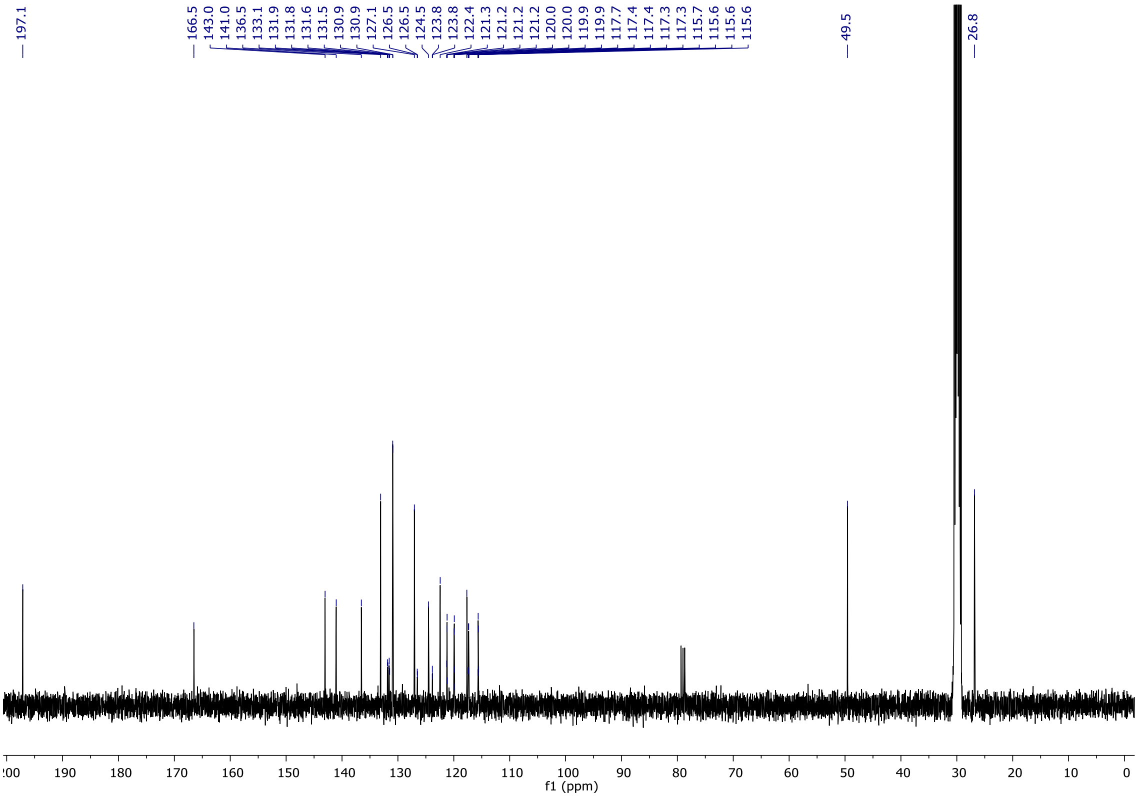

^{19}F NMR (282 MHz, $CDCl_3$)

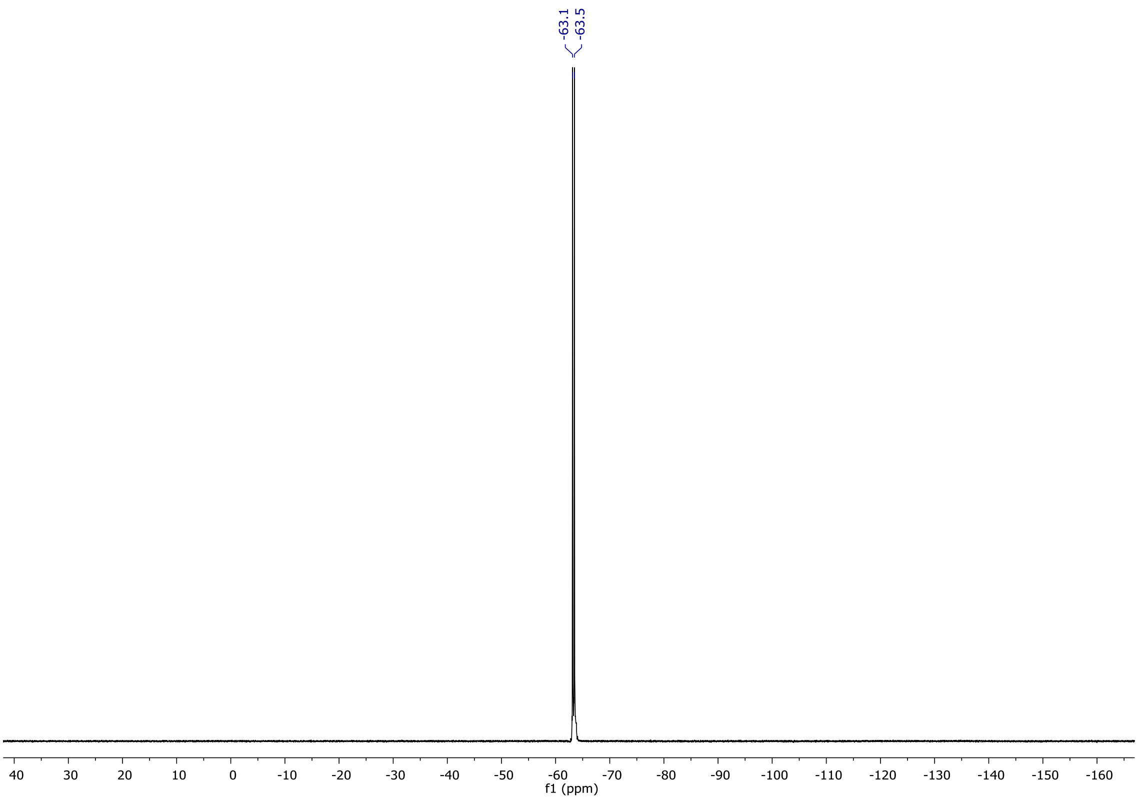

Methyl 3-((3*R,4*R**)-5-oxo-1-(*p*-tolyl)-4-(*p*-tolylamino)pyrrolidin-3-yl)propanoate (8).**

^{1}H NMR (400 MHz, $CDCl_3$)

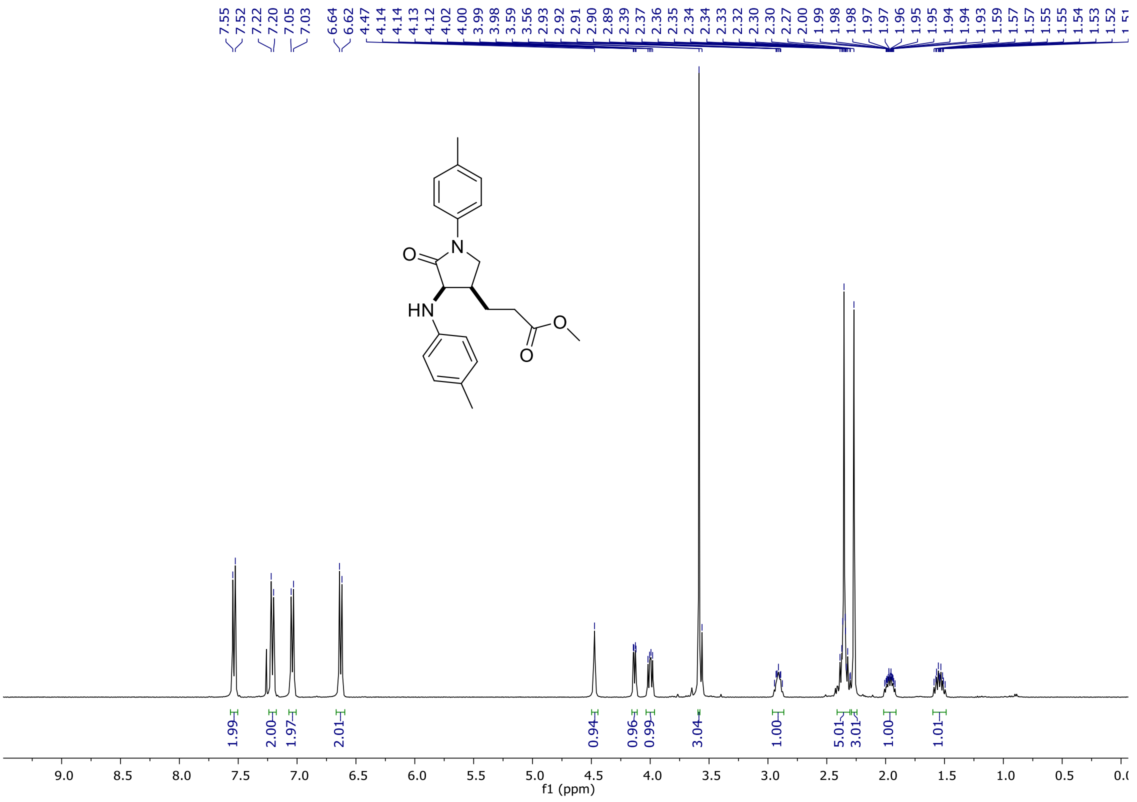

^{13}C NMR {^{1}H} (101 MHz, $CDCl_3$)

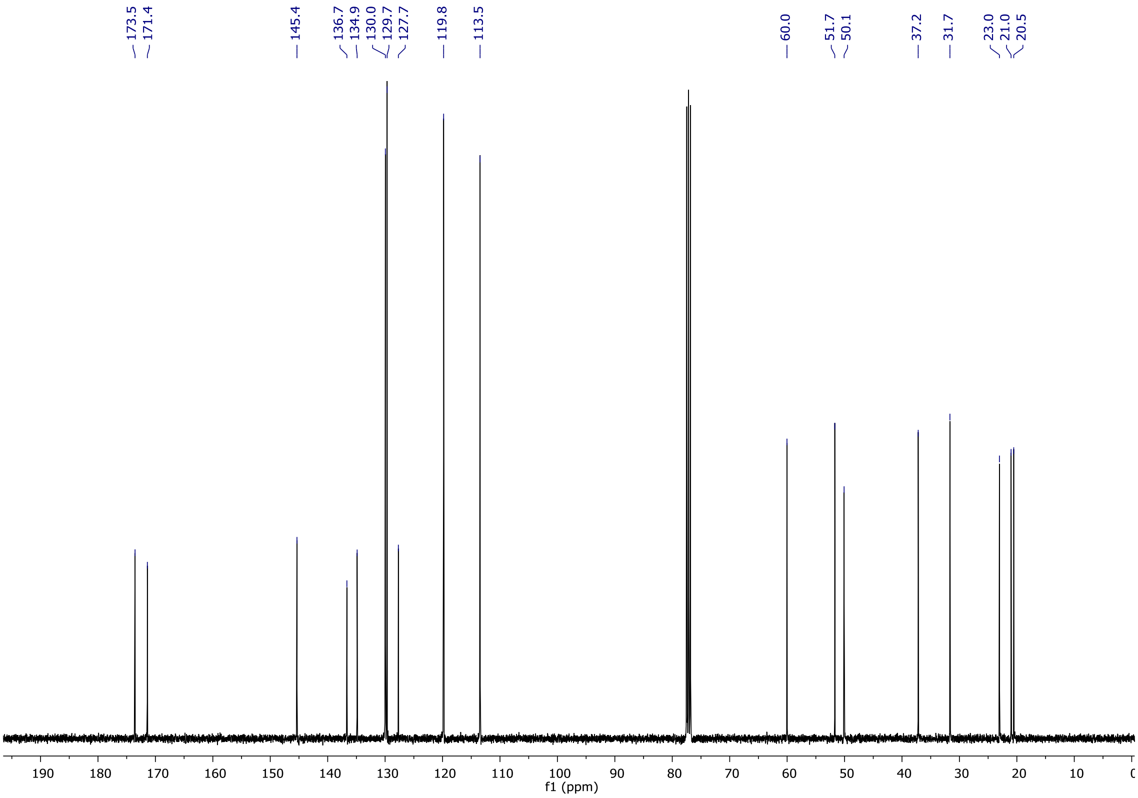

1D-NOESY {^{1}H – ^{1}H} (400 MHz, $CDCl_3$)

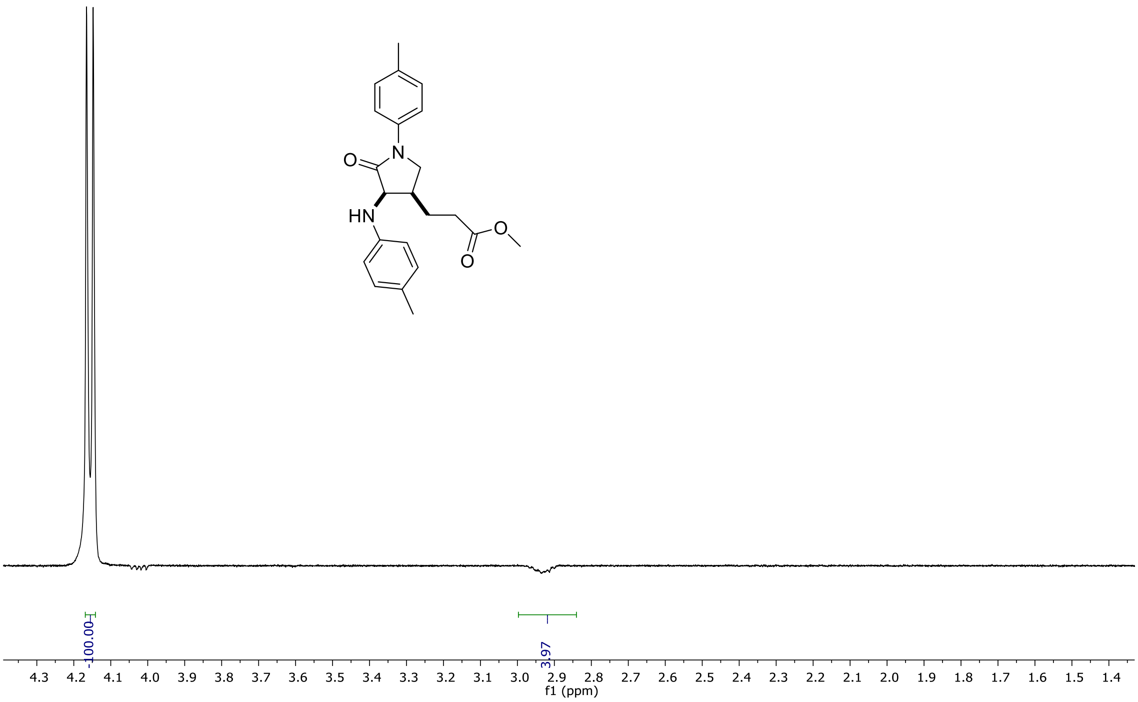

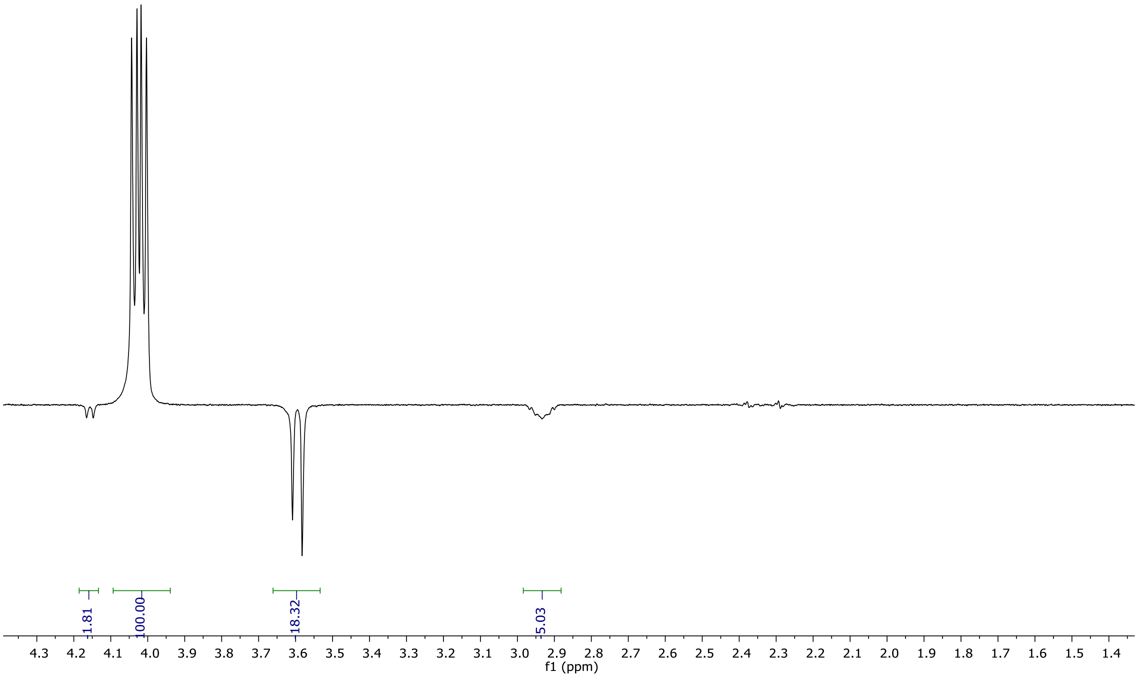

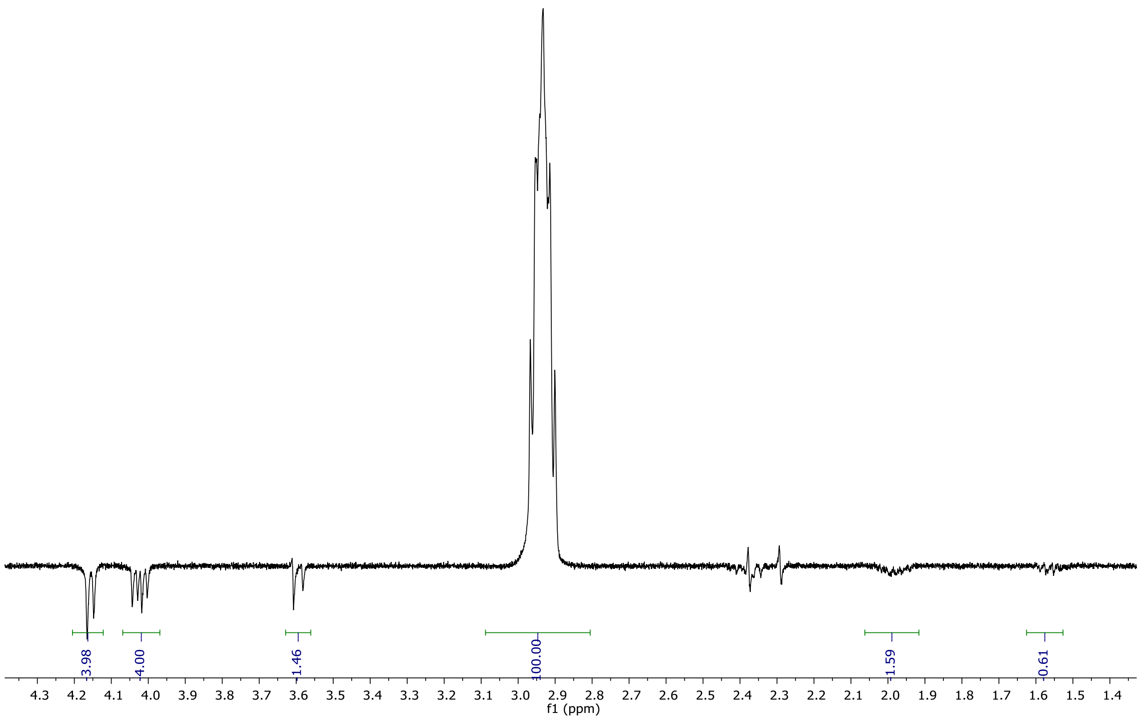
3.98
4.00
1.46
100.00
1.59
0.61
4.3 4.2 4.1 4.0 3.9 3.8 3.7 3.6 3.5 3.4 3.3 3.2 3.1 3.0 2.9 2.8 2.7 2.6 2.5 2.4 2.3 2.2 2.1 2.0 1.9 1.8 1.7 1.6 1.5 1.4
f1 (ppm)

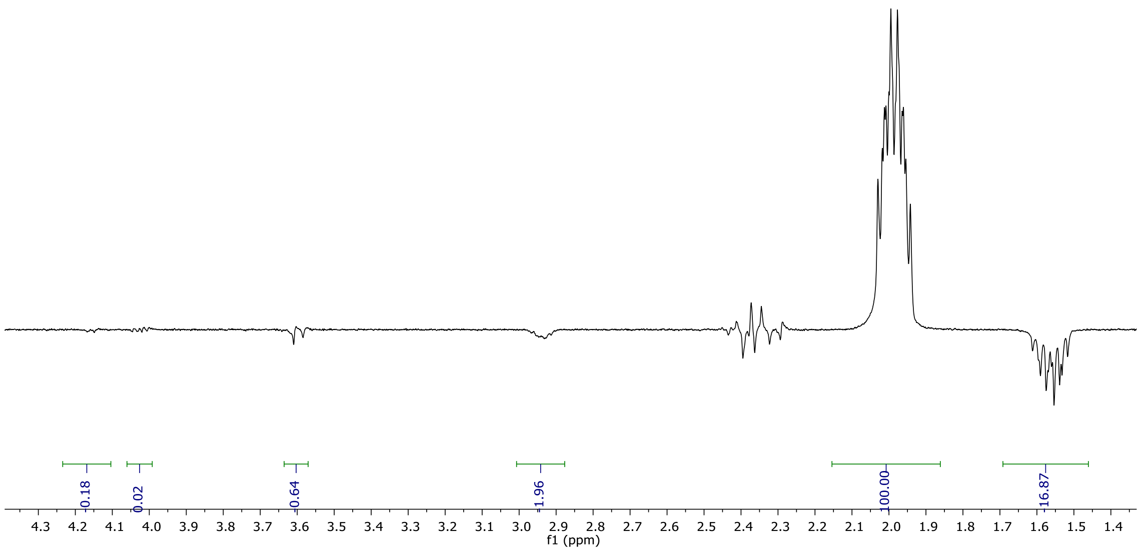
0.18
0.02
0.64
1.96
100.00
16.87
4.3 4.2 4.1 4.0 3.9 3.8 3.7 3.6 3.5 3.4 3.3 3.2 3.1 3.0 2.9 2.8 2.7 2.6 2.5 2.4 2.3 2.2 2.1 2.0 1.9 1.8 1.7 1.6 1.5 1.4
f1 (ppm)

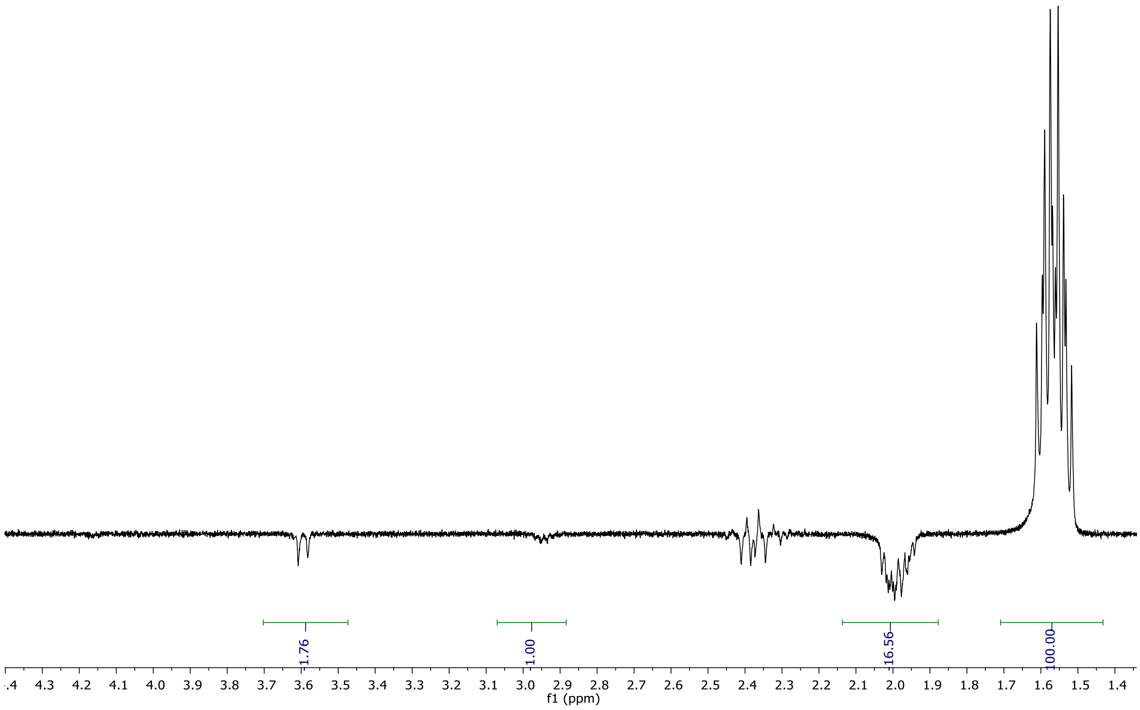
1.76
1.00
16.56
100.00
f1 (ppm)

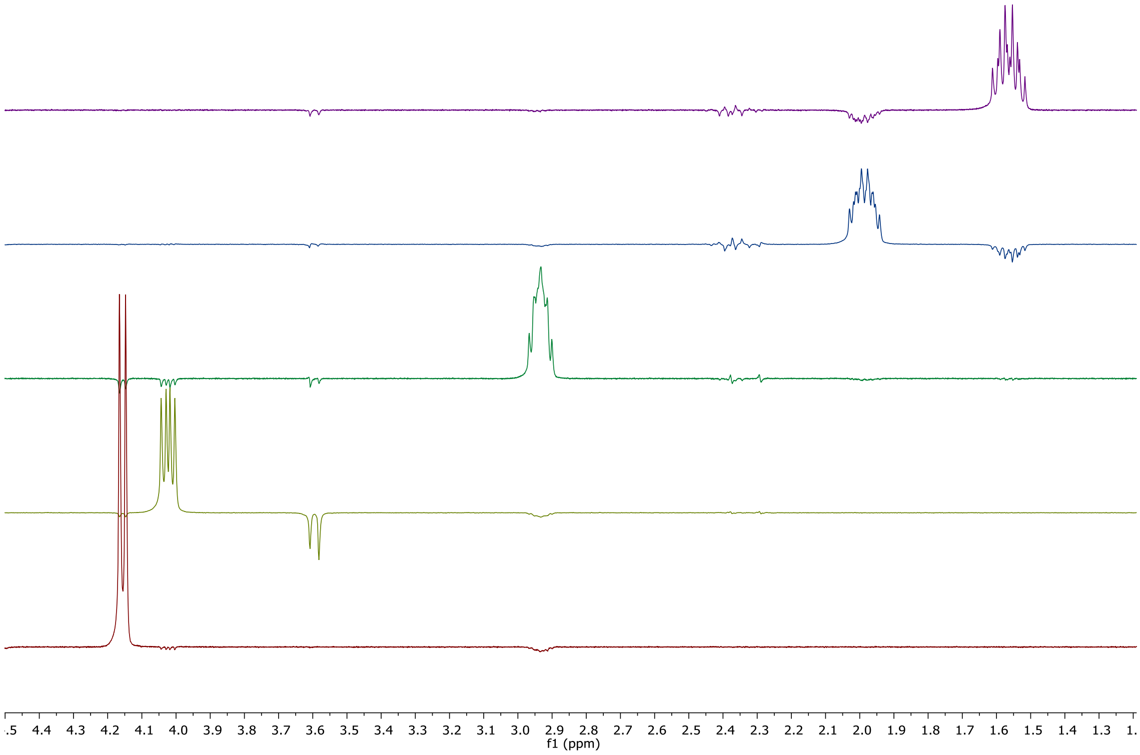
f1 (ppm)

(4a*R,7a*R**)-1,6-Di-*p*-tolylhexahydro-1*H*-pyrrolo[3,4-*b*]pyridine-2,7-dione (9).**

^{1}H NMR (400 MHz, $CDCl_3$)

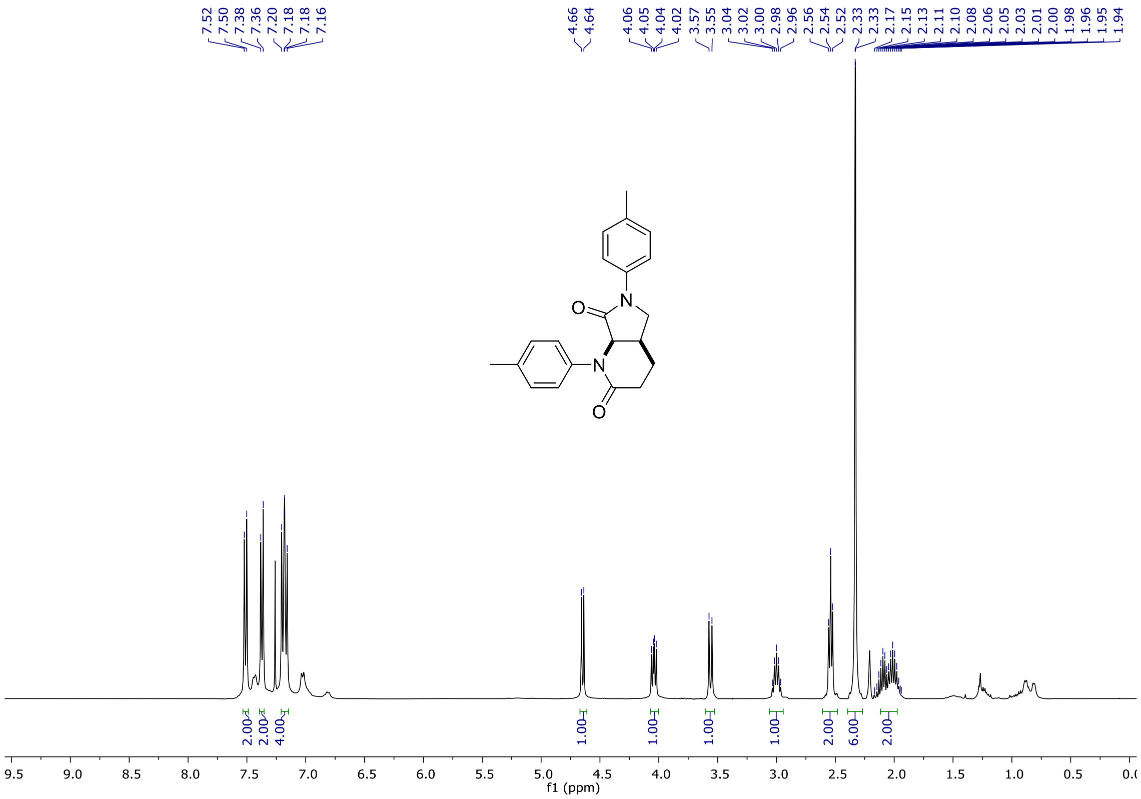

^{13}C NMR {^{1}H} (101 MHz, $CDCl_3$)

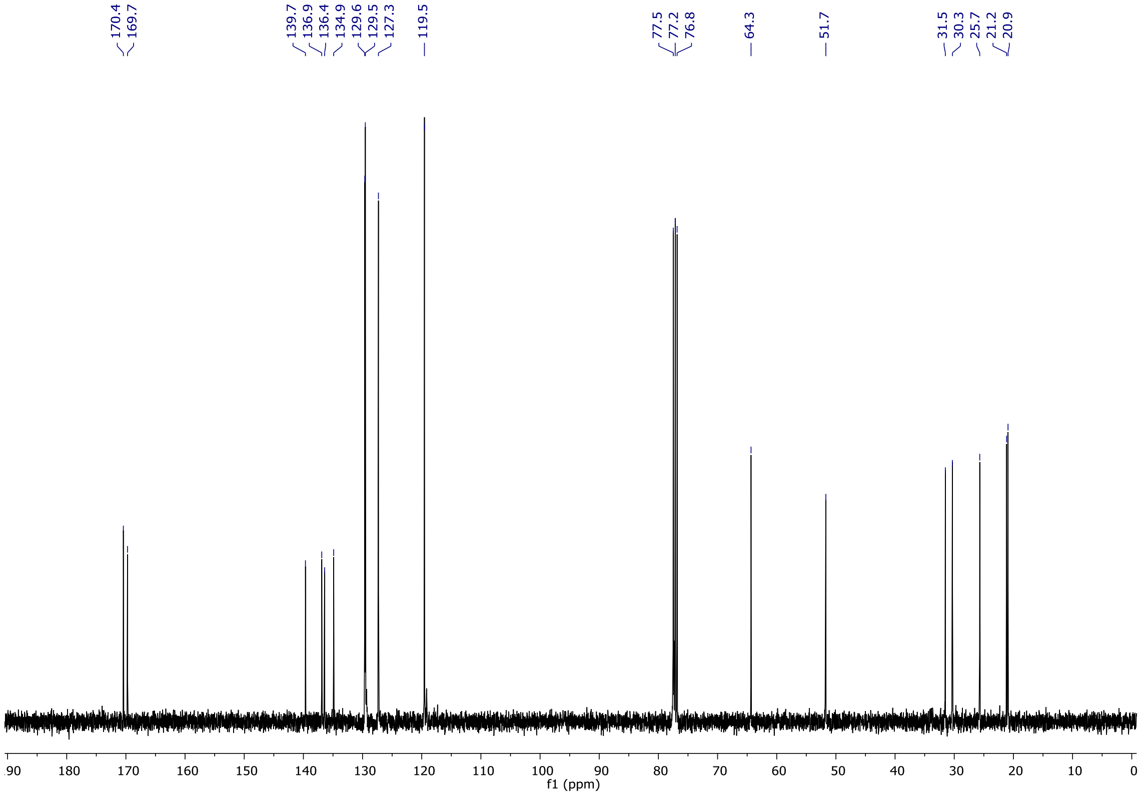

Methyl (*E*)-3-(4-hydroxy-5-oxo-1-(*p*-tolyl)-2,5-dihydro-1*H*-pyrrol-3-yl)acrylate (10).

^{1}H NMR (400 MHz, DMSO-d6)

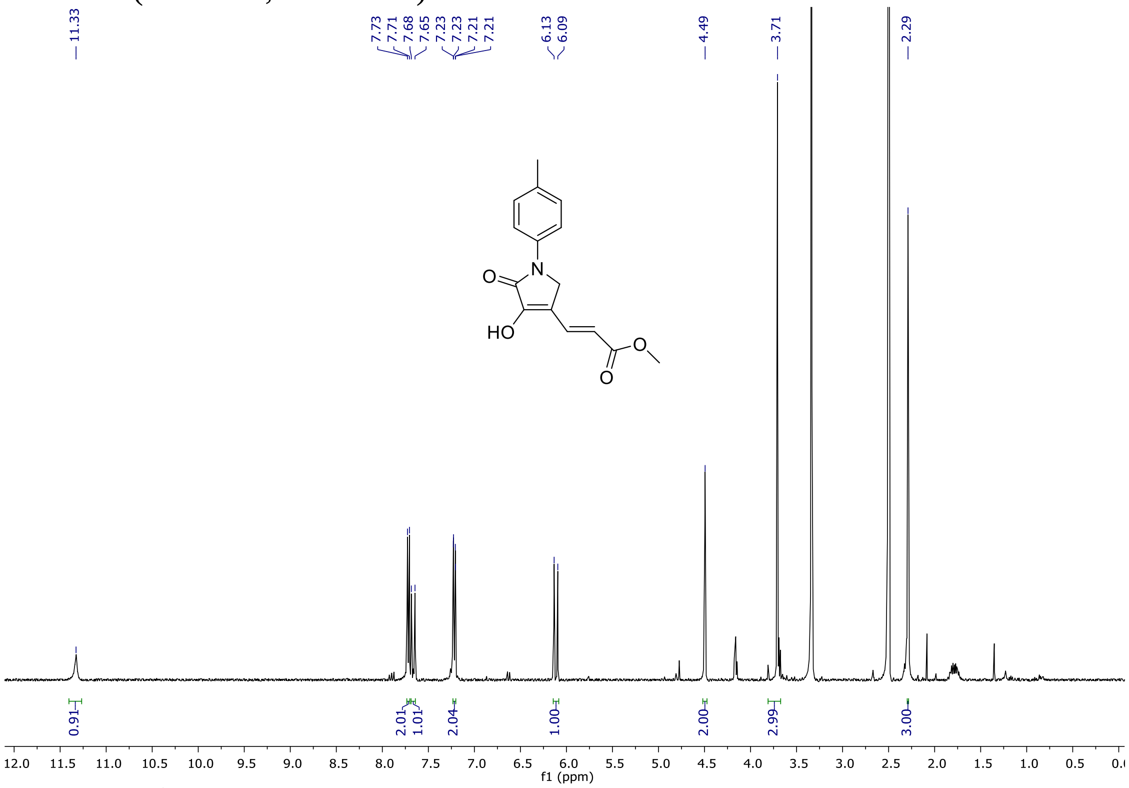

^{13}C NMR {^{1}H} (101 MHz, DMSO-d6)

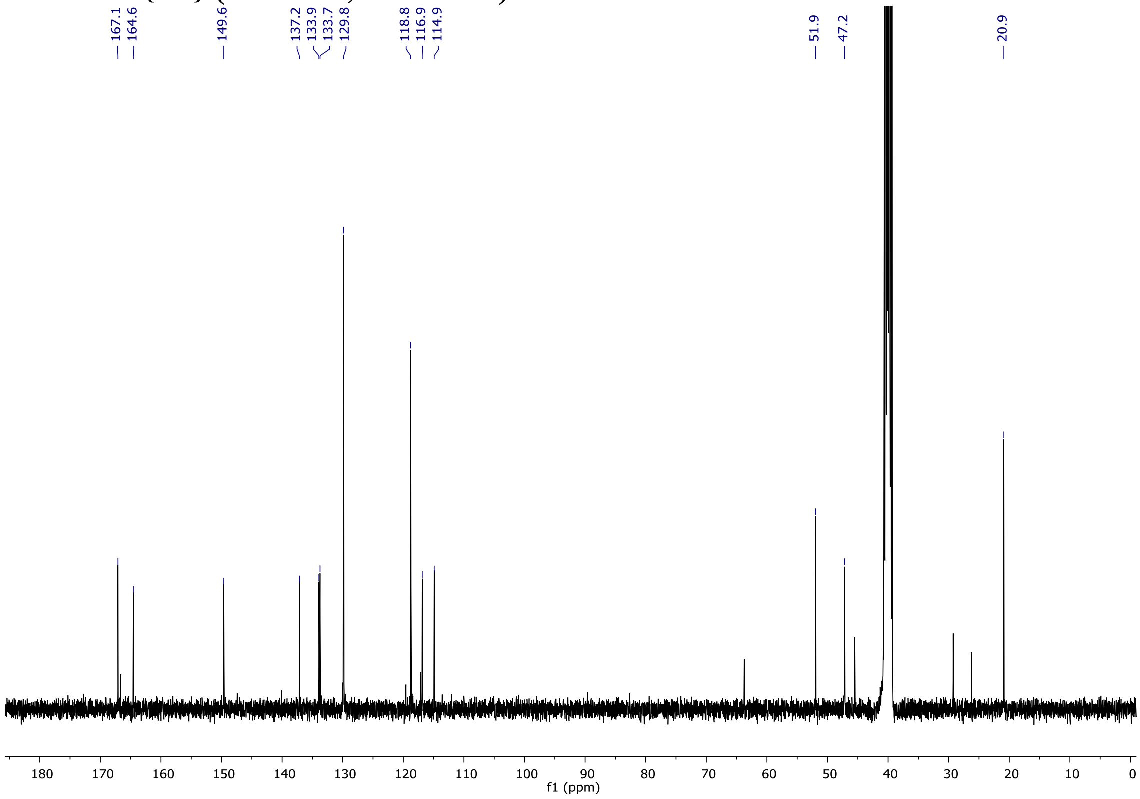

2. Characterization by NMR Spectroscopy and Crystal Structure Determination of products 12.

Ethyl (*R)-2-((*R**)-3-(diethoxyphosphoryl)-5-oxo-1-(*p*-tolyl)-4-(*p*-tolylamino)-2,5-dihydro-1*H*-pyrrol-2-yl)-2-hydroxyacetate (12a).**

^{1}H NMR (400 MHz, $CDCl_3$)

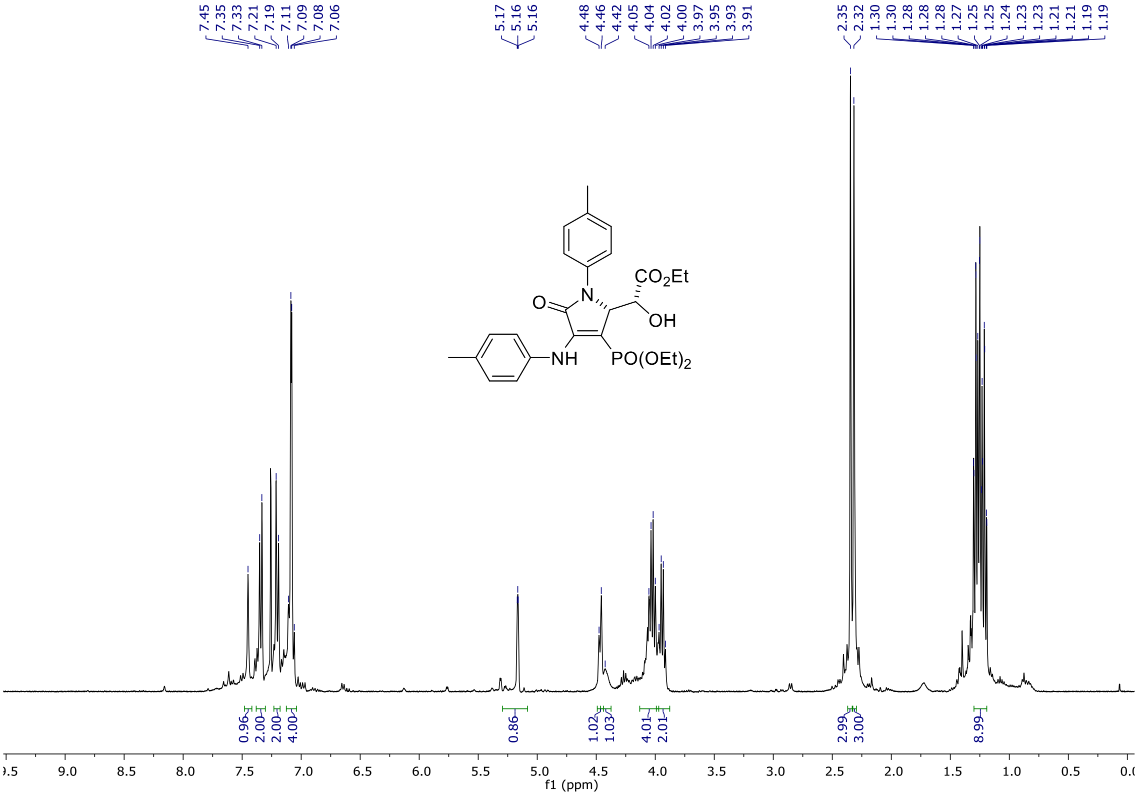

^{13}C NMR {^{1}H} (101 MHz, $CDCl_3$)

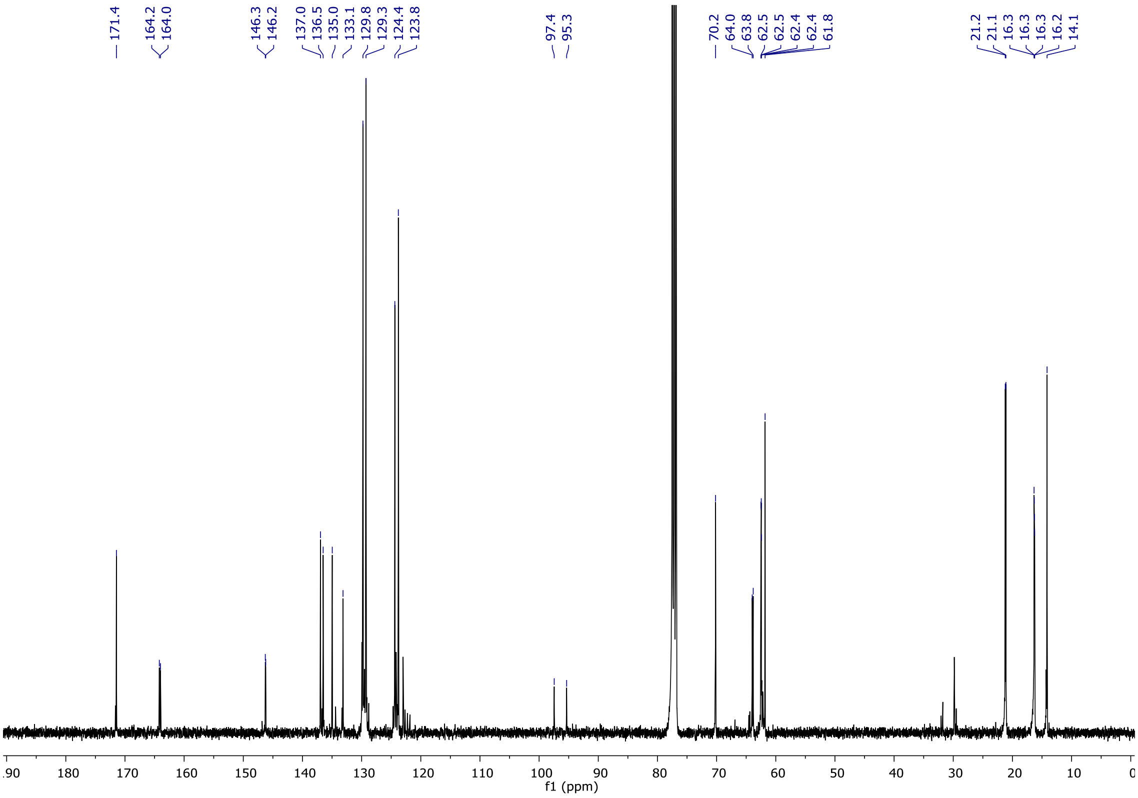

^{31}P-NMR (162 MHz, $CDCl_3$)

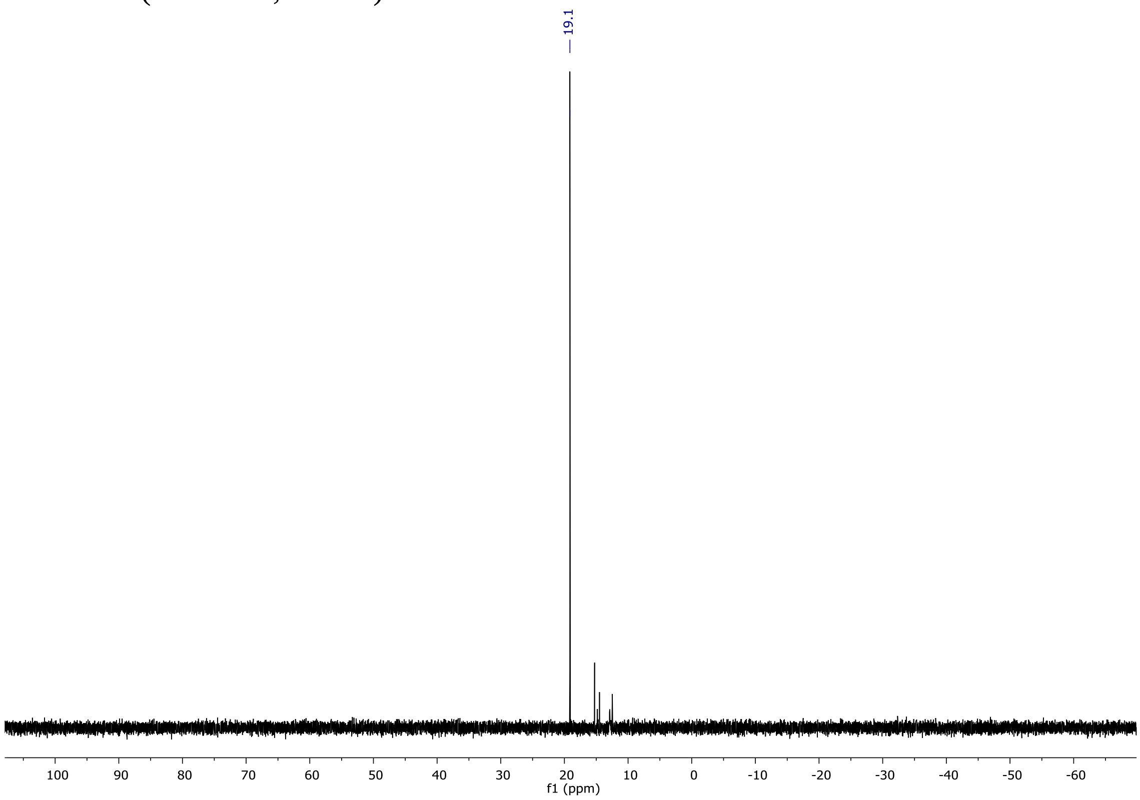

Ethyl (*R)-2-((*R**)-3-(diphenylphosphoryl)-5-oxo-1-(*p*-tolyl)-4-(*p*-tolylamino)-2,5-dihydro-1*H*-pyrrol-2-yl)-2-hydroxyacetate (12b).**

^{1}H NMR (400 MHz, $CDCl_3$)

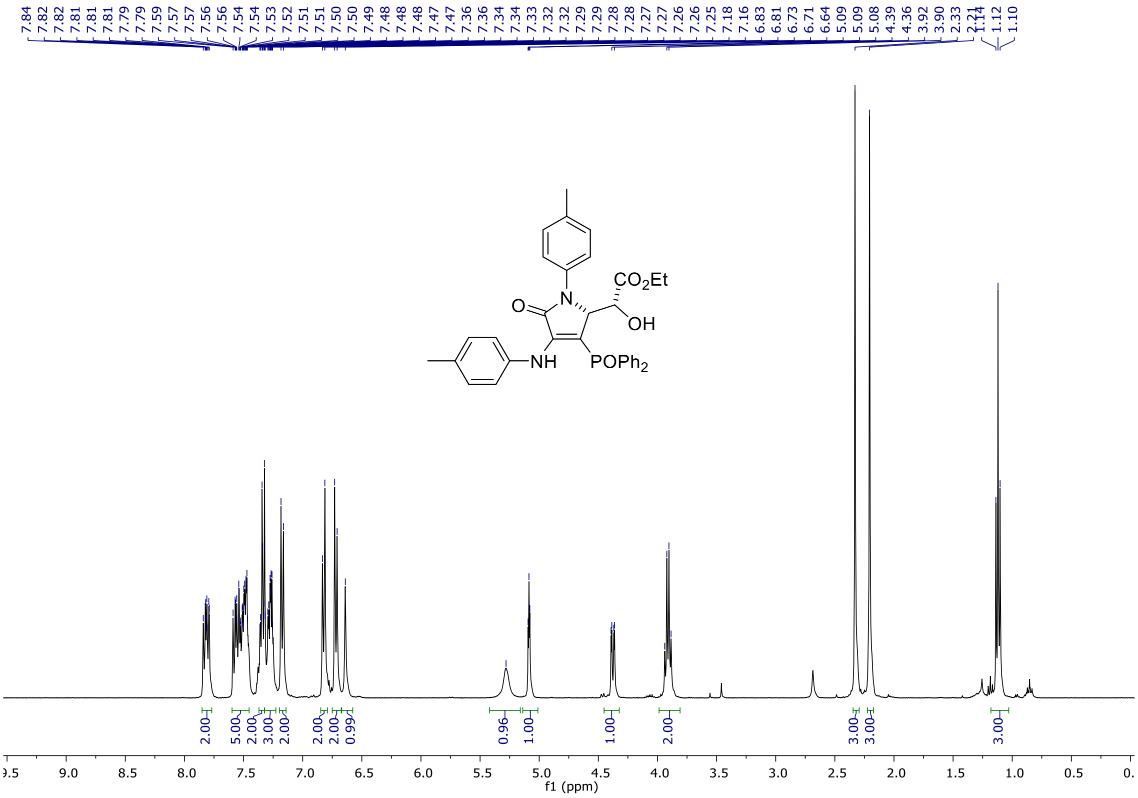

^{13}C NMR {^{1}H} (101 MHz, $CDCl_3$)

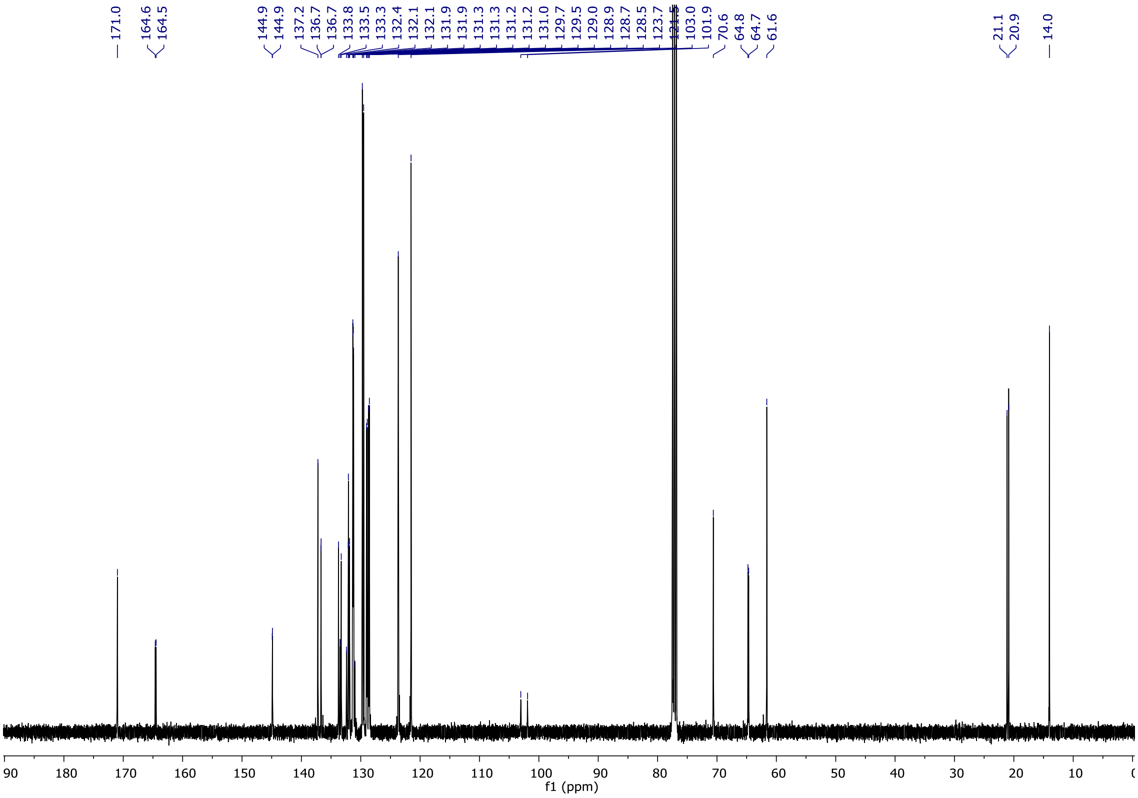

^{31}P NMR (121 MHz, $CDCl_3$)

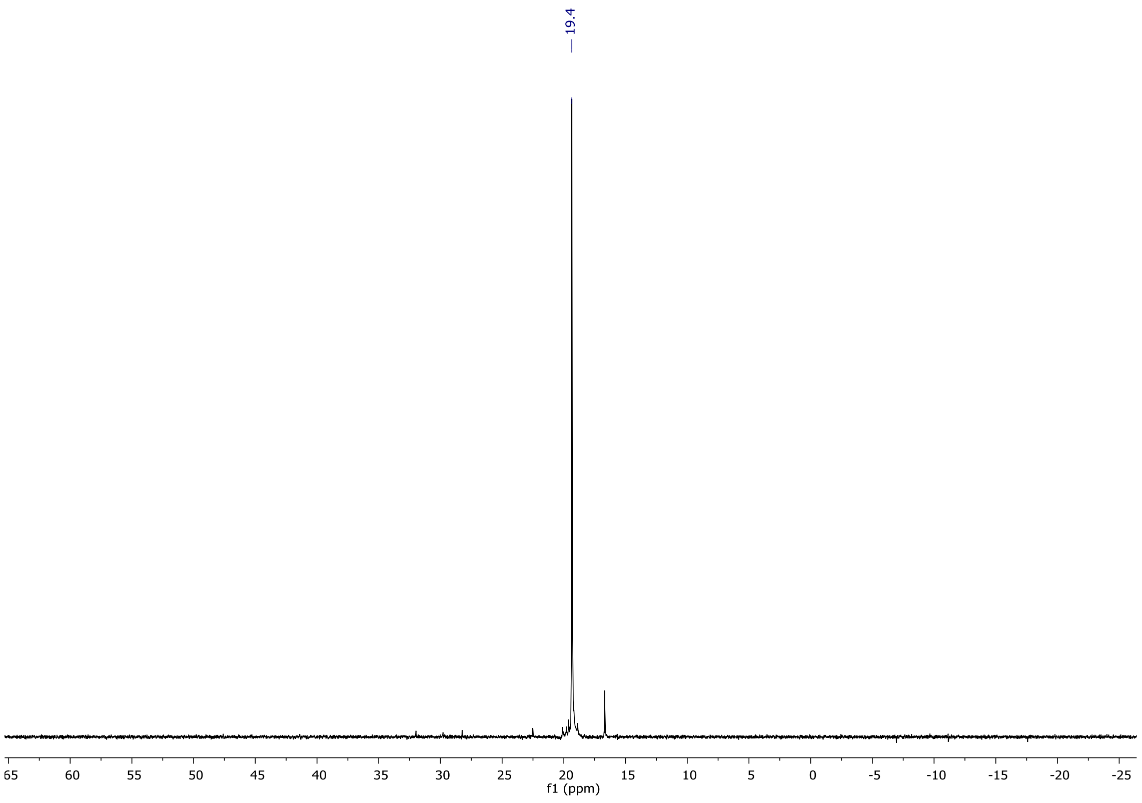

Crystal structure determination of compound 12b.

The structure of compound **12b** was determined on a crystal prepared from a CH_2Cl_2/ Et_2O solvent system by slow evaporation in a vial at room temperature. The X-ray data have been deposited at the Cambridge Crystallographic Data Centre (CCDC 2125880).

Intensity data were collected on an Agilent Technologies Super-Nova diffractometer, wich was equipped with monochromated Mo kα radiation (λ= 0.71073 Å) and Eos CCD detector. Measurement was carried out at 151.0(10) K with the help of an Oxford Cryostream 700 PLUS temperature device. Data frames were processed (united cell determination, analytical absorption correction with face indexing, intensity data integration and correction for Lorentz and polarization effects) using the Crysalis software package.[1] The structure was solved using SHELXT[2] and refined by full-matrix least-squares with SHELXL-97.[3] Final geometrical calculations were carried out with Mercury[4] and PLATON[5] as integrated in WinGX.[6]

Thermal ellipsoid plot/ORTEP for compound 12b. Contour probability level: 50%

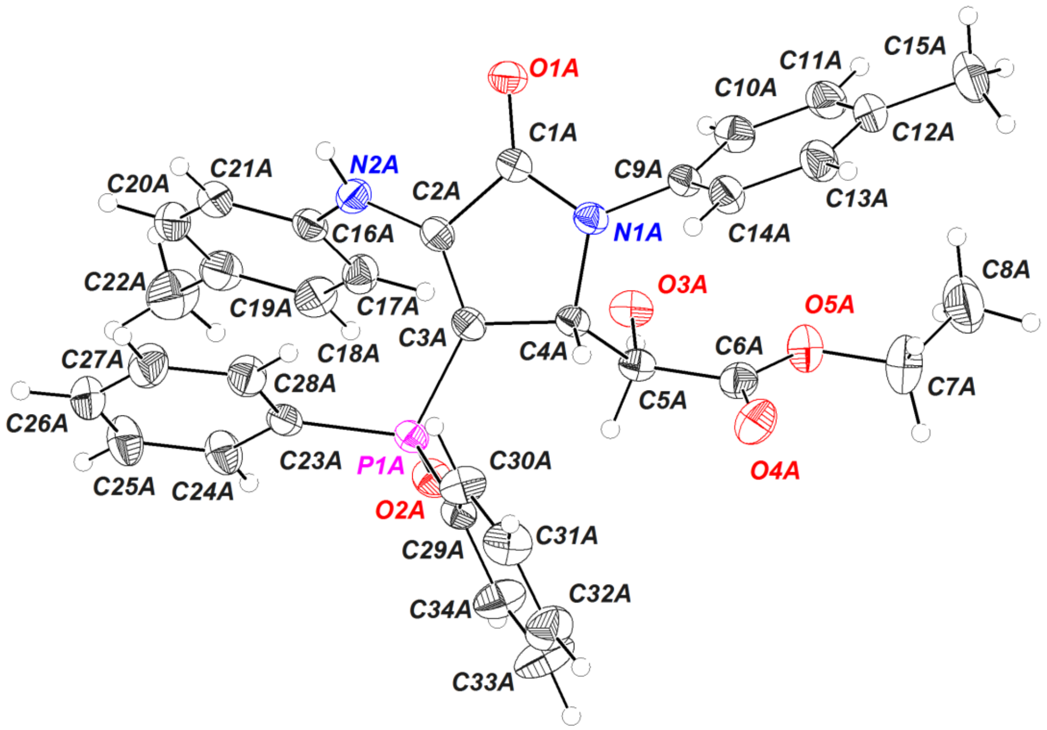

Table S1. Crystal data and structure refinement for 12b.

Identification code	a20210323_AL231

Empirical formula	$C_{34}H_{33}N_2O_5P$
Formula weight	580.59
Temperature/K	150.0(10)
Crystal system	triclinic
Space group	P-1
a/Å	12.64056(19)
b/Å	14.4828(2)
c/Å	18.2237(3)
α/°	71.6731(14)
β/°	88.2210(13)
γ/°	78.0417(12)
Volume/Å^3	3096.20(8)
Z	4
ρ_{calc}g/cm^3	1.246
μ/mm^{-1}	1.141
F(000)	1224.0
Crystal size/mm^3	0.203 × 0.101 × 0.082
Radiation	Mo Kα (λ = 0.71073)
2Θ range for data collection/°	6.574 to 137.998
Index ranges	$-15 \le h \le 15$, $-17 \le k \le 17$, $-22 \le l \le 21$
Reflections collected	47925
Independent reflections	11513 [R_{int} = 0.0405, R_{sigma} = 0.0308]
Data/restraints/parameters	11513/0/765
Goodness-of-fit on F^2	1.029
Final R indexes [I>=2σ (I)]	R_1 = 0.0386, wR_2 = 0.0994
Final R indexes [all data]	R_1 = 0.0482, wR_2 = 0.1061
Largest diff. peak/hole / e Å^{-3}	0.45/-0.34
Flack parameter	0.14(6)
Bijvoet Pairs Coverage	99%
Hooft y	0.14(4)
P2(false)	$\le 10^{-106}$

[1] CrysAlisPro, Agilent Technologies, Version 1.171.37.31 (reléase 14-01-2014 CryAlis171.NET) (compiled Jan 14 2014, 18:38:05).
[2] G.M. Sheldrick, *Acta Cryst*. **2015**, *A71*, 3.
[3] G.M. Sheldrick, *Acta Cryst.* **2008**, *A64*, 112; G.M. Sheldrick, *Acta Cryst.* **2015**, *C71*, 3.
[4] C. F. Macrae, *J. Appl. Cryst.* **2008**, *41*, 466.
[5] A. L. Spek (2010) PLATON, A Multipurpose Crystallographic Tool, Utrecht University, Utrecht, The Netherlands; A. L. Spek, *J. Appl. Cryst.* **2003**, *36*, 7.
[6] L. J. Farrugia, *J. Appl. Cryst.* **1999**, *32*, 837.

3. Characterization by NMR Spectroscopy and Crystal Structure Determination of product 13.

(*Z*)-4-(Diphenylphosphoryl)-5-((4-nitrophenyl)imino)-1-(*p*-tolyl)-3-(*p*-tolylamino)-1,5-dihydro-2*H*-pyrrol-2-one (13).

^{1}H NMR (400 MHz, $CDCl_3$)

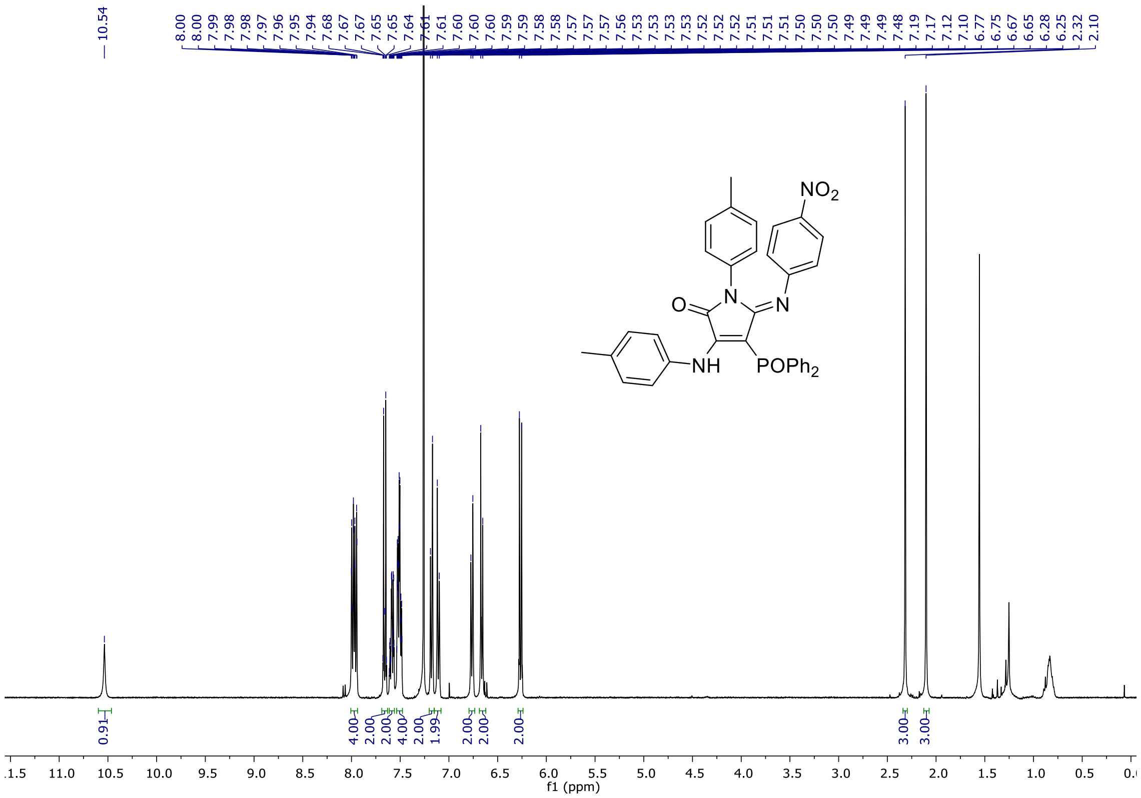

^{13}C NMR {^{1}H} (101 MHz, $CDCl_3$)

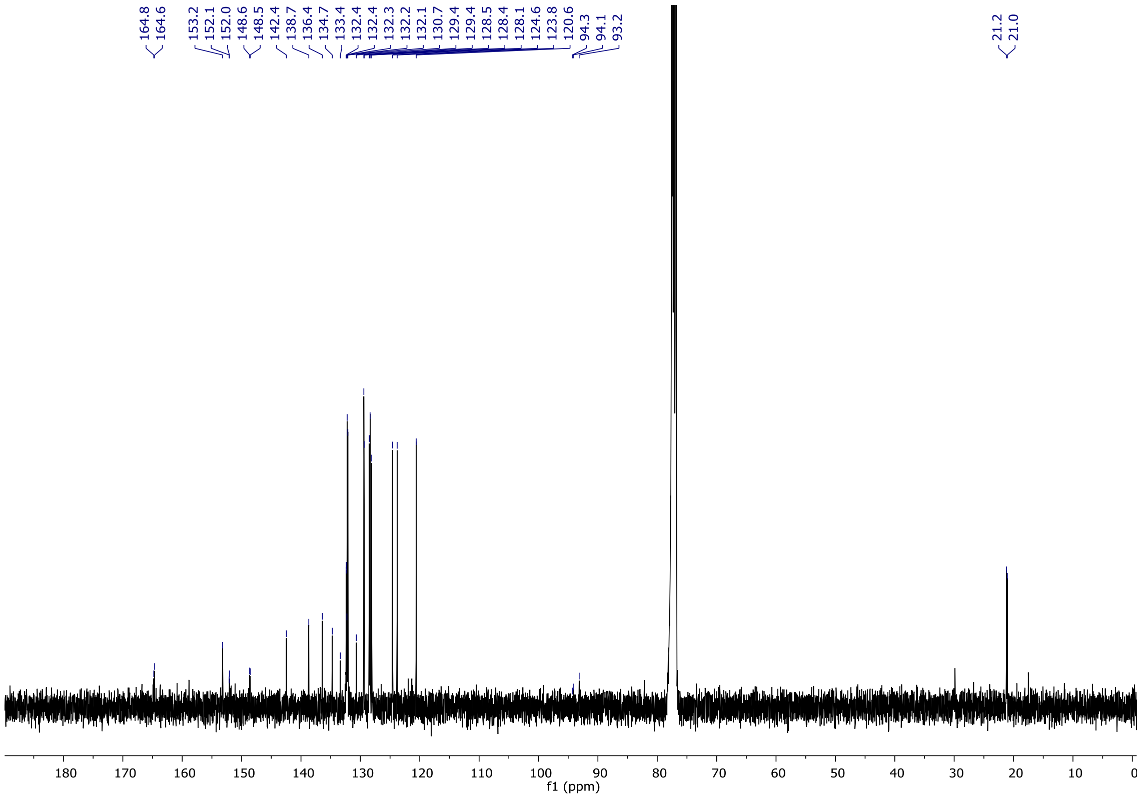

^{31}P-NMR (162 MHz, $CDCl_3$)

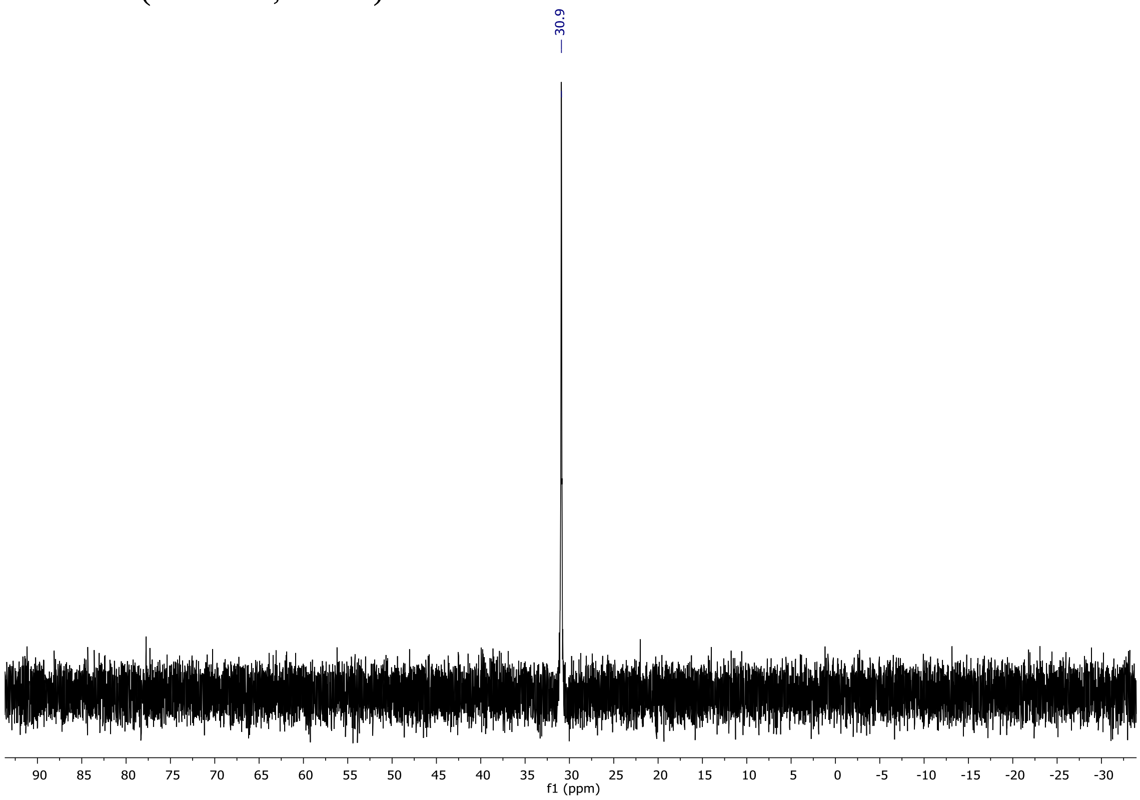

Crystal structure determination of compound 13.

The structure of compound **13** was determined on a crystal prepared from a CH_2Cl_2/ hexane solvent system by slow evaporation in a vial at room temperature. The X-ray data have been deposited at the Cambridge Crystallographic Data Centre (CCDC 2125933).

Intensity data were collected on an Agilent Technologies Super-Nova diffractometer, wich was equipped with monochromated Cu kα radiation (λ= 1.54184 Å) and Atlas CCD detector. Measurement was carried out at 151.0(5) K with the help of an Oxford Cryostream 700 PLUS temperature device. Data frames were processed (united cell determination, analytical absorption correction with face indexing, intensity data integration and correction for Lorentz and polarization effects) using the Crysalis software package.[¡Error! Marcador no definido.] The structure was solved using SHELXT[¡Error! Marcador no definido.] and refined by full-matrix least-squares with SHELXL-97.[¡Error! Marcador no definido.] Final geometrical calculations were carried out with Mercury[¡Error! Marcador no definido.] and PLATON[¡Error! Marcador no definido.] as integrated in WinGX.[¡Error! Marcador no definido.]

Thermal ellipsoid plot/ORTEP for compound 13. Contour probability level: 50%

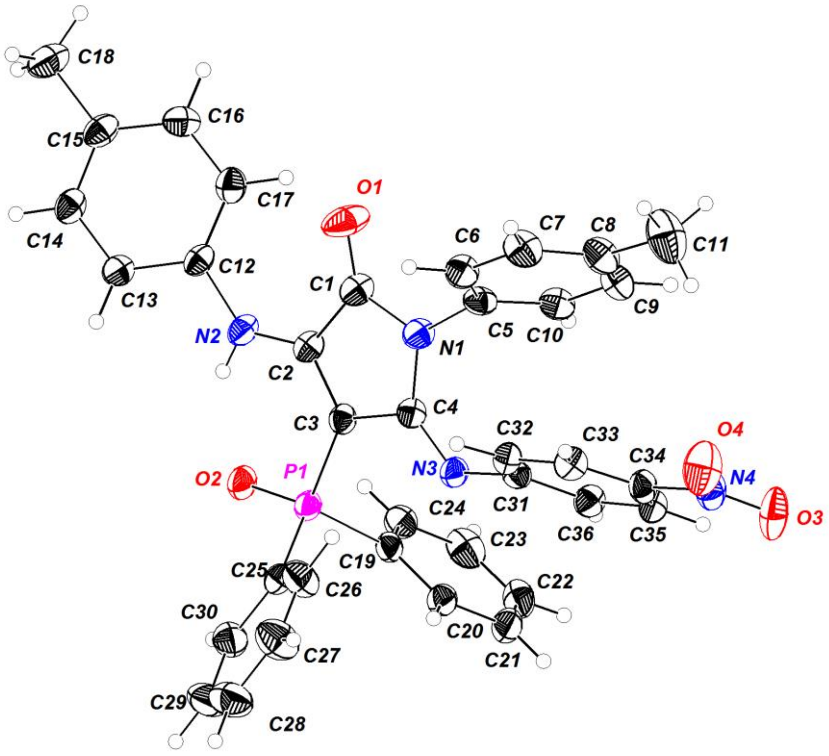

Table S2. Crystal data and structure refinement for 13.

Identification code	a20210337_AL426B
Empirical formula	$C_{36}H_{29}N_4O_4P$
Formula weight	612.60
Temperature/K	150.0(5)
Crystal system	triclinic
Space group	P-1
a/Å	8.0982(4)
b/Å	9.7541(5)
c/Å	19.6381(9)
α/°	84.400(4)
β/°	87.924(4)
γ/°	77.994(4)
Volume/Å^3	1509.85(13)
Z	2
ρ_{calc}g/cm^3	1.347
μ/mm^{-1}	1.198
F(000)	640.0
Crystal size/mm^3	0.256 × 0.095 × 0.062
Radiation	CuKα (λ = 1.54184)
2Θ range for data collection/°	9.05 to 137.95
Index ranges	-9 ≤ h ≤ 9, -11 ≤ k ≤ 11, -23 ≤ l ≤ 23
Reflections collected	21424
Independent reflections	5600 [R_{int} = 0.0766, R_{sigma} = 0.0666]
Data/restraints/parameters	5600/0/408
Goodness-of-fit on F^2	1.018
Final R indexes [I>=2σ (I)]	R1 = 0.0485, wR2 = 0.1094
Final R indexes [all data]	R1 = 0.0753, wR2 = 0.1247
Largest diff. peak/hole / e Å^{-3}	0.37/-0.35

4. Characterization by NMR Spectroscopy of products 18 and 19.

4-Methyl-5-phenyl-1-(*p*-tolyl)-3-(*p*-tolylamino)-1,5-dihydro-2*H*-pyrrol-2-one (18).

^{1}H NMR (400 MHz, $CDCl_3$)

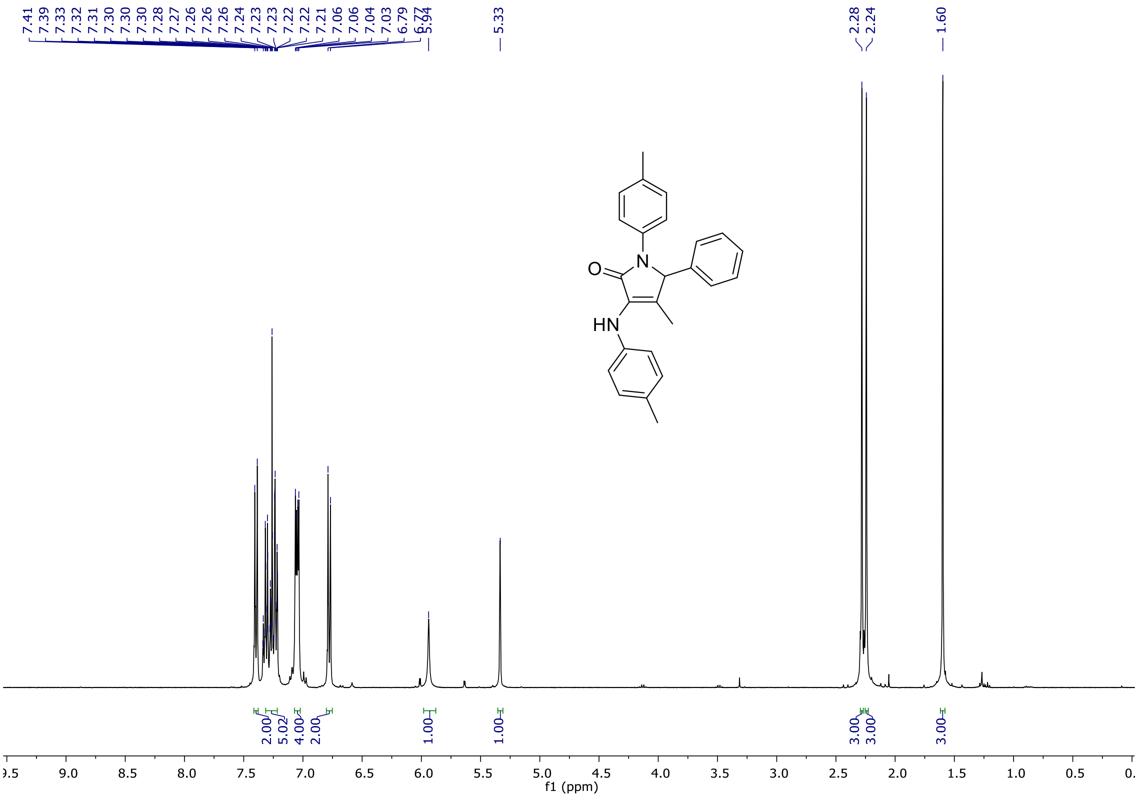

^{13}C NMR {^{1}H} (101 MHz, $CDCl_3$)

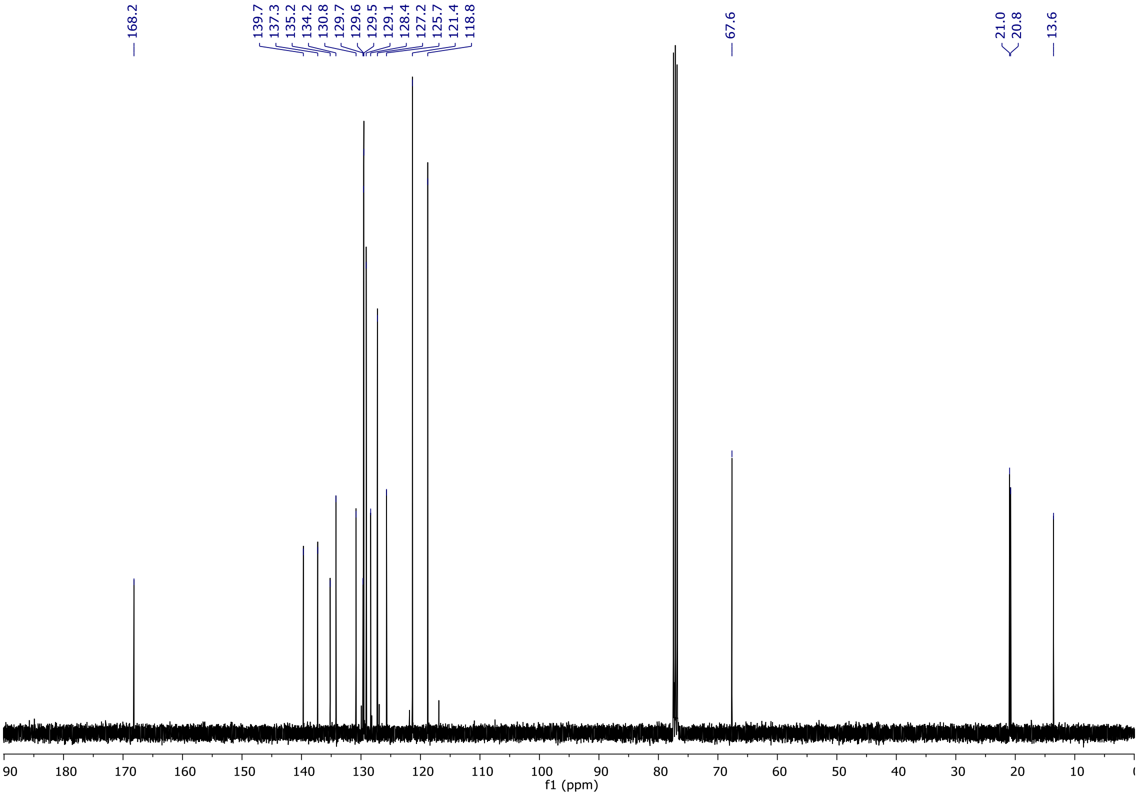

2D-COSY NMR {^{1}H – ^{1}H} (400 MHz, $CDCl_3$)

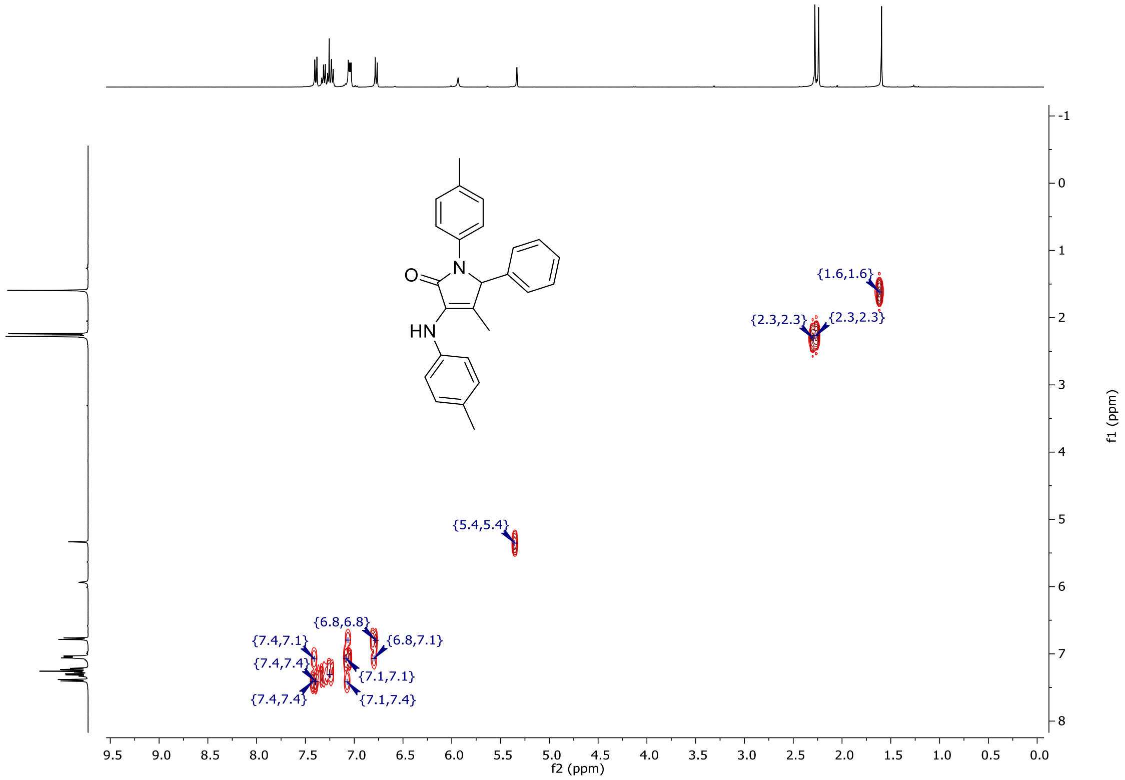

2D-HSQC NMR {^{1}H – ^{13}C} (^{1}H: 400 MHz, ^{13}C: 101 MHz, $CDCl_3$)

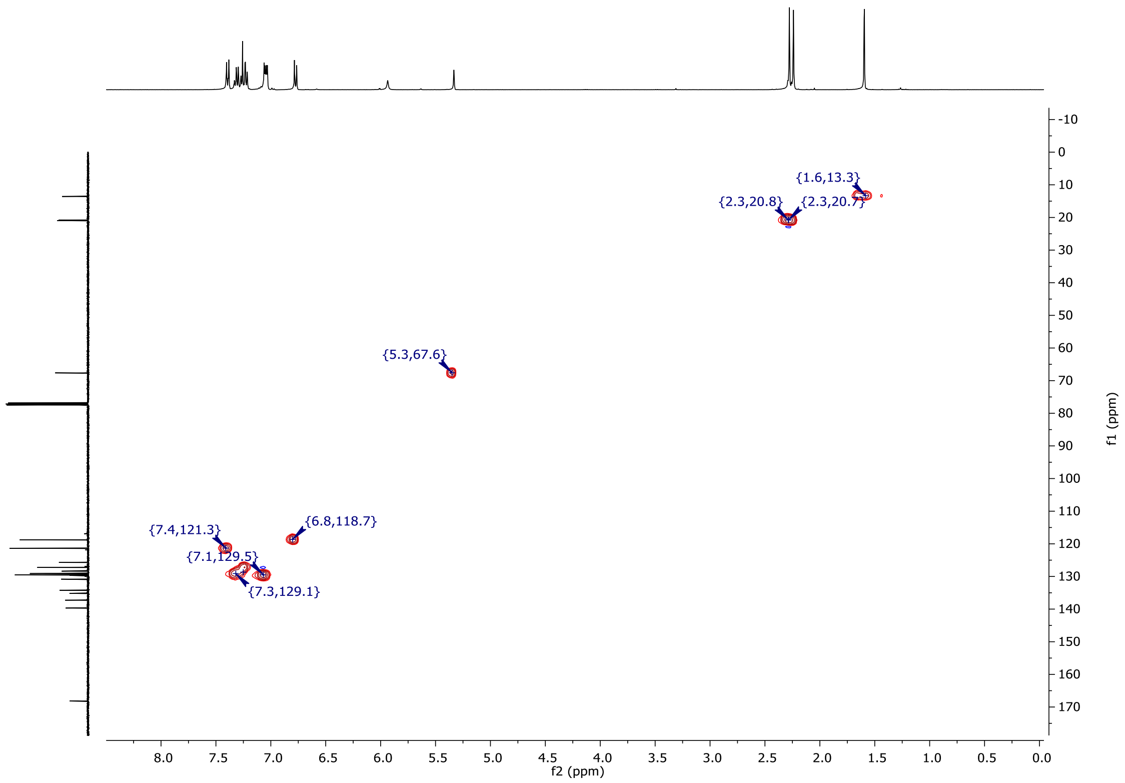

2D-HMBC NMR {^{1}H – ^{13}C} (^{1}H: 400 MHz, ^{13}C: 101 MHz, $CDCl_3$)

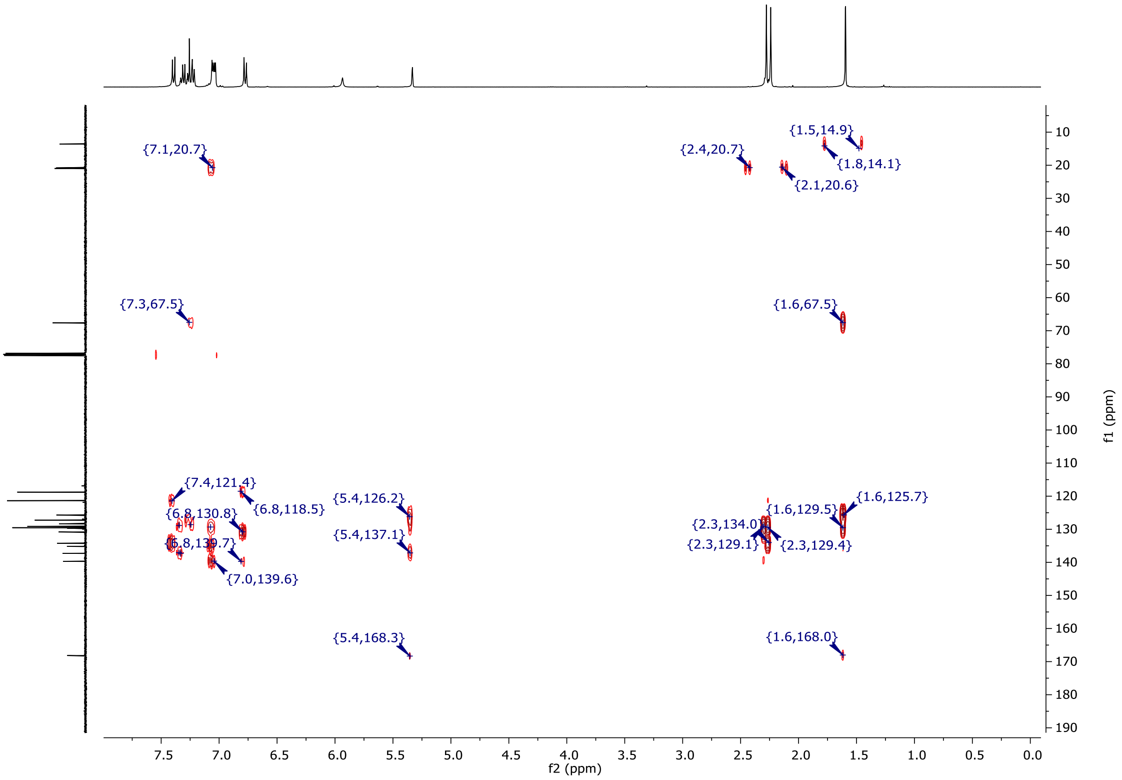

3-Hydroxy-4-methyl-5-phenyl-1-(*p*-tolyl)-1,5-dihydro-2*H*-pyrrol-2-one (19).

^{1}H NMR (400 MHz, $CDCl_3$)

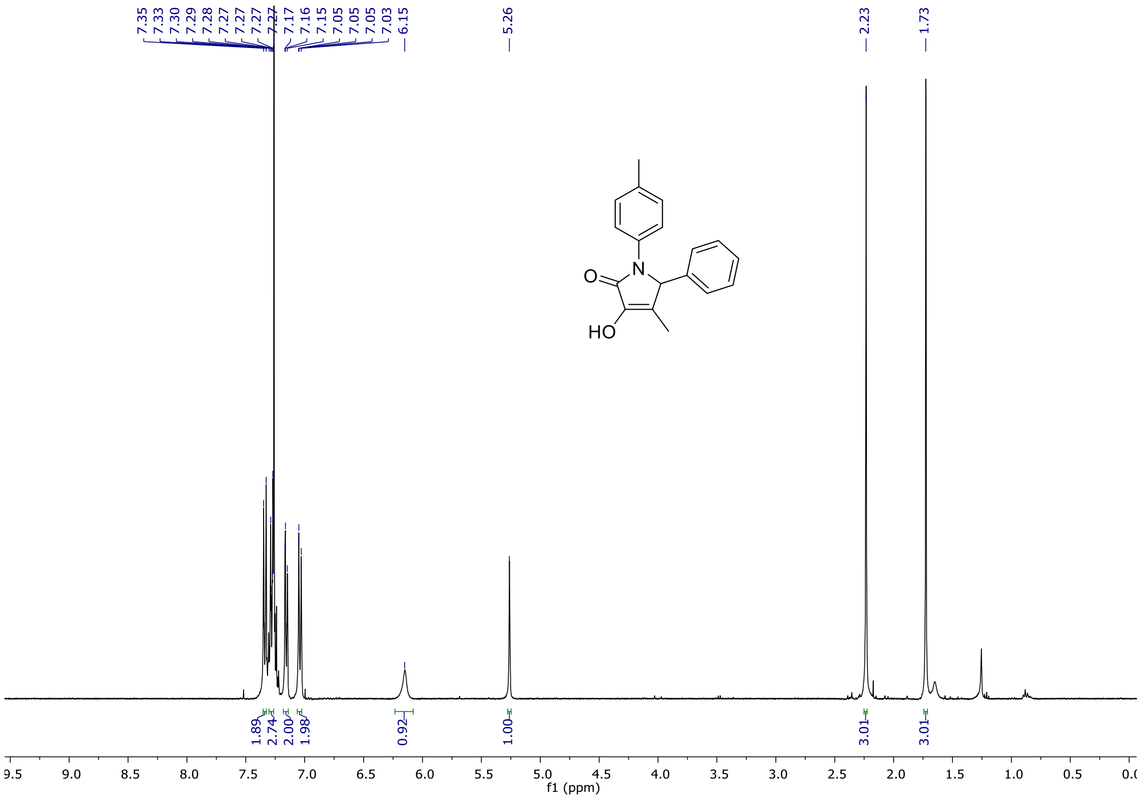

^{13}C NMR {^{1}H} (101 MHz, $CDCl_3$)

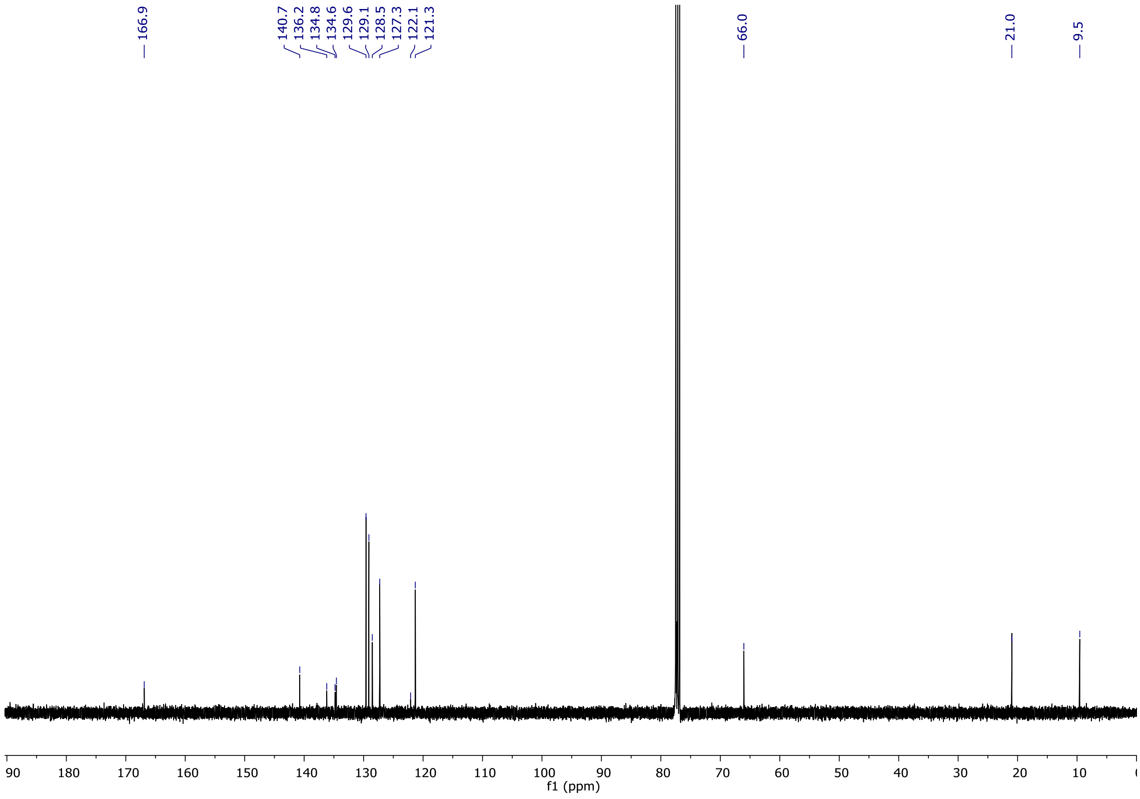

5. Characterization by NMR Spectroscopy of product 20.

Ethyl 3-((dimethylamino)methyl)-5-oxo-1-(*p*-tolyl)-4-(*p*-tolylamino)-2,5-dihydro-1*H*-pyrrole-2-carboxylate (20).

^{1}H NMR (400 MHz, $CDCl_3$)

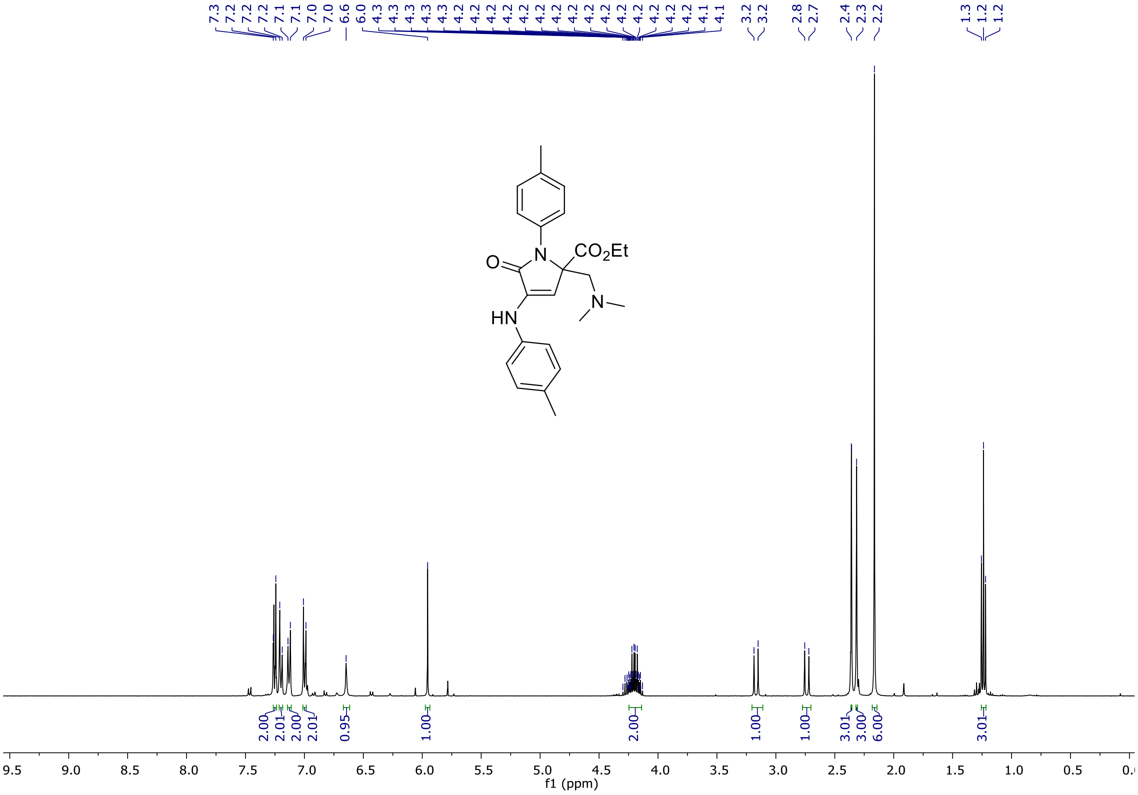

^{13}C NMR {^{1}H} (101 MHz, $CDCl_3$)

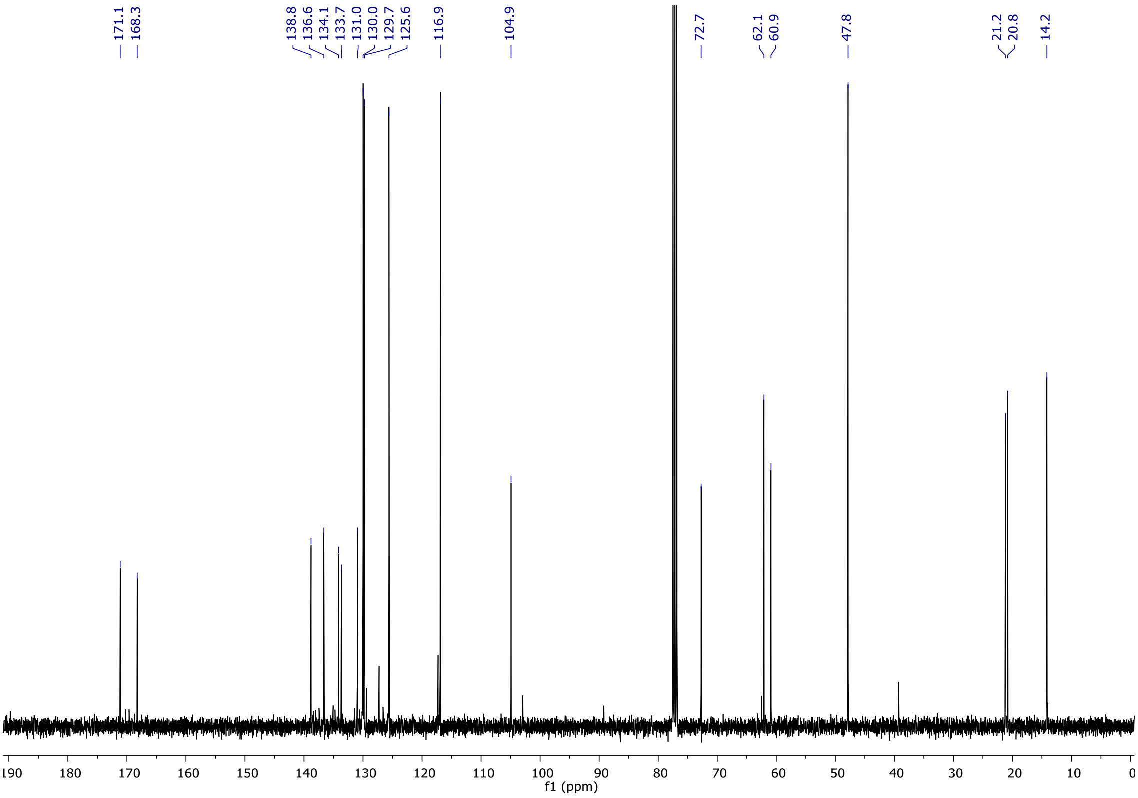

2D-COSY NMR {^{1}H – ^{1}H} (400 MHz, $CDCl_3$)

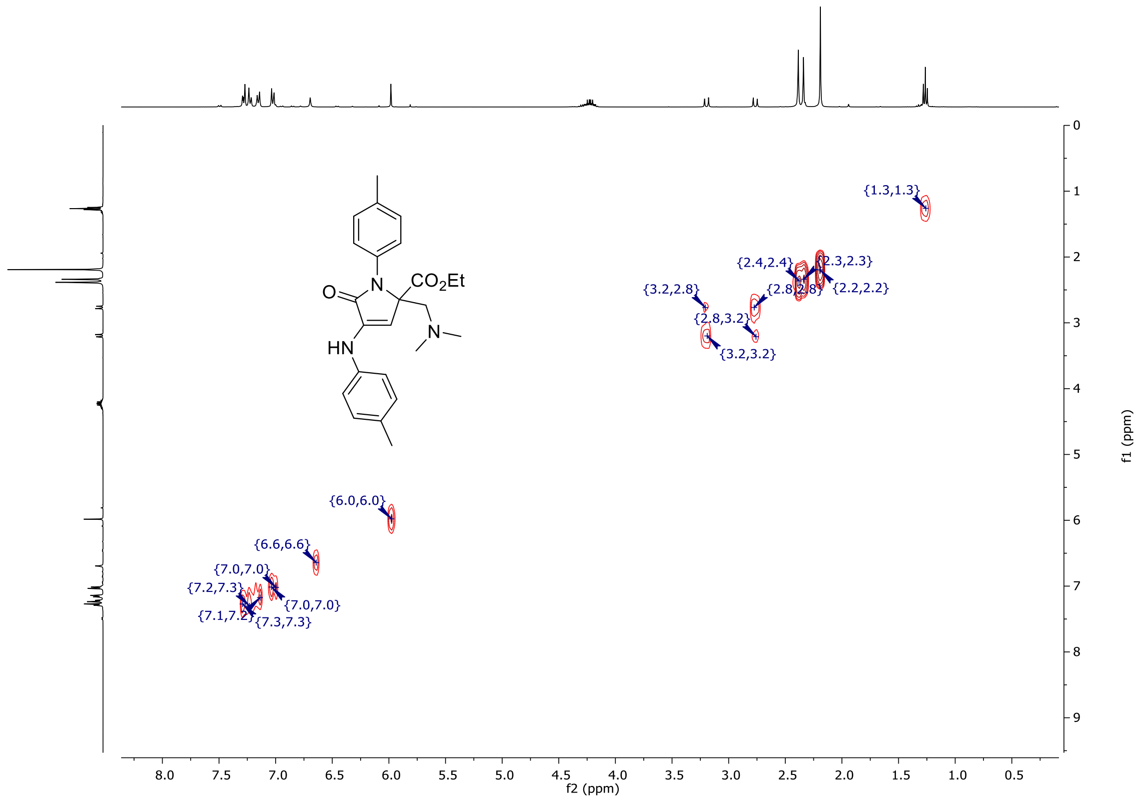

2D-HSQC NMR {^{1}H – ^{13}C} (^{1}H: 400 MHz, ^{13}C: 101 MHz, $CDCl_3$)

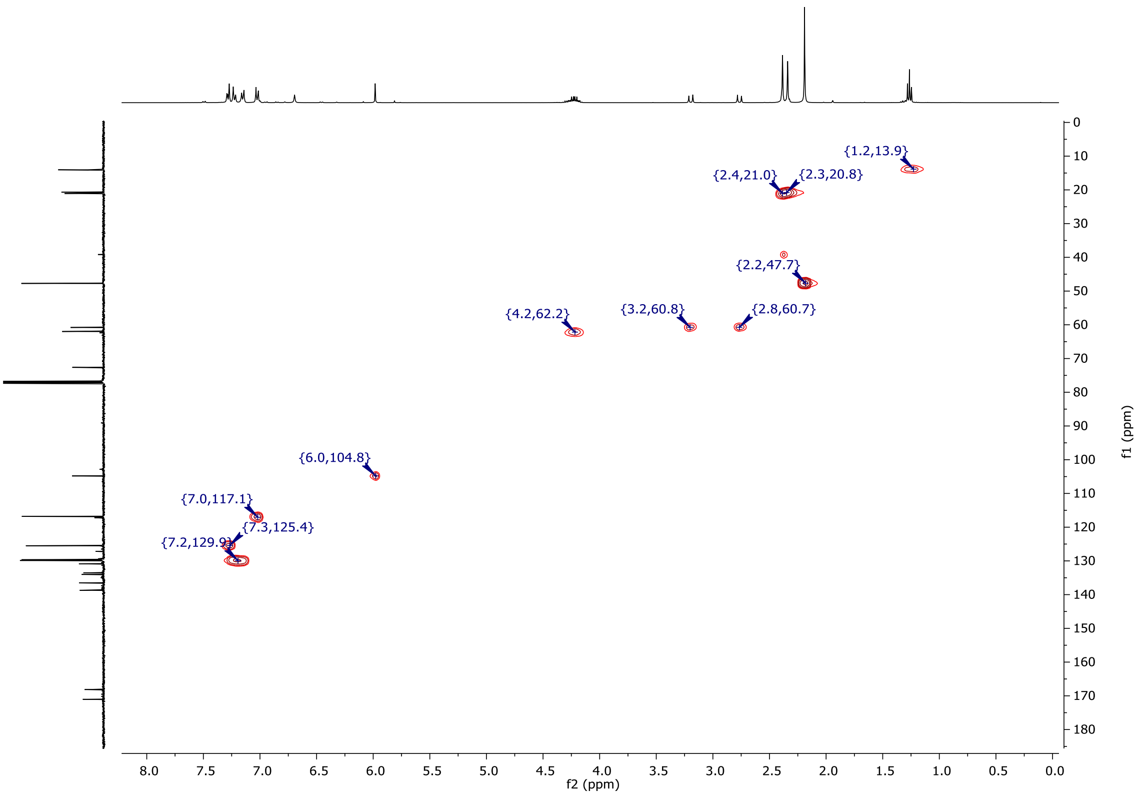

2D-HMBC NMR {^{1}H – ^{13}C} (^{1}H: 400 MHz, ^{13}C: 101 MHz, $CDCl_3$)

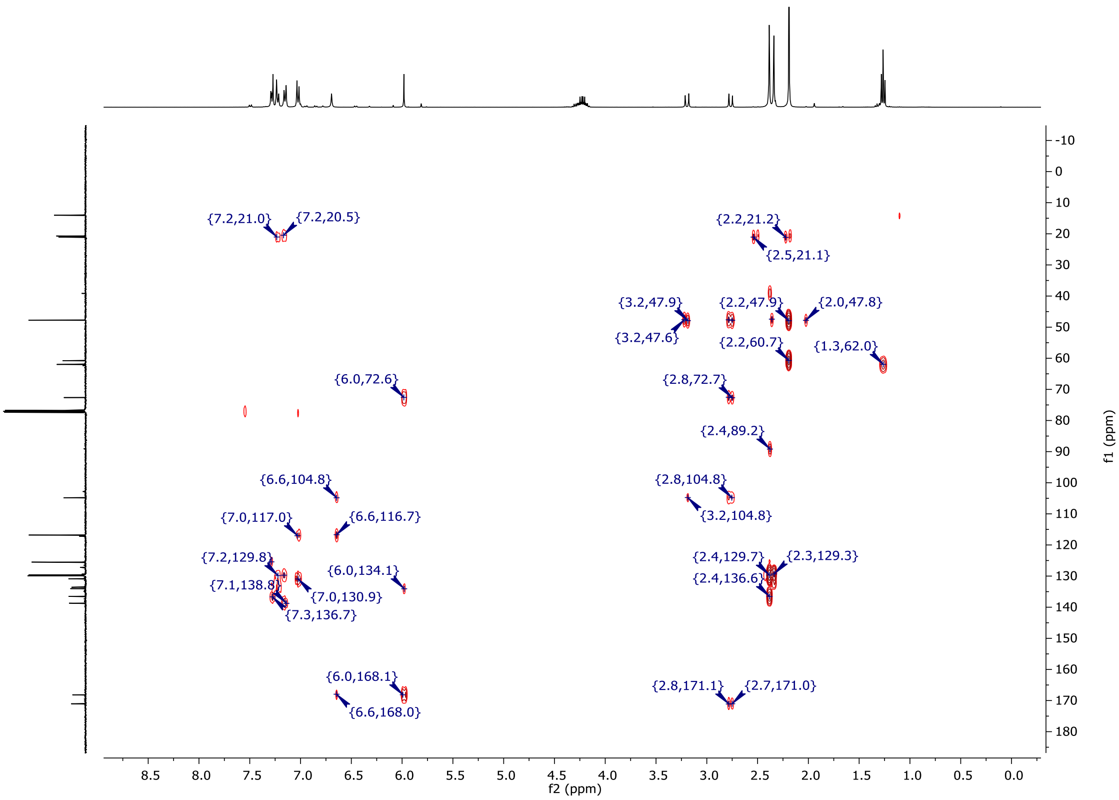

6. Characterization by NMR Spectroscopy of products 21 and 23.

Ethyl (2*S**,4S*)-5-oxo-1-(*p*-tolyl)-4-(*p*-tolylamino)pyrrolidine-2-carboxylate (21)

^{1}H NMR (400 MHz, $CDCl_3$)

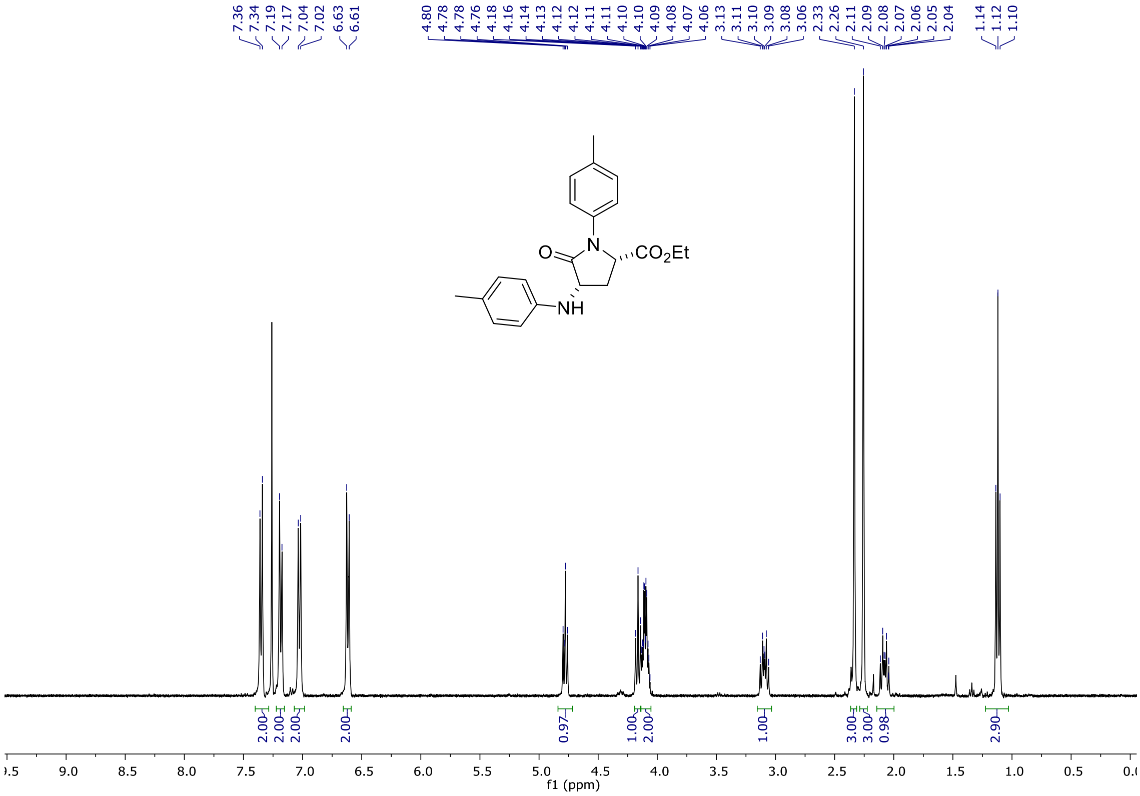

^{13}C NMR {^{1}H} (101 MHz, $CDCl_3$)

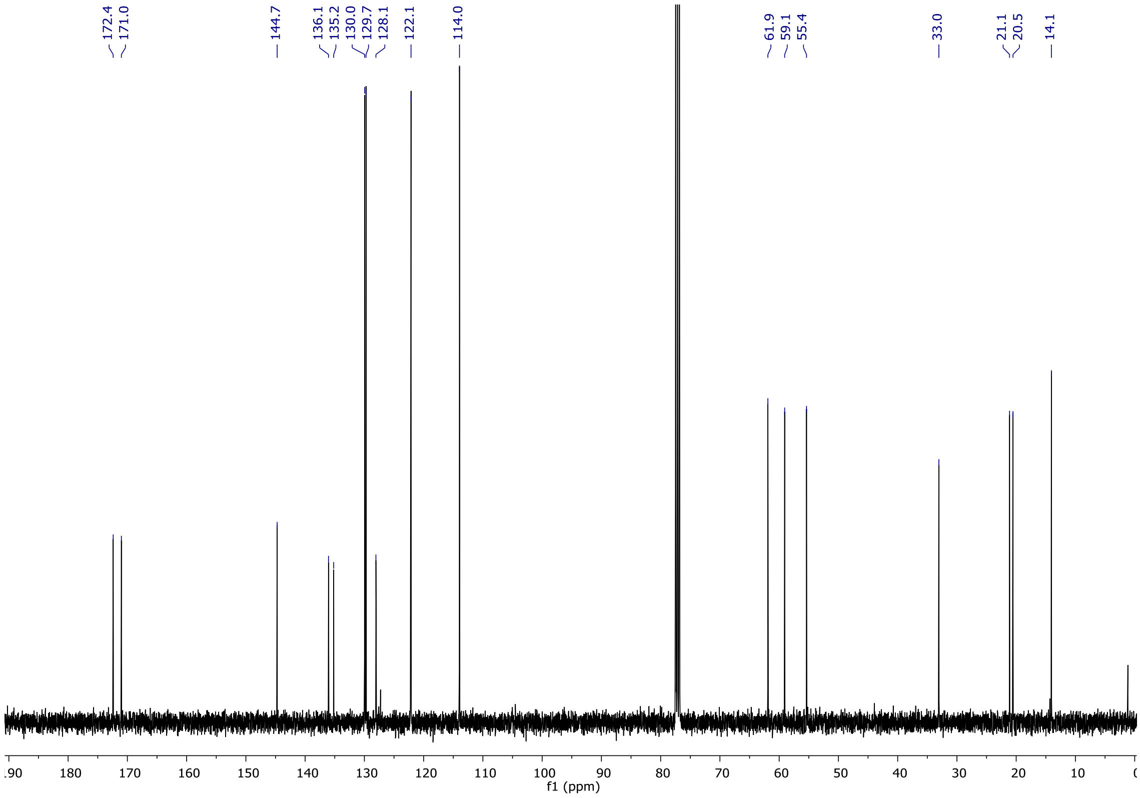

1D-NOESY {^{1}H – ^{1}H} (400 MHz, $CDCl_3$)

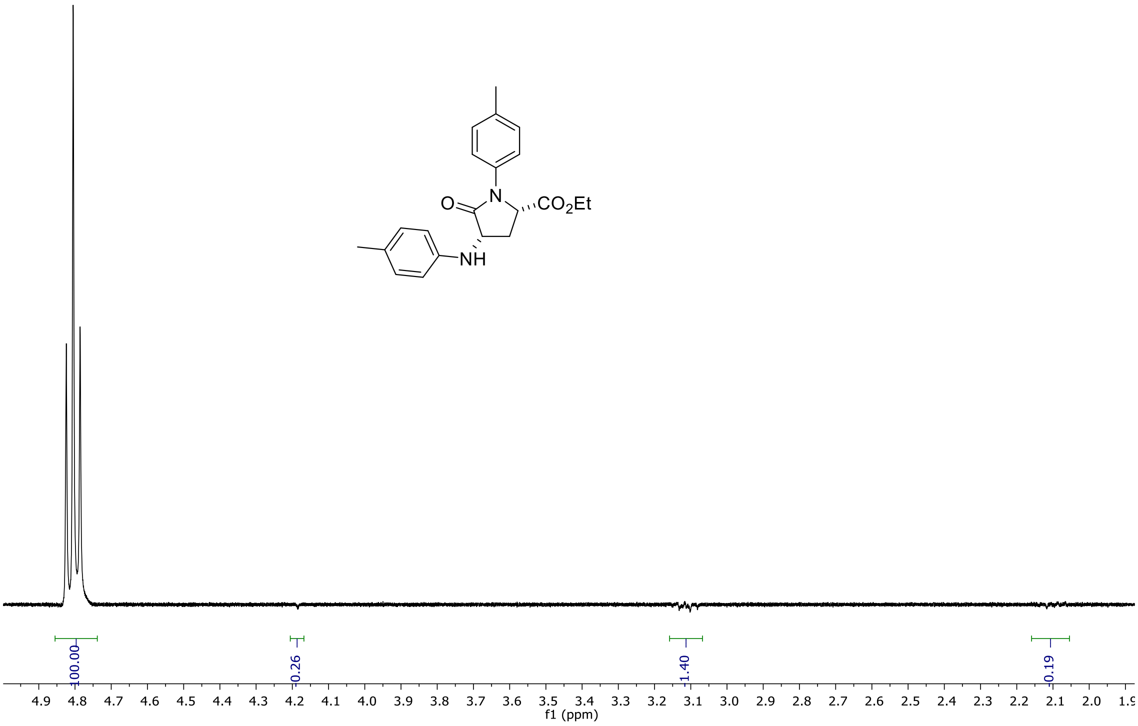

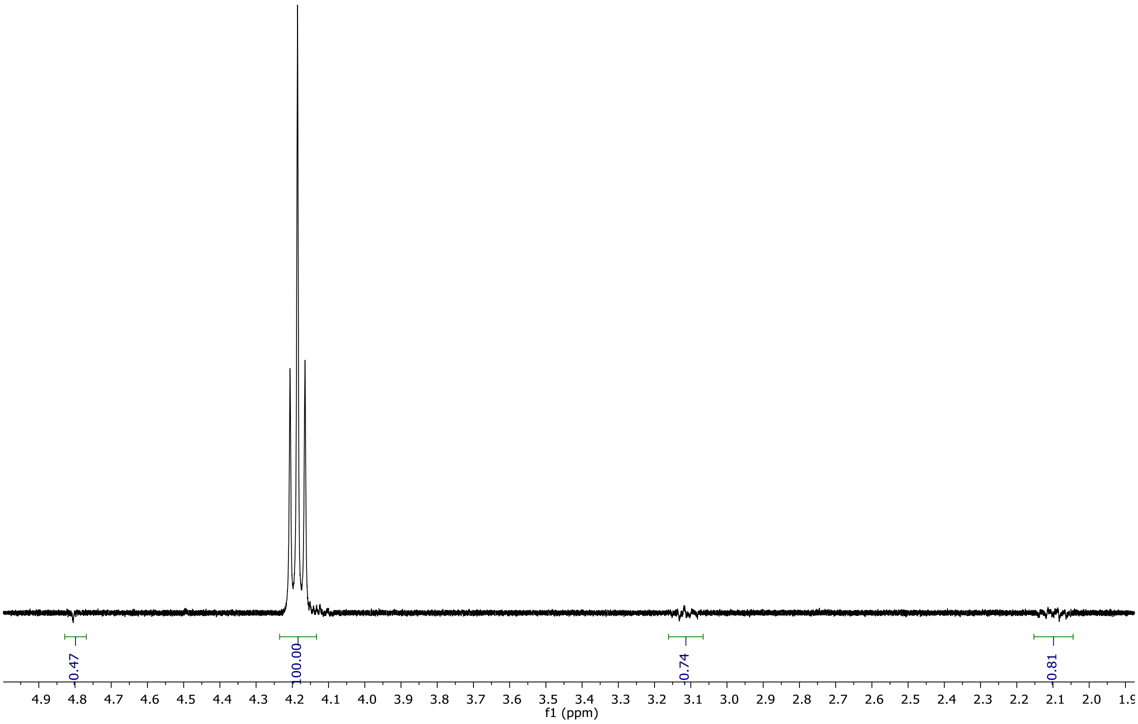

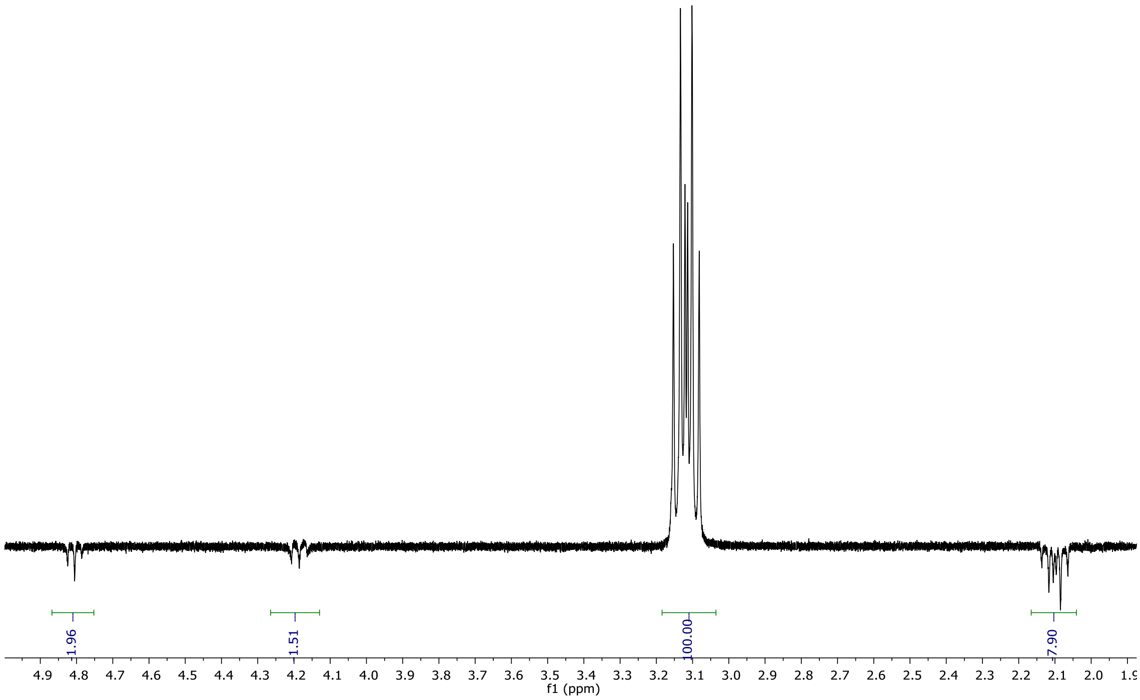
1.96
1.51
100.00
7.90
4.9 4.8 4.7 4.6 4.5 4.4 4.3 4.2 4.1 4.0 3.9 3.8 3.7 3.6 3.5 3.4 3.3 3.2 3.1 3.0 2.9 2.8 2.7 2.6 2.5 2.4 2.3 2.2 2.1 2.0
f1 (ppm)

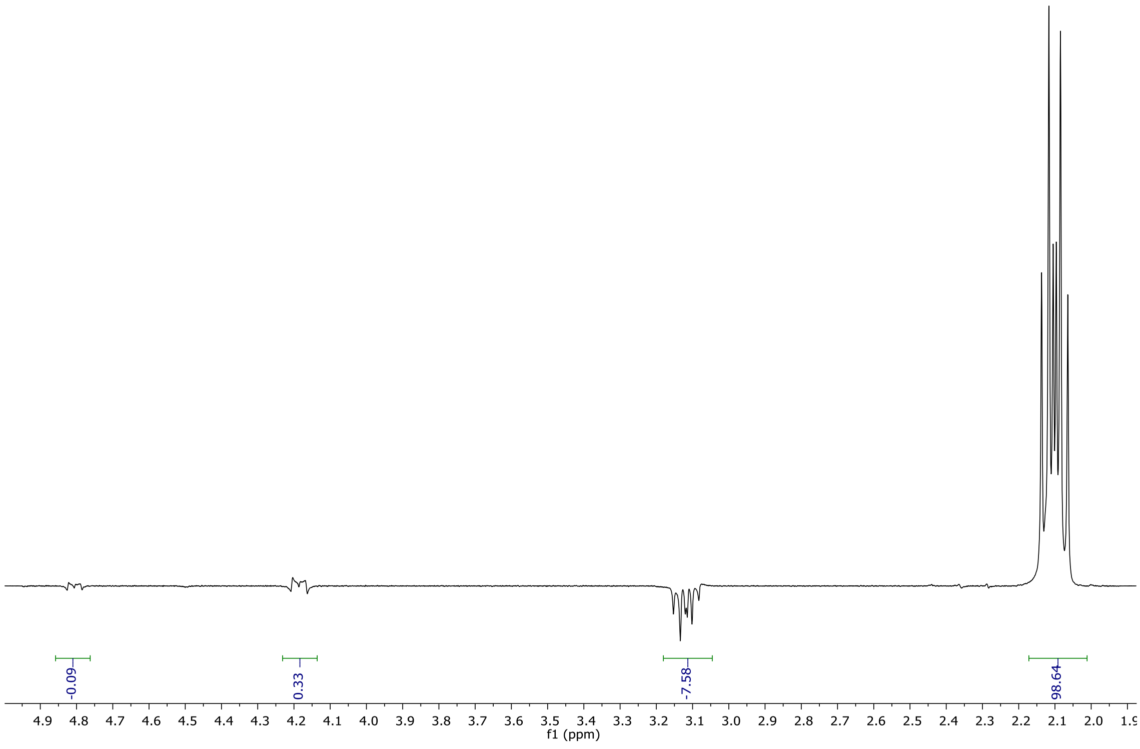
-0.09
0.33
-7.58
98.64
4.9 4.8 4.7 4.6 4.5 4.4 4.3 4.2 4.1 4.0 3.9 3.8 3.7 3.6 3.5 3.4 3.3 3.2 3.1 3.0 2.9 2.8 2.7 2.6 2.5 2.4 2.3 2.2 2.1 2.0
f1 (ppm)

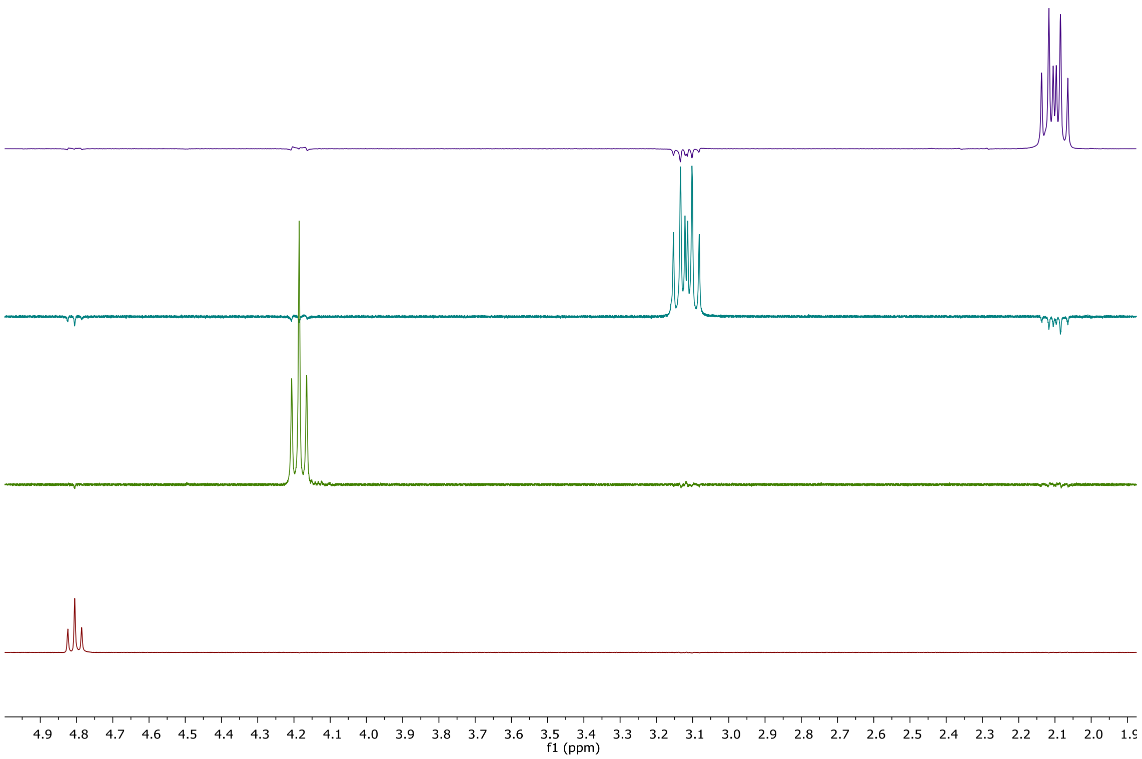
4.9 4.8 4.7 4.6 4.5 4.4 4.3 4.2 4.1 4.0 3.9 3.8 3.7 3.6 3.5 3.4 3.3 3.2 3.1 3.0 2.9 2.8 2.7 2.6 2.5 2.4 2.3 2.2 2.1 2.0
f1 (ppm)

5-Ethyl 2-methyl (4*S,5*S**)-4-(4-nitrophenyl)-7-oxo-1,6-di-*p*-tolyl-4,5,6,7-tetrahydro-1*H*-pyrrolo[3,4-*b*]pyridine-2,5-dicarboxylate (23)**

^{1}H NMR (400 MHz, $CDCl_3$)

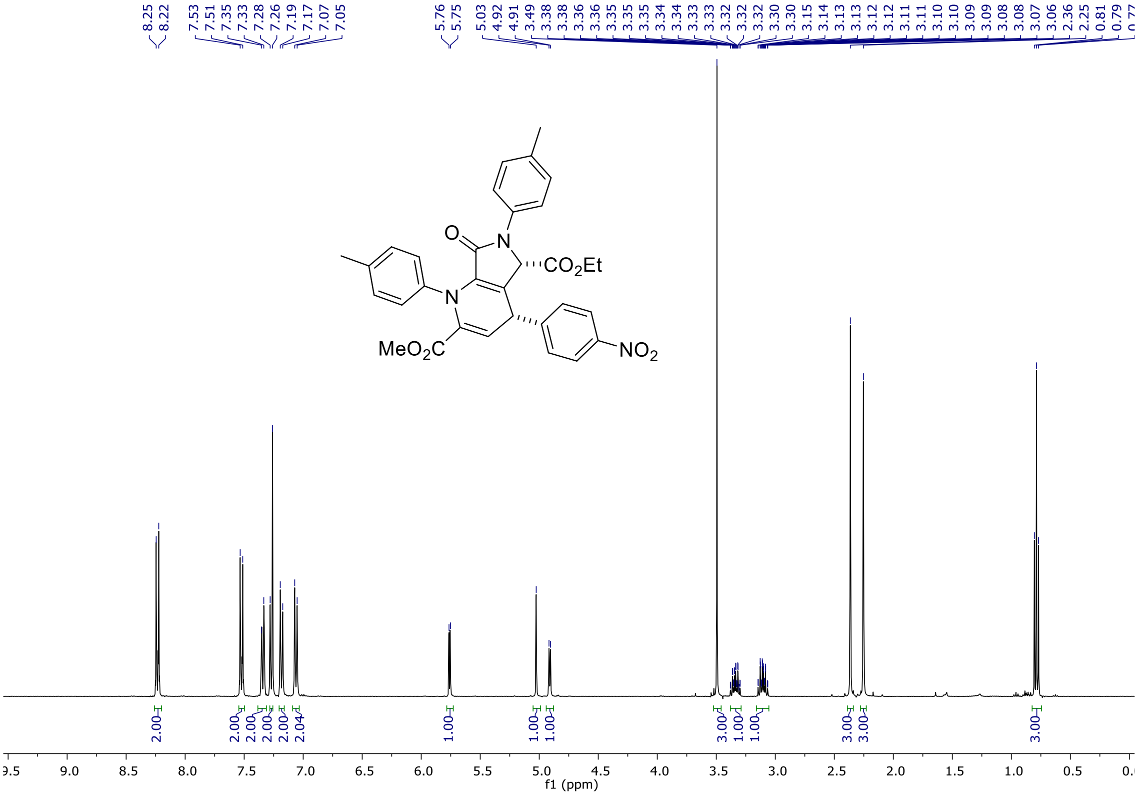

^{13}C NMR {^{1}H} (101 MHz, $CDCl_3$)

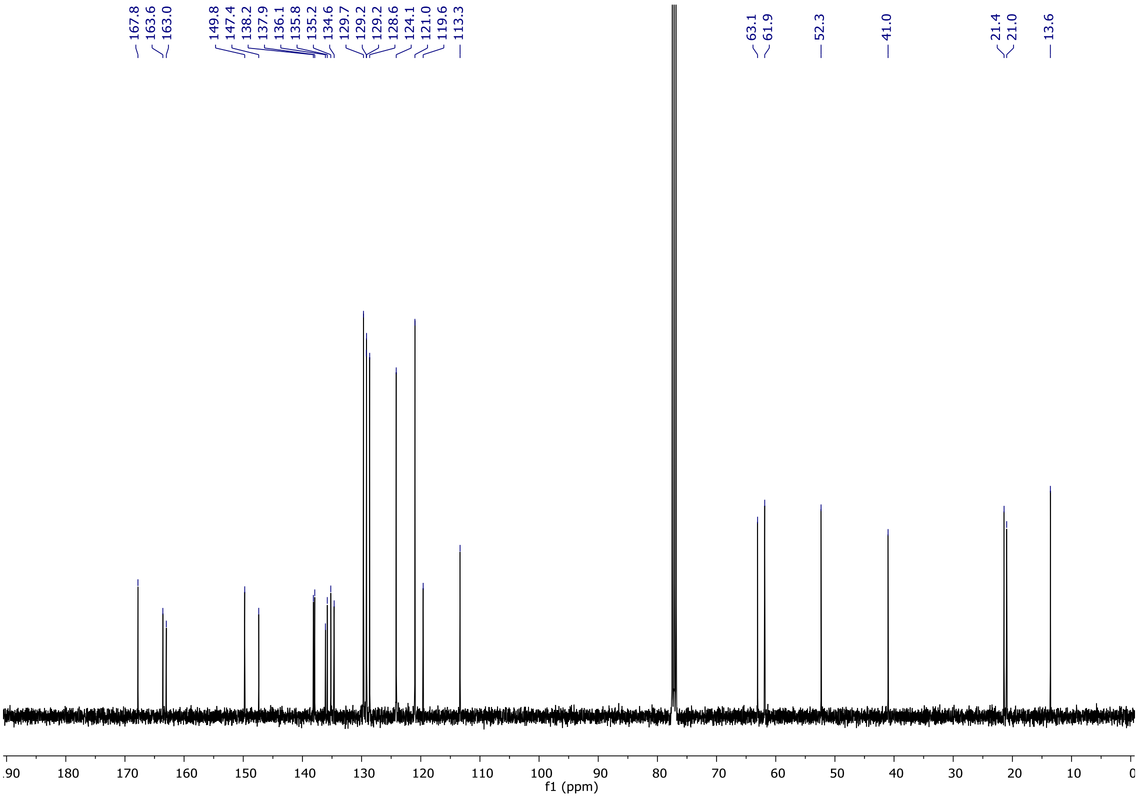

1D-NOESY {^{1}H – ^{1}H} (400 MHz, $CDCl_3$)

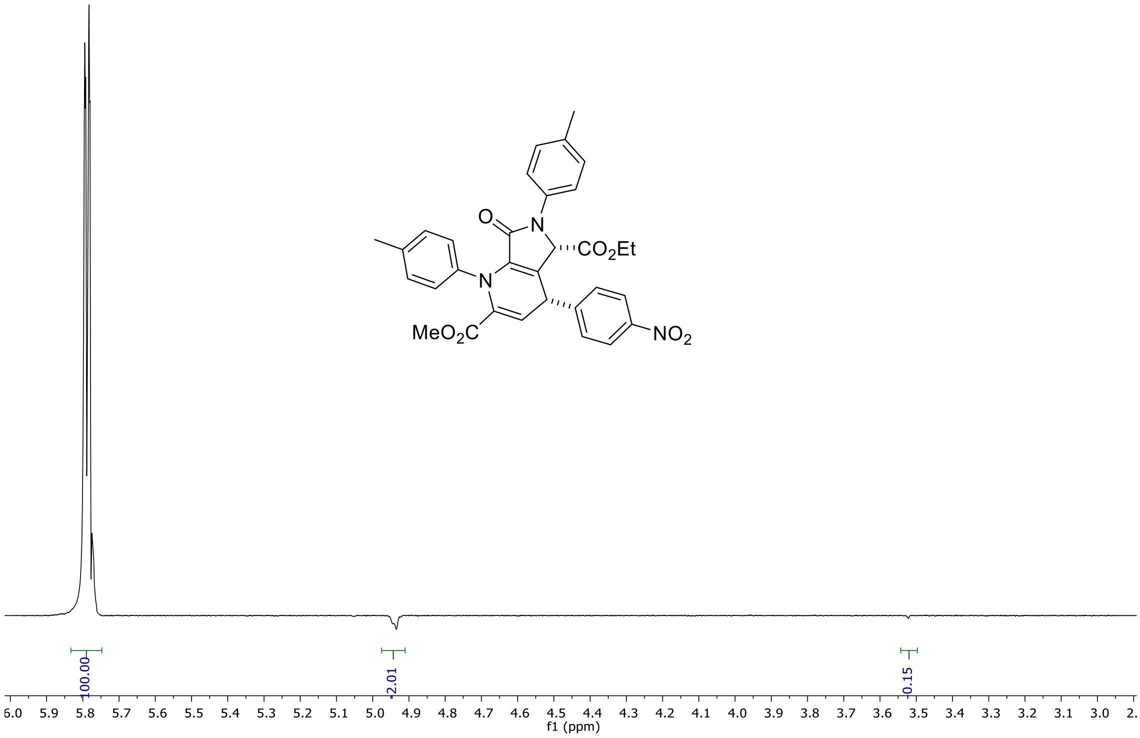

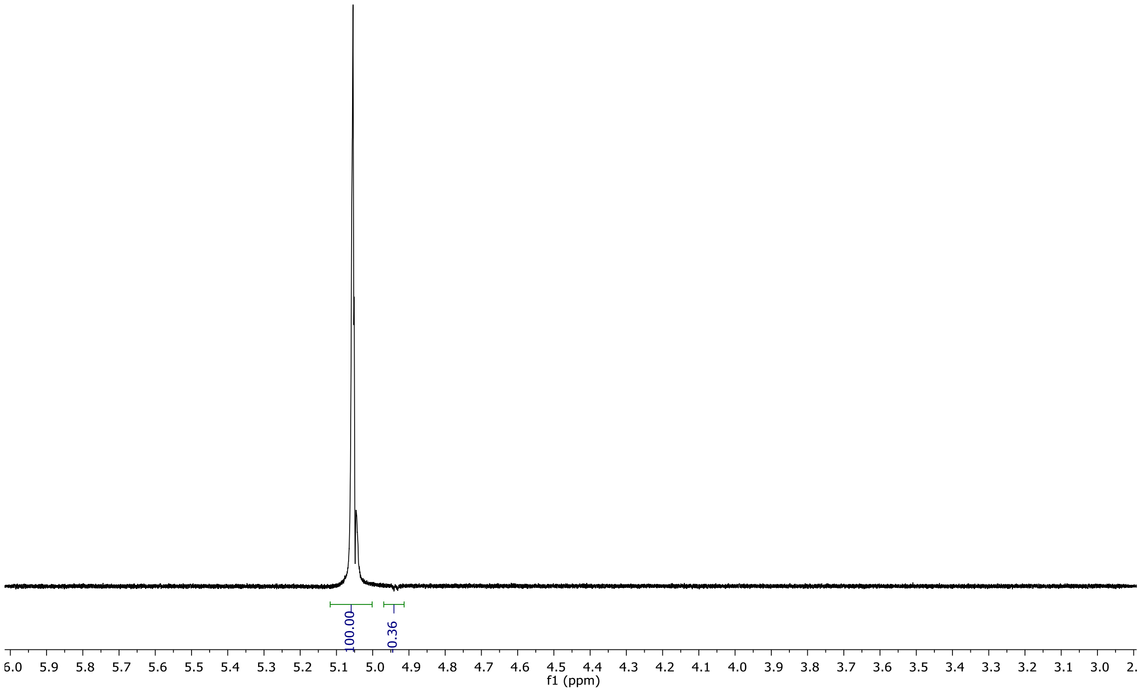

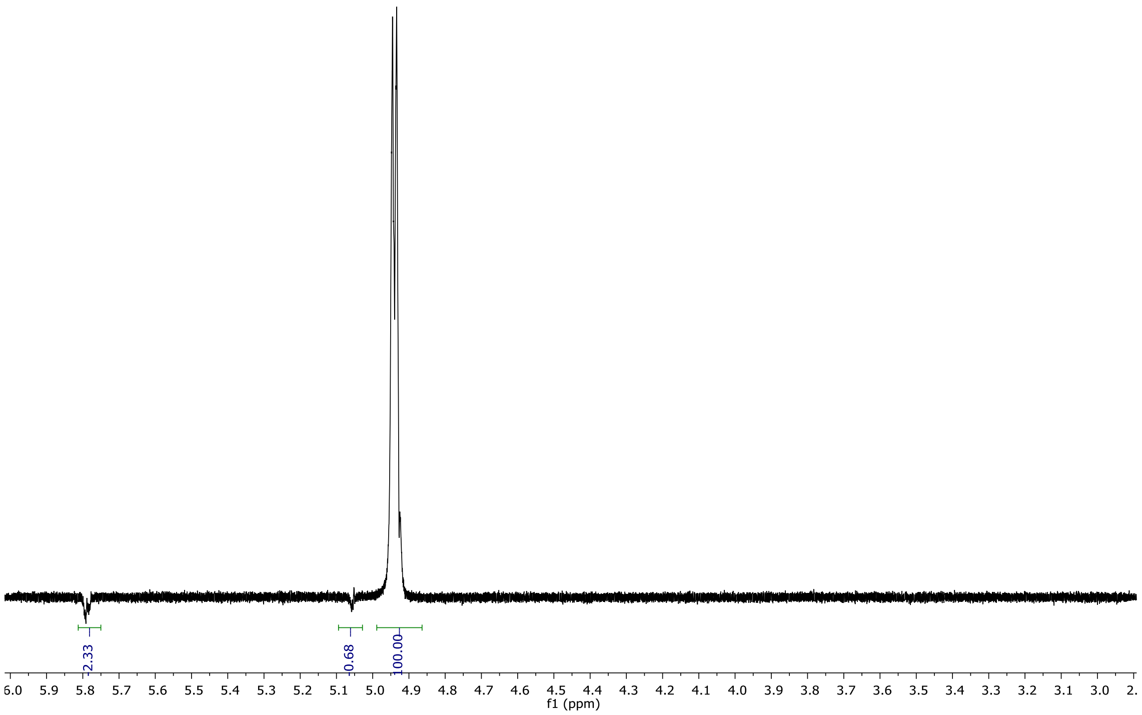
2.33
0.68
100.00
6.0 5.9 5.8 5.7 5.6 5.5 5.4 5.3 5.2 5.1 5.0 4.9 4.8 4.7 4.6 4.5 4.4 4.3 4.2 4.1 4.0 3.9 3.8 3.7 3.6 3.5 3.4 3.3 3.2 3.1 3.0
f1 (ppm)

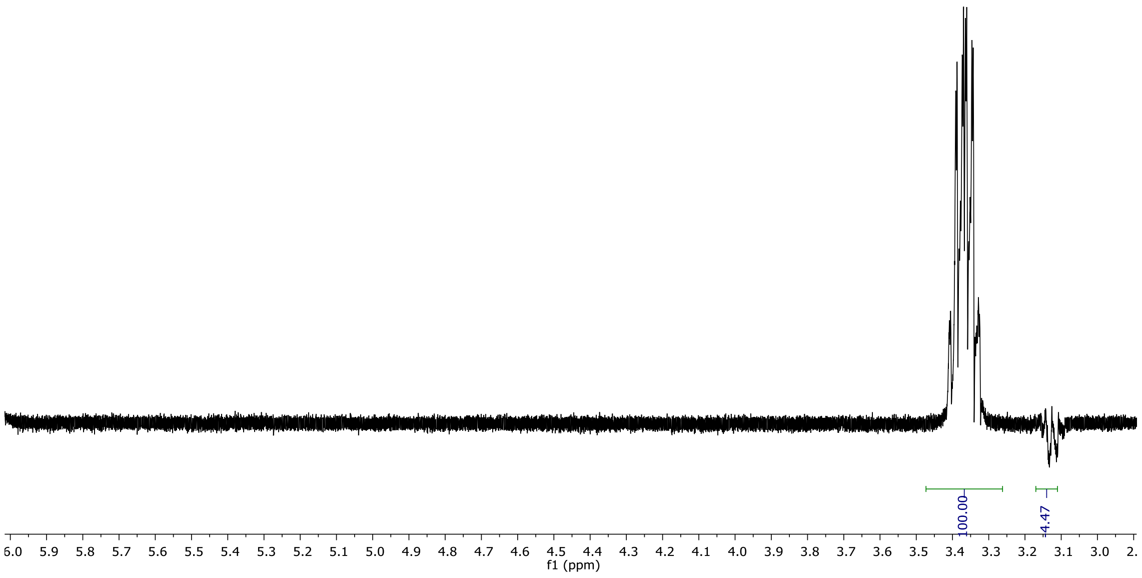
100.00
4.47
6.0 5.9 5.8 5.7 5.6 5.5 5.4 5.3 5.2 5.1 5.0 4.9 4.8 4.7 4.6 4.5 4.4 4.3 4.2 4.1 4.0 3.9 3.8 3.7 3.6 3.5 3.4 3.3 3.2 3.1 3.0
f1 (ppm)

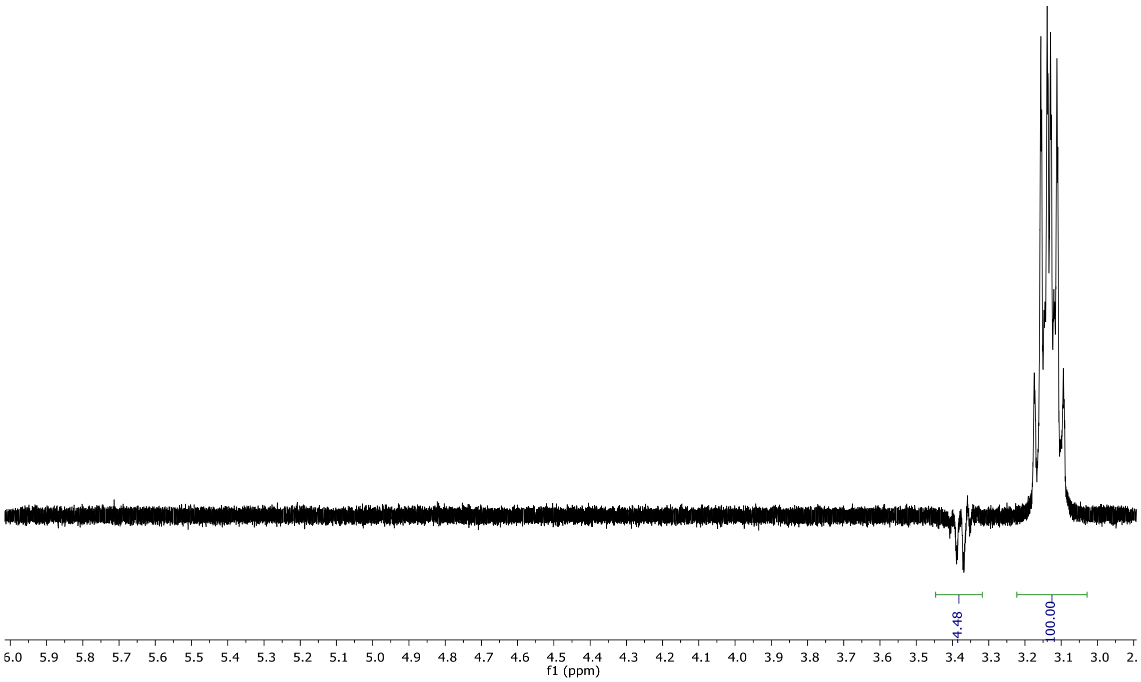
4.48
100.00
6.0 5.9 5.8 5.7 5.6 5.5 5.4 5.3 5.2 5.1 5.0 4.9 4.8 4.7 4.6 4.5 4.4 4.3 4.2 4.1 4.0 3.9 3.8 3.7 3.6 3.5 3.4 3.3 3.2 3.1 3.0
f1 (ppm)

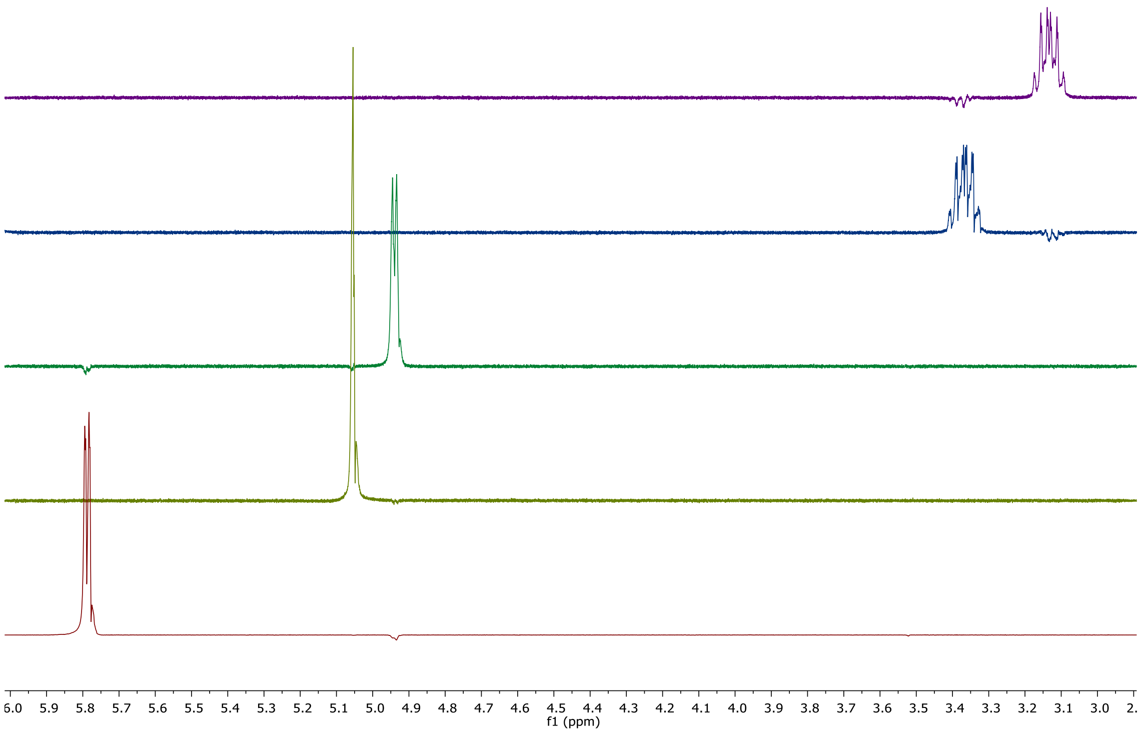
6.0 5.9 5.8 5.7 5.6 5.5 5.4 5.3 5.2 5.1 5.0 4.9 4.8 4.7 4.6 4.5 4.4 4.3 4.2 4.1 4.0 3.9 3.8 3.7 3.6 3.5 3.4 3.3 3.2 3.1 3.0
f1 (ppm)

7. Characterization by NMR Spectroscopy of products 25.

3-Methyl-5-phenyl-1-(*p*-tolyl)-3-(*p*-tolylamino)-1,3-dihydro-2*H*-pyrrol-2-one (25a).

^{1}H NMR (400 MHz, $CDCl_3$)

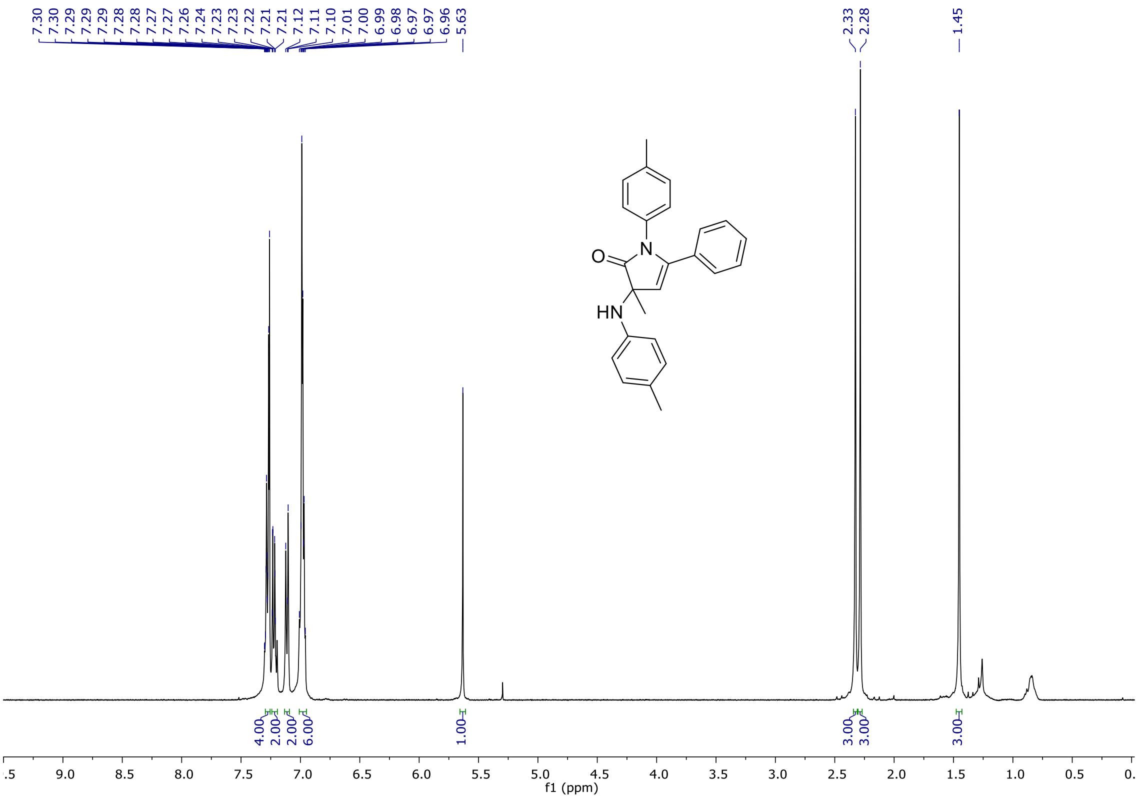

^{13}C NMR {^{1}H} (101 MHz, $CDCl_3$)

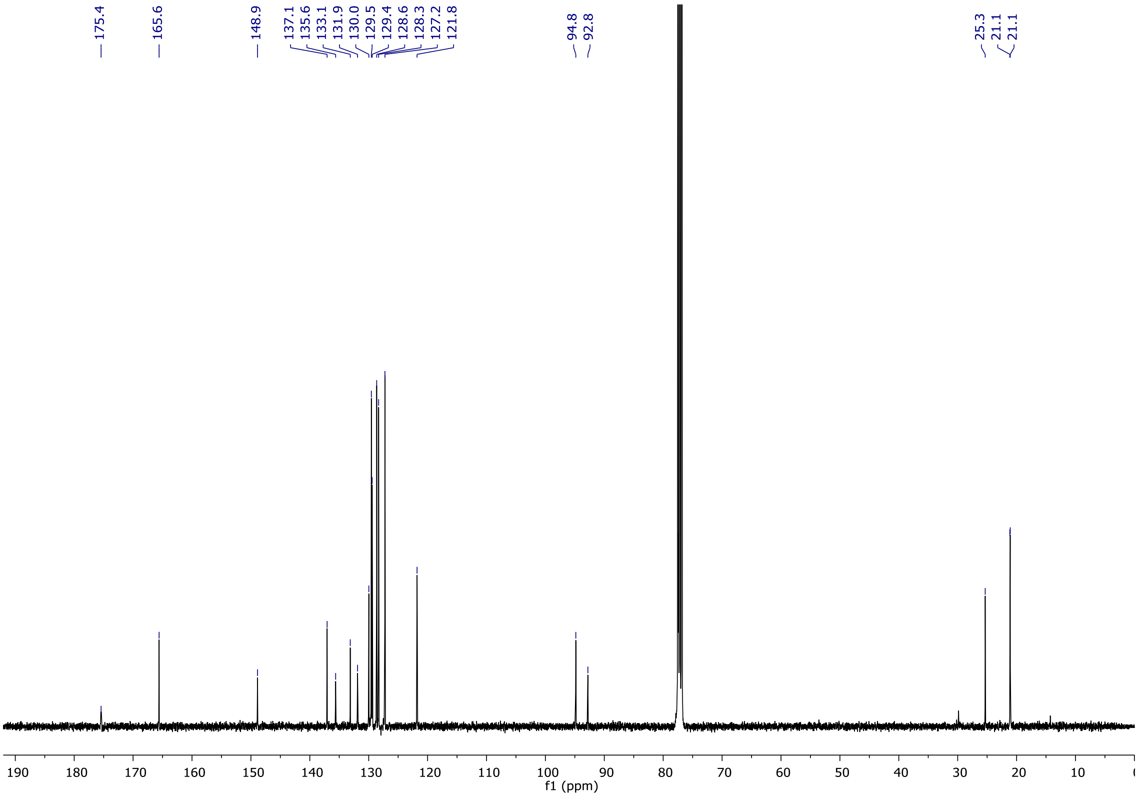

2D-COSY NMR {^{1}H – ^{1}H} (400 MHz, $CDCl_3$)

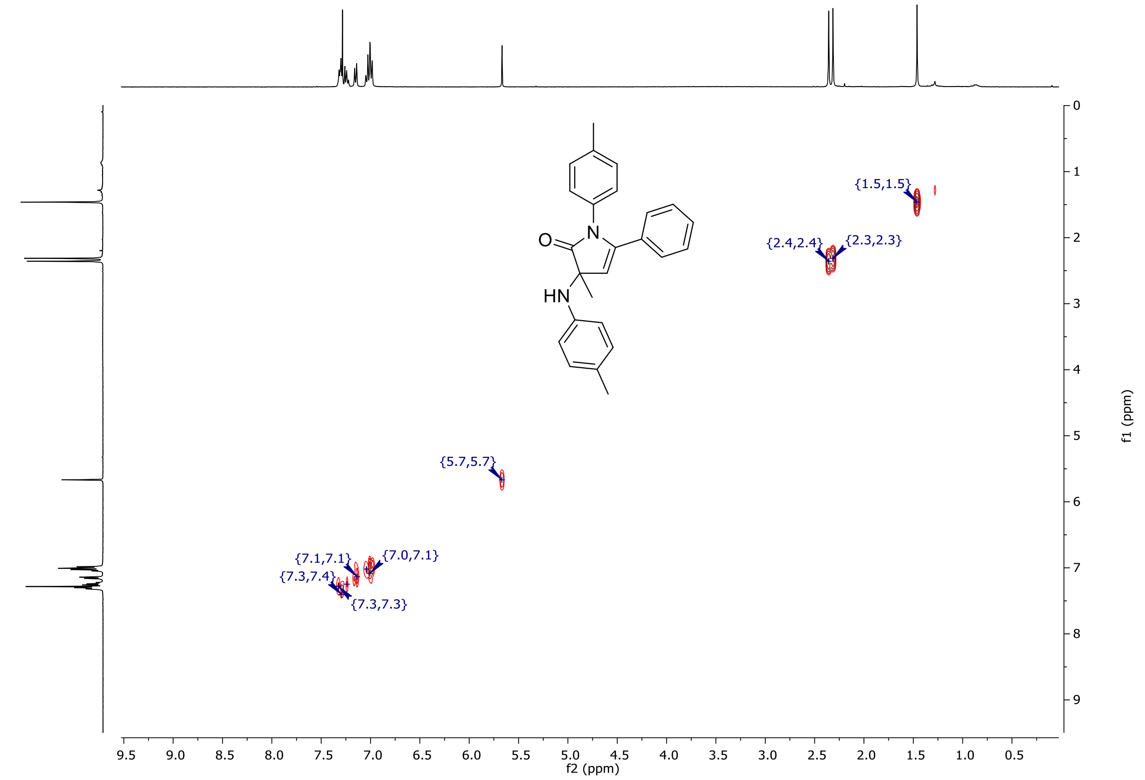

2D-HSQC NMR {^{1}H – ^{13}C} (^{1}H: 400 MHz, ^{13}C: 101 MHz, $CDCl_3$)

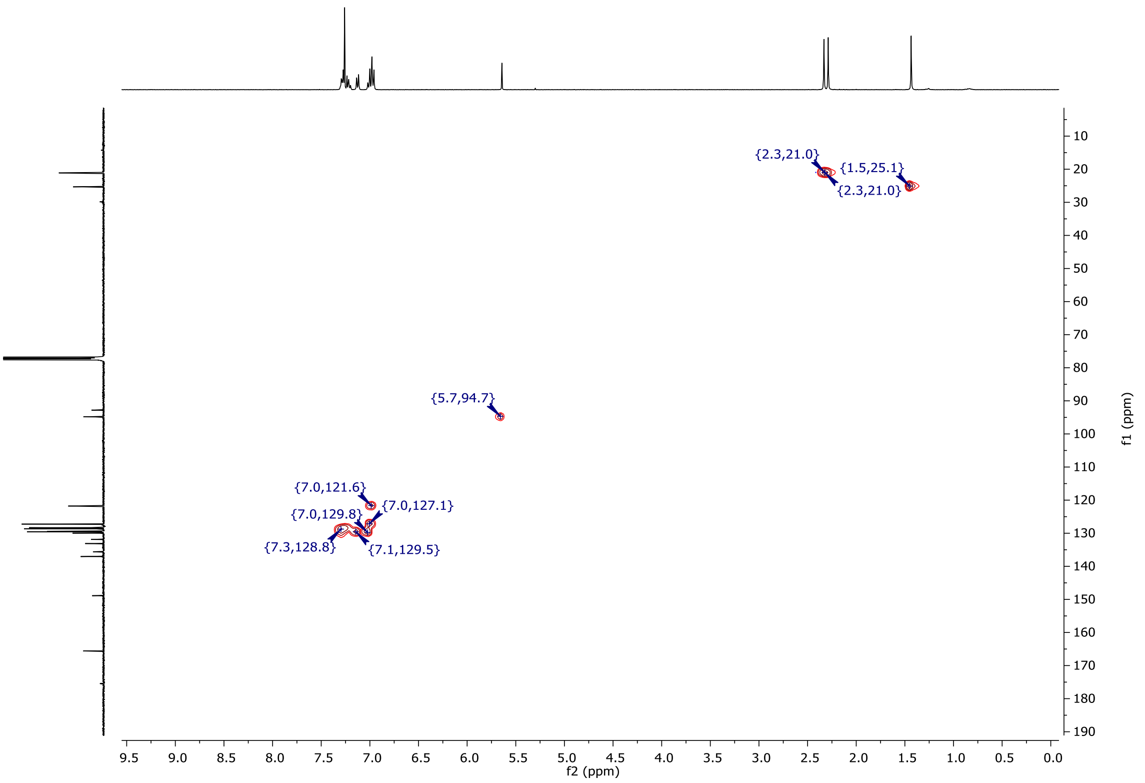

2D-HMBC NMR {$^{1}H – ^{13}C$} (^{1}H: 400 MHz, ^{13}C: 101 MHz, $CDCl_3$)

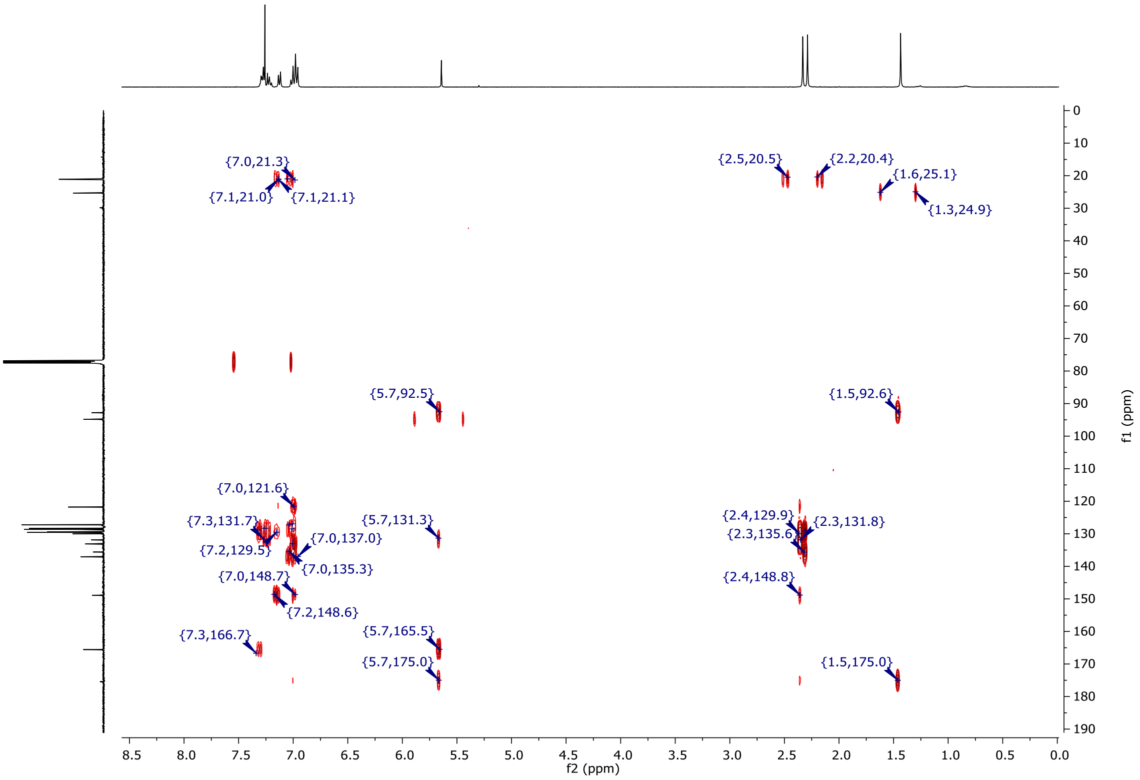

3-Butyl-5-phenyl-1-(*p*-tolyl)-3-(*p*-tolylamino)-1,3-dihydro-2*H*-pyrrol-2-one (25b).

^{1}H NMR (400 MHz, $CDCl_3$)

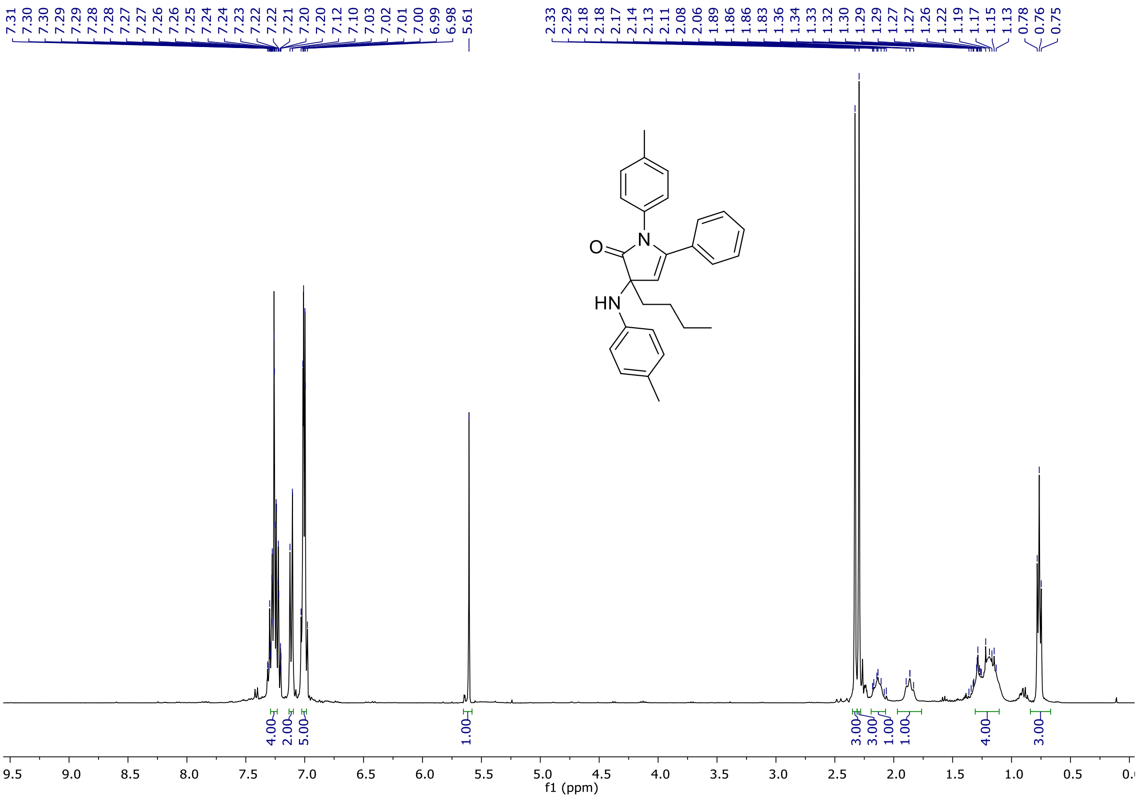

^{13}C NMR {^{1}H} (101 MHz, $CDCl_3$)

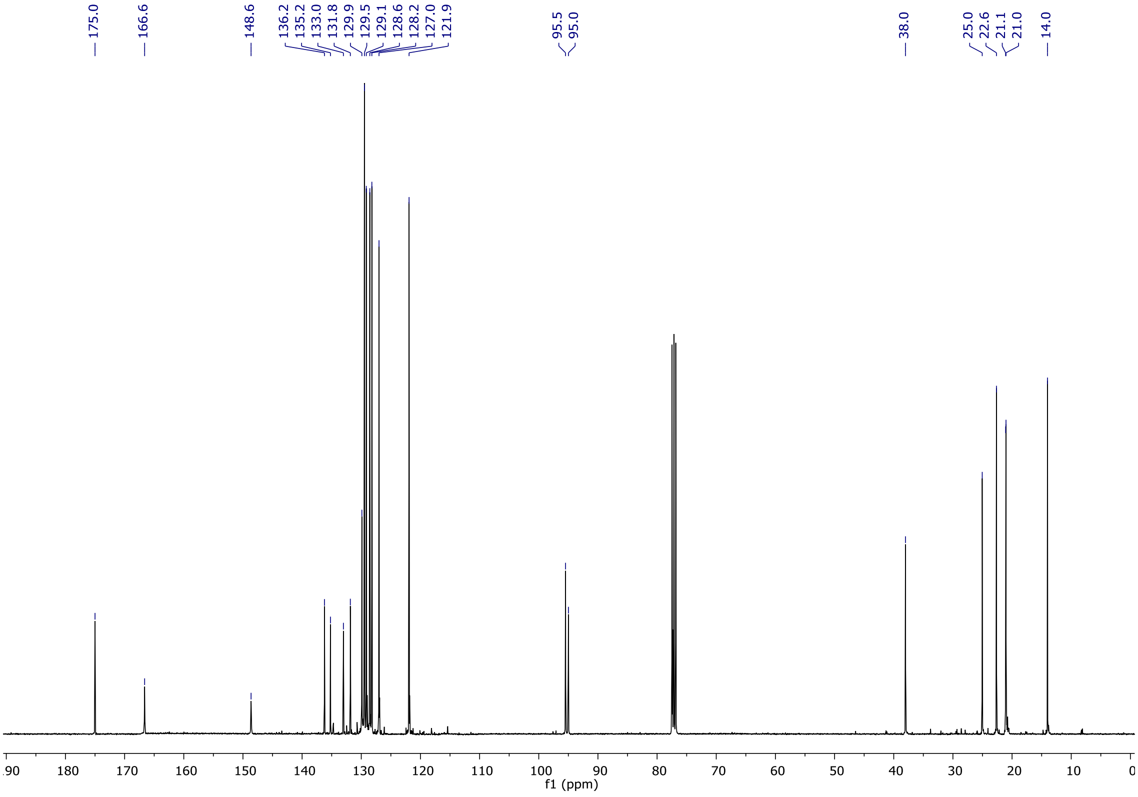

3-Butyl-1-(4-methoxyphenyl)-3-((4-methoxyphenyl)amino)-5-phenyl-1,3-dihydro-2*H*-pyrrol-2-one (25c).

^{1}H NMR (400 MHz, $CDCl_3$)

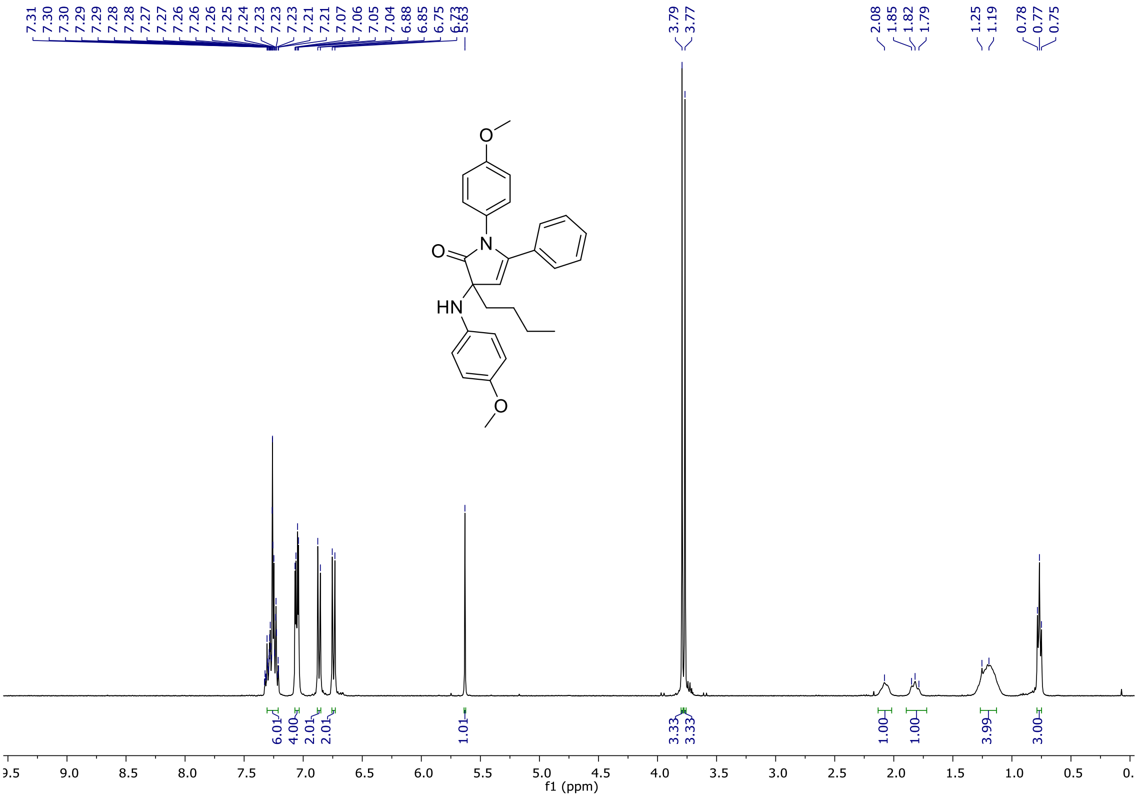

^{13}C NMR {^{1}H} (101 MHz, $CDCl_3$)

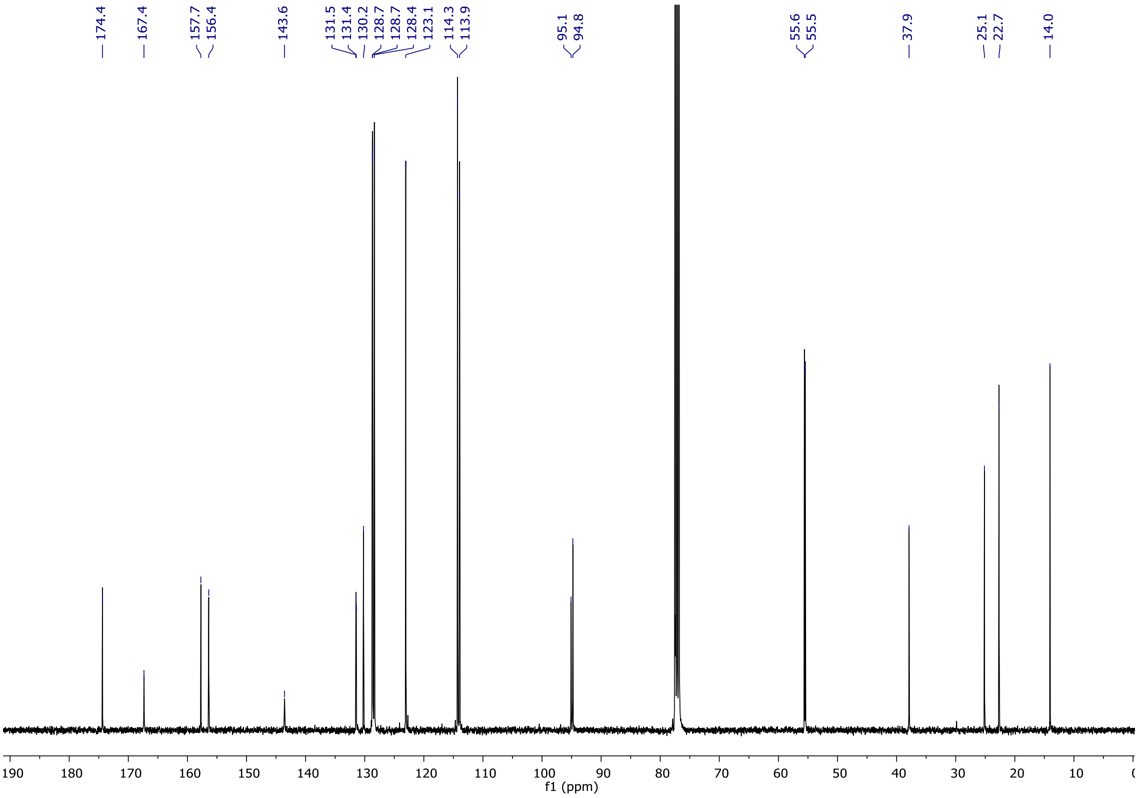

1-(4-Bromophenyl)-3-((4-bromophenyl)amino)-3-butyl-5-phenyl-1,3-dihydro-2*H*-pyrrol-2-one (25d).

^{1}H NMR (400 MHz, $CDCl_3$)

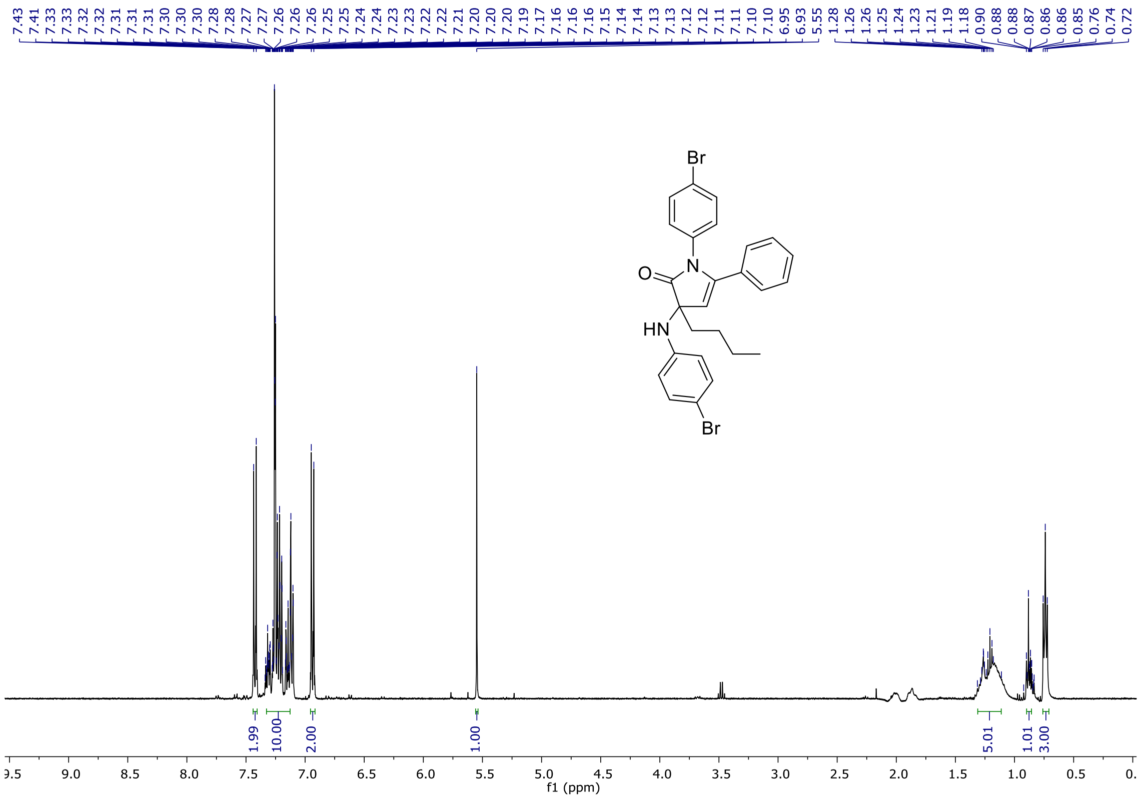

^{13}C NMR {^{1}H} (75 MHz, $CDCl_3$)

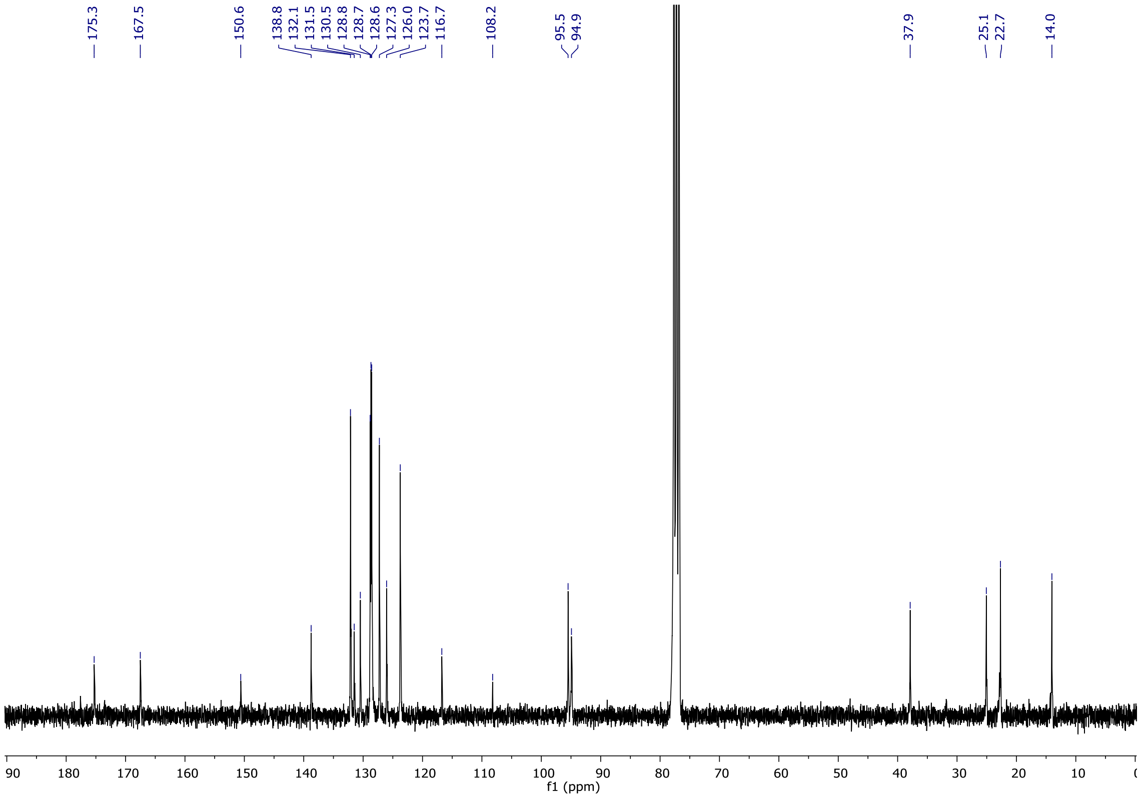

3-Butyl-1-(3-chlorophenyl)-3-((3-chlorophenyl)amino)-5-phenyl-1,3-dihydro-2*H*-pyrrol-2-one (25e).

^{1}H NMR (400 MHz, $CDCl_3$)

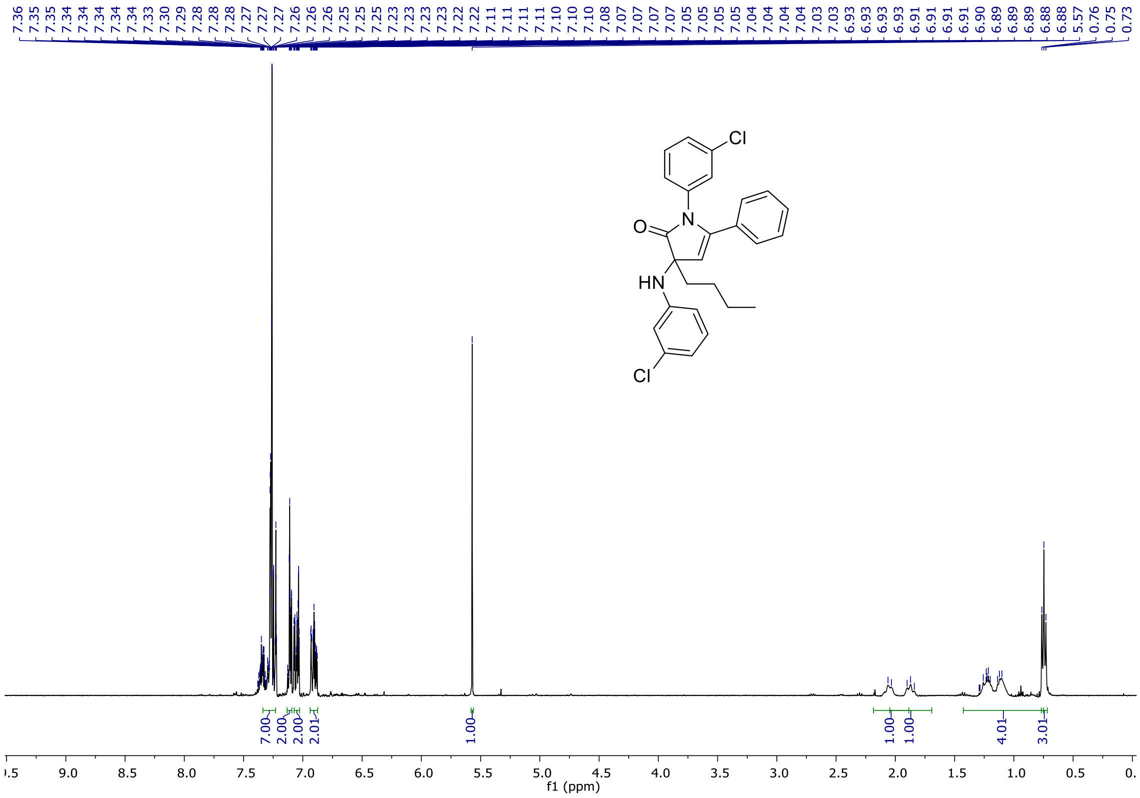

^{13}C NMR {^{1}H} (101 MHz, $CDCl_3$)

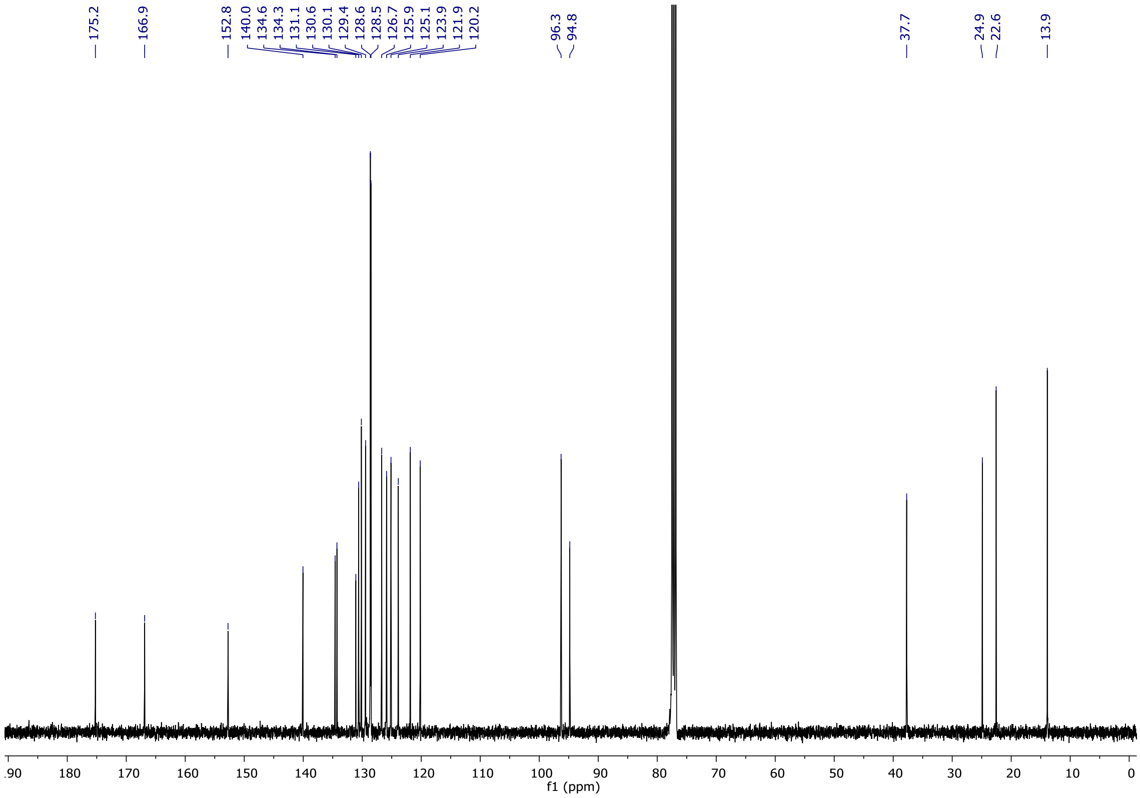

8. Calculation of Lipinski's rule of five and prediction of ADME properties.

In the following table some calculations had been developed to predict ADME parameters, and determine the medicinal chemistry friendliness of our small molecules. Lipinski rules are used to evaluate if a compound is likely to be an orally active drug in humans. According to Lipinski's rule, an orally active drug has no more than one violation of the following criteria: MW ≤ 500, LogP ≤ 4.15, N or O atoms ≤ 10 and NH or OH atoms ≤ 5.[7] These calculations were performed using SwissADME program from the Swiss Institute of Bioinformatics (http://www.swissadme.ch/).

Comp.	MW (g/mol)	LogP	HBA	HBD	RB	TPSA (Å^2)	Lipinski (Violations)	GI Abs.	BBB Permeation
**7e**	404.50	4.25	3	1	7	58.64	Yes (0)	High	Yes
**12a**	516.52	3.27	7	2	12	124.21	Yes (1)	High	No
**12b**	580.61	4.60	5	2	10	105.75	Yes (1)	High	No
**25a**	368.47	4.73	1	1	4	32.34	Yes (1)	High	Yes
**25b**	410.55	5.74	1	1	7	32.34	Yes (1)	High	No
**25c**	442.55	5.03	3	1	9	50.80	Yes (0)	High	Yes
**25d**	540.29	6.33	1	1	7	32.34	No (2)	Low	No
**25e**	451.39	6.15	1	1	7	32.34	Yes (1)	Low	No
**26**	278.35	3.26	1	1	3	32.34	Yes (0)	High	Yes

[7] Lipinski, C.A.; Lombardo, F.; Dominy, B. W.; Feeney, P. J. Experimental and computational approaches to estimate solubility and permeability in drug discovery and development settings. *Adv. Drug. Deliv. Rev.* **2001**, *64*, 4-17.
